# Quantum Chemical and Kinetic Study on Radical/Molecule Formation Mechanism of Pre-Intermediates for PCTA/PT/DT/DFs from 2-Chlorothiophenol and 2-Chlorophenol Precursors

**DOI:** 10.3390/ijms20071542

**Published:** 2019-03-27

**Authors:** Chenpeng Zuo, Hetong Wang, Wenxiao Pan, Siyuan Zheng, Fei Xu, Qingzhu Zhang

**Affiliations:** 1 Environment Research Institute, Shandong University, Qingdao 266237, China; zuochenpeng@126.com (C.Z.); Kishi_Wang@163.com (H.W.); zhengsiyuan1991@126.com (S.Z.); zqz@sdu.edu.cn (Q.Z.); 2 State Key Laboratory of Environmental Chemistry and Ecotoxicology, Research Center for Eco-Environmental Sciences, Chinese Academy of Sciences, Beijing 100085, China; wxpan@rcees.ac.cn; 3 Shenzhen Research Institute, Shandong University, Shenzhen 518057, China

**Keywords:** PCTA/PT/DTs, formation mechanism, radical/molecule coupling, density functional theory, rate constant

## Abstract

Polychlorinated phenoxathiins (PCPTs), polychlorinated dibenzothiophenes (PCDTs), and polychlorinated thianthrenes (PCTAs) are sulfur analogues of polychlorinated dibenzo-p-dioxins and polychlorinated dibenzofurans (PCDD/DFs). Chlorothiophenols (CTPs) and chlorophenols (CPs) are key precursors for the formation of PCTA/PT/DTs, which can react with H or OH to form chloro(thio)phenoxy radical, sulfydryl/hydroxyl-substituted phenyl radicals, and (thio)phenoxyl diradicals. However, previous radical/radical PCTA/DT formation mechanisms in the literature failed to explain the higher concentration of PCDTs than that of PCTAs under the pyrolysis or combustion conditions. In this work, a detailed thermodynamics and kinetic calculations were carried out to investigate the pre-intermediate formation for PCTA/PT/DTs from radical/molecule coupling of the 2-C(T)P with their key radical species. Our study showed that the radical/molecule coupling mechanism explains the gas-phase formation of PCTA/PT/DTs in both thermodynamic and kinetic perspectives. The S/C coupling modes to form thioether-(thio)enol intermediates are preferable over the O/C coupling modes to form ether-(thio)enol intermediates. Thus, although the radical/molecule coupling of chlorophenoxy radical with 2-C(T)P has no effect on the PCDD/PT formation, the radical/molecule coupling of chlorothiophenoxy radical with 2-C(T)P plays an important role in the PCTA/PT formation. Most importantly, the pre-PCDT intermediates formation pathways from the couplings of sulfydryl/hydroxyl-substituted phenyl radical with 2-C(T)P and (thio)phenoxyl diradicals with 2-C(T)P are more favorable than pre-PCTA/PT intermediates formation pathways from the coupling of chlorothiophenoxy radical with 2-C(T)P, which provides reasonable explanation for the high PCDT-to-PCTA ratio in the environment.

## 1. Introduction

Polychlorinated thianthrenes/dibenzothiophenes (PCTA/DTs), which are polychlorinated dibenzo-p-dioxins/dibenzofurans (PCDD/DFs) analogues with all (or at least one) oxygen atoms substituted by the sulfur atoms, have given rise to environmental and regulatory concerns to the public. PCTA/DTs are found as mixtures of 75 PCTA and 135 PCDT isomers, which have similar geochemical behavior, toxicity, persistence, lipophilic, and physicochemical properties as PCDD/DFs [1,2,3,4,5,6,7]. PCTA/DTs have been widely detected in the different environmental samples, such as aquatic organisms [8], soil and sediment [9,10], pulp bleaching [11], wastes from petroleum refineries [12], petroleum spills [13], especially in some high-temperature pyrolysis or combustion conditions such as incineration of municipal waste and fly ash [14,15], stack gas [16], and the metal reclamation industry [17]. The concentrations of PCDTs in fly ash and in stack gas samples are much higher than those in some pulp mill effluents [18]. Sinkkone et al. reported the concentrations of TCDBTs, TeCDBTs, and PeCDBTs in gas phase samples from the aluminum smelter to be in the range of 40–850 ng/nm^3^, 9–370 ng/nm^3^, and 1–155 ng/nm^3^, respectively, and the amount approached the concentration of PCDD/DFs in emissions and wastes [17]. Similar to PCDD/DFs, PCTA/DTs were never intentionally synthesized for commercial purposes, but formed as byproducts from the thermal and combustion processes. Polychlorinated phenoxathiins (PCPTs) are also one group of chlorinated tricyclic aromatic heterocycles which can be identified as PCDD analogues with one oxygen substituted by sulfur, or PCTAs with one sulfur substituted by oxygen. It is reported that PCPTs have thermodynamic properties, persistence, and environmental mobility, as do PCTA/DTs, which can be viewed as dioxin-like compounds [19]. Although the literature on the formation of PCPTs are less than that on PCDD/DFs and PCTA/DTs, the similarity in structural and chemical properties of PCPTs to PCDD/DFs and PCTA/DTs reveals their possibly similar formation pathways. For example, PCPTs have been proved to form with PCTAs and PCDTs in combustion experiments [20]. Ferrario et al. stated that phenoxathiins can be converted to dibenzofurans by heating with metallic copper at 250 °C [21]. Hence, the information about formation mechanism of PCTA/PT/DTs under combustion and thermal processes are required, which can serve as the basis for reducing PCTA/PT/DTs emissions and human or environmental exposure. 

Careful examination of the literature shows that two heterogeneous PCTA/DT formation pathways were proposed: Reactions from elemental carbon, sulfur, and chlorine- and sulfur-containing pesticides [22], and reactions from chemical precursors [23,24,25,26,27], which is the most direct route to the formation of PCTA/DTs. Among different precursors of PCTA/DTs, chlorothiophenols (CTPs) are demonstrated to be the most important precursors of PCTA/DTs, which is consistent with the widely recognized fact that chlorophenols (CPs) are identified to be predominant precursors of PCDD/DFs [23,24,28]. CTPs are used in large quantities in various chemical industries, such as the manufacturing of dyes, insecticides, printing inks, pharmaceuticals, and polyvinyl chloride [29], while CPs are released from direct application as biocides, leaching from wood products, synthesis during bleaching operations, and emissions from operating facilities [30,31]. Under high-temperature conditions, CTPs can readily form chlorothiophenoxy radical (CTPRs), sulfydryl-substituted phenyl radical, and thiophenoxyl diradical by losing the sulfydryl-H, the H/Cl atom combined to the carbon in the adjacent position of the carbon with -SH group, and both the sulfydryl-H and the ortho-substituted H/Cl, respectively, via abstraction reactions by H, OH, Cl, or O(^3^P). Although sulfydryl-substituted phenyl radicals and thiophenoxyl diradicals have not yet been detected in combustion and thermal processes, their oxygenated counterparts hydroxyl-substituted phenyl radicals and phenoxyl diradicals have been identified and proposed to be potential precursors for PCDD/DF formation [32,33,34,35]. In their theoretical studies, Yu et al. and Pan et al. proved the formation feasibility of sulfydryl-substituted phenyl radicals and thiophenoxyl diradicals from CTPs and the formation feasibility of hydroxyl-substituted phenyl radicals and phenoxyl diradicals from CPs, and also proved the corresponding energetically favorable contributions to the formation of PCTA/DT and PCDD/DFs, respectively [27,36].

Similar to the formation of PCDD/DFs from CPs precursor, the homogeneous gas-phase formation of PCTA/DTs involves radical/radical condensation of CTPRs and radical/molecule recombination of CTPR and CTP [23,24,25,37,38,39,40]. Dar et al. presented that radical/radical couplings are thermodynamically comparable to radical/molecule recombinations for the PCTA/DT formation [23,24]. Therefore, following the radical/radical routes, a series of theoretical studies on PCTA/DT formation mechanisms from the coupling of 2-CTPRs, 2,4,5-TCTPRs, 2,4-DCTPRs, and 2,4,6-TCPRs were carried out by Dar et al. and our group [23,24,25,26]. These studies showed that the formation of PCTAs was easier than that of PCDT due to the fact that the PCTA can form via one elementary step less than PCDT and the potential barrier of the rate-determining step of PCTA is about 10 kcal/mol lower than that of PCDT [23,24,25,26]. However, this conclusion failed to give reasonable explanation for the much higher concentration of PCDTs than that of PCTAs under the pyrolysis or combustion conditions [16,17]. For instance, the concentration of tetrachlorobenzothiophene (TeCDT) was detected to be approximately 5–40 times greater than that of tetrachlorothianthrene (TeCTA) in stack gas samples from waste incineration samples [16]. Sinkkone et al. observed that the mean concentrations of trichlorobenzothiophene (TCDT) (8.944 ng/g) in some ash and slag samples was much higher than that of triachlorothianthrene (TCTA) (0.052 ng/g) [17]. This great discrepancy prompted us to turn our attention back to the radical/molecule mechanism, which has long been overlooked in the PCTA/DT formation and may contribute to the high PCDT-to-PCTA ratio. First, the radical/molecule mechanism from 2-CTPR with 2-CTP, which is calculated using B3LYP method by Dar et al. [23,24] has ignored the electron correlation and may overestimate the energy values. Second, a recent study by Pan et al. showed that the radical/molecule mechanisms from phenyl radicals and phenoxyl diradicals with 2-CP solely lead to the formation of PCDF, which well accounts for the experimental observation of the high PCDF-to-PCDD ratio from CPs as precursors in gas-phase and particle-mediated conditions [41]. Along the same line of inquiry, the radical/molecule mechanisms from sulfydryl-substituted phenyl and thiophenoxyl diradicals with CTPs were inspired to be further studied and compared with the oxygen-substituted reactions. Third, considering the structure and property similarity of PCPTs with PCTA/DDs and the coexistence of CTPs and CPs, it is of significance to study the PCPT formation from cross-condensation of CPs with CTP-related radicals or CTPs with CP-related radicals. High correlation between the PCTA/DTs, PCDD/DFs, and PCPTs revealed their similar formation mechanism [14,20,21,42,43]. As far as we know, there is no information available on the radical/molecule mechanisms for the PCTA/DT formation from sulfydryl-substituted phenyl and thiophenoxyl diradicals with CTPs, as well as the radical/molecule mechanisms for the PCPT formation from CP and CTP as precursors.

**Figure 1:**
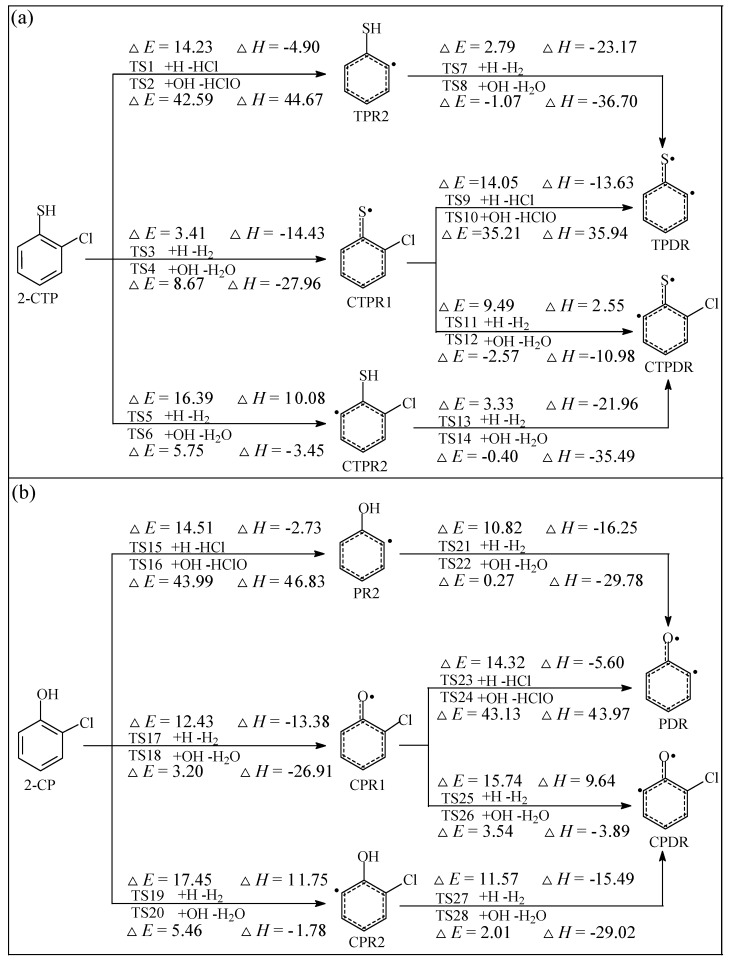
Formation of radical species C(T)PR1, C(T)PR2, C(T)PDR, (T)PR2, and (T)PDR embedded with the potential barriers Δ*E* (in kcal/mol) and reaction heats Δ*H* (in kcal/mol) from reactions of 2-CTP (**a**) and 2-CP (**b**) with H/OH radicals, respectively. Δ*H* is calculated at 0 K.

Therefore, in this study, we present a systematic theoretical investigation on the initial pathways of PCTA/PT/DT formation from the condensation reactions of 2-CP/2-CTP with chlorinated phenoxy radical 2-chlorophenoxy (CPR1) and chlorinated thiophenoxy radical 2-chlorothiophenoxy (CTPR1), phenyl radicals 2-hydroxylphenyl (PR2) and 2-sulfydrylphenyl (TPR2), chlorinated phenyl radicals 2-hydroxyl-3-chloro-phenyl (CPR2) and 2-sulfydryl-3-chloro-phenyl (CTPR2), phenoxyl diradical (PDR) and thiophenoxyl diradical (TPDR), and chlorinated phenoxyl diradical (CPDR) and chlorinated thiophenoxyl diradical (CTPDR) (shown in Figure 1). 2-CTP and 2-CP are selected as model compounds because they are the simplest and most representative CTPs and CPs precursors to produce PCTA/PT/DT/DFs. The kinetic data and rate constants were evaluated over a wide temperature range of 600–1200 K and fitted into Arrhenius formulas to improve and optimize PCTA/DT formation mathematic models. The temperature range covers all the possible temperature range for the formation of PCTA/PT/DTs at high temperature pyrolysis or combustion conditions. This work provides a new mechanism for the formation of PCTA/DTs to explain the higher concentration of PCDTs than that of PCTAs in the environment and explores plausible mechanism for the formation of dioxin-like compounds PCPTs. The contribution to PCTA/PT/DT/DF formation from different radical/molecule condensation reactions was sorted and compared with radical/radical mechanism from self-coupling of 2,4-dichlorothiophenoxys (2,4-DCTPRs) in our previous work [26] and the formation of PCDD/DFs from oxygen-substituted precursors [41].

## 2. Results

### 2.1. Formation of Radical Species C(T)PR1, C(T)PR2, C(T)PDR, (T)PR2, and (T)PDR from 2-CTP and 2-CP Molecules

**Figure 2:**
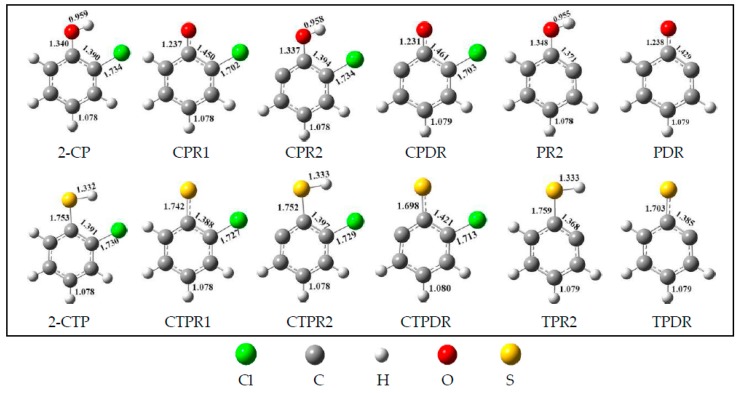
Optimized structures of 2-CP and 2-CTP molecules and related radicals in this paper: 2-chlorophenol (2-CP), 2-chlorophenoxy (CPR1), 2-hydroxyl-3-chloro-phenyl (CPR2), chlorinated phenoxyl diradical (CPDR), 2-hydroxylphenyl radical (PR2), phenoxyl diradical (PDR), 2-chlorothiophenol (2-CTP), 2-chlorothiophenoxy (CTPR1), 2-sulfydryl-3-chloro-phenyl (CTPR2), chlorinated thiophenoxyl diradical (CTPDR), 2-sulfydrylphenyl radical (TPR2), thiophenoxyl diradical (TPDR). All the values are in Å.

The formation of 2-chlorothiophenoxy (CTPR1), 2-sulfydryl-3-chloro-phenyl (CTPR2), chlorinated thiophenoxyl diradical (CTPDR), 2-sulfydrylphenyl radical (TPR2), and thiophenoxyl diradical (TPDR) derived from 2-chlorothiophenol (2-CTP), and the formation of 2-chlorophenoxy (CPR1), 2-hydroxyl-3-chloro-phenyl (CPR2), chlorinated phenoxyl diradical (CPDR), 2-hydroxylphenyl radical (PR2), and phenoxyl diradical (PDR) derived from 2-chlorophenol (2-CP) are the initial and key steps in the radical/molecule formation of PCTA/PT/DT/DFs. In combustion and thermal processes, these radicals may be generated by means of H or Cl extraction reactions of 2-C(T)P by the active radicals H, OH, Cl, or O(^3^P) which exist abundantly in high-temperature conditions. The potential barriers (Δ*E*) and the reaction heat (Δ*H*) which are calculated at the MPWB1K/6-311+G(3df,2p)//MPWB1K/6-31+G(d,p) level for the formation of C(T)PR1, C(T)PR2, C(T)PDR, (T)PR2, and (T)PDR from 2-C(T)P abstracted by the H and OH are given in the Figure 1a,b. In Figure 1, data of sulfydryl/hydroxyl-H abstraction of 2-C(T)P by H and OH are cited from our previous studies at the MPWB1K/6-311+G(3df,2p)//MPWB1K/6-31+G(d,p) level [44,45,46]. Pan et al. and Yu et al. also studied the formation of C(T)PR1, C(T)PR2, C(T)PDR, (T)PR2, and (T)PDR from 2-C(T)P molecules abstracted by H radical at the BB1K/6-311+G(3df,2p)//BB1K/6-311G(d,p) level [27,41]. Similar abstraction reactions by OH radical are added in this study. Notably, our value at the MPWB1K/6-311+G(3df,2p)//MPWB1K/6-31+G(d,p) level match well with Pan and Yu’s data at the BB1K/6-311+G(3df,2p)//BB1K/6-311G(d,p) level, and the maximum relative error remains within 1.03 kcal/mol for potential barriers (Δ*E*) and less than 0.90 kcal/mol for reaction heats (Δ*H*). Based on these results, the same level of can be expected for other species involved in this study. As can be seen in Figure 1, the chlorinated phenoxy/thiophenoxy radicals C(T)PR1 arises from cleavage of sulfydryl S-H bond or hydroxyl O-H bond from the precursor 2-C(T)P. Moreover, the sulfydryl-substituted/hydroxyl-substituted phenyl radicals (T)PR2 and C(T)PR2 arise from the dissociation of the Cl or H atom combined to the carbon in the adjacent position of the carbon with the sulfydryl/hydroxyl group. Additionally, the phenoxyl/thiophenoxyl diradicals (T)PDR and C(T)PDR arise from the loss of both of the sulfydryl-H or hydroxyl-H and the *ortho*-substituted Cl or H atom. All the optimized geometries for 2-C(T)P and related radicals are shown in Figure 2.

### 2.2. Formation of Pre-Intermediates of PCTA/PT/DT/DFs via Radical/Molecule Coupling Reactions

**Figure 3:**
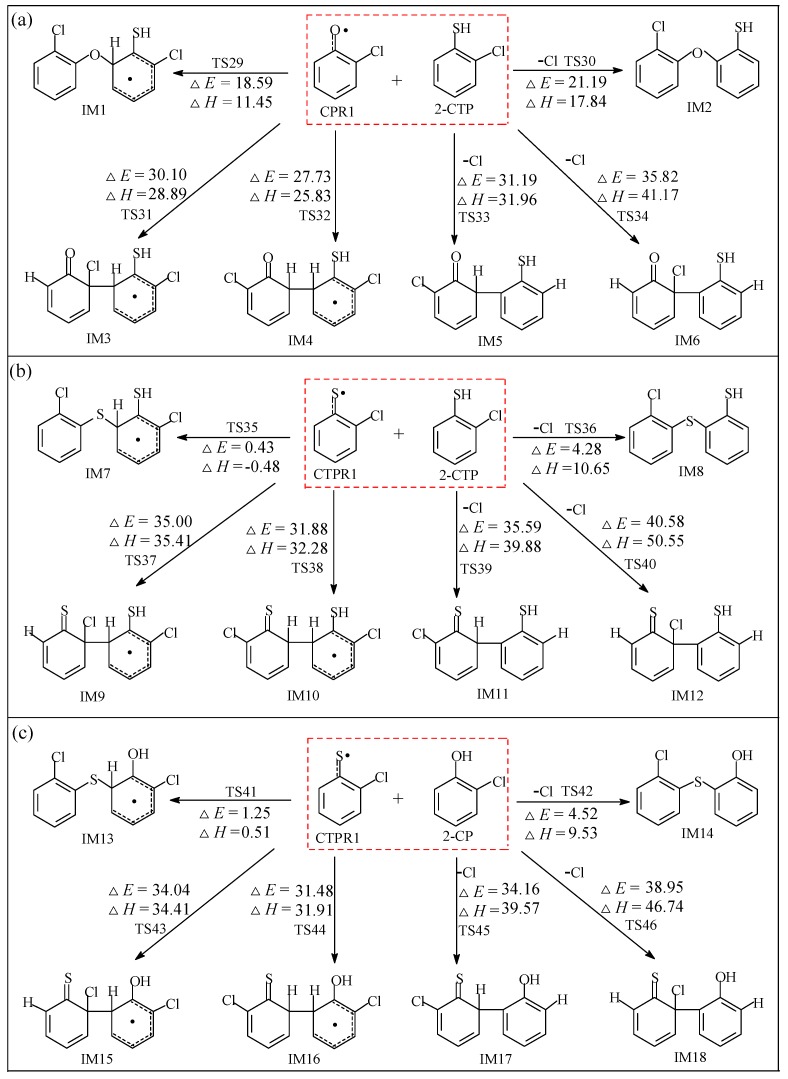
Pre-PCTA/PT/DT/DF formation routes embedded with the potential barriers Δ*E* (in kcal/mol) and reaction heats Δ*H* (in kcal/mol) from the cross-condensation reactions of CPR1 with 2-CTP (**a**), CTPR1 with 2-CTP (**b**), and CTPR1 with 2-CP (**c**). Δ*H* is calculated at 0 K.

**Figure 4:**
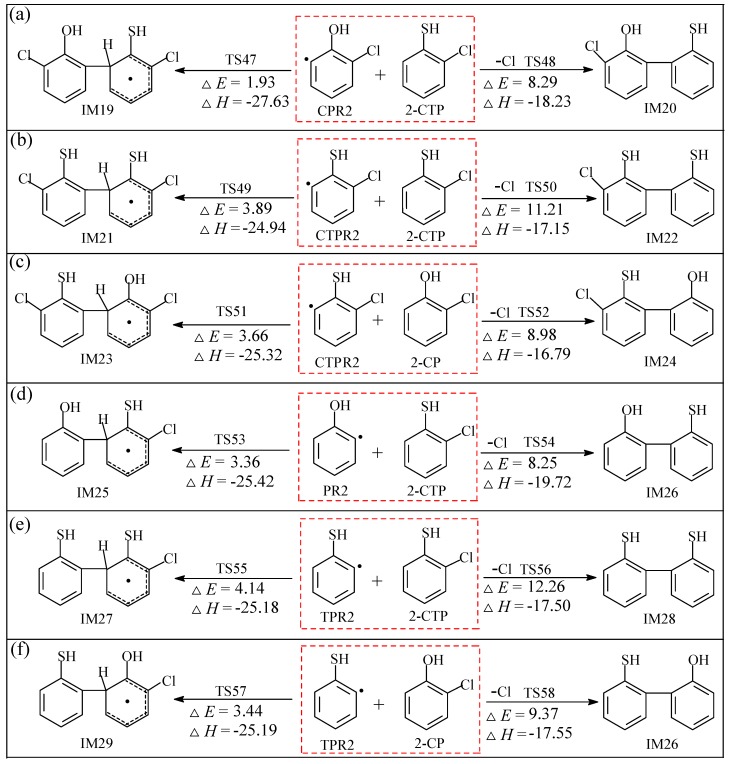
Pre-PCDT/DF formation routes embedded with the potential barriers Δ*E* (in kcal/mol) and reaction heats Δ*H* (in kcal/mol) from the coupled reactions of CPR2 with 2-CTP (**a**), CTPR2 with 2-CTP (**b**), CTPR2 with 2-CP (**c**), PR2 with 2-CTP (**d**), TPR2 with 2-CTP (**e**), and TPR2 and 2-CP (**f**). Δ*H* is calculated at 0 K.

**Figure 5:**
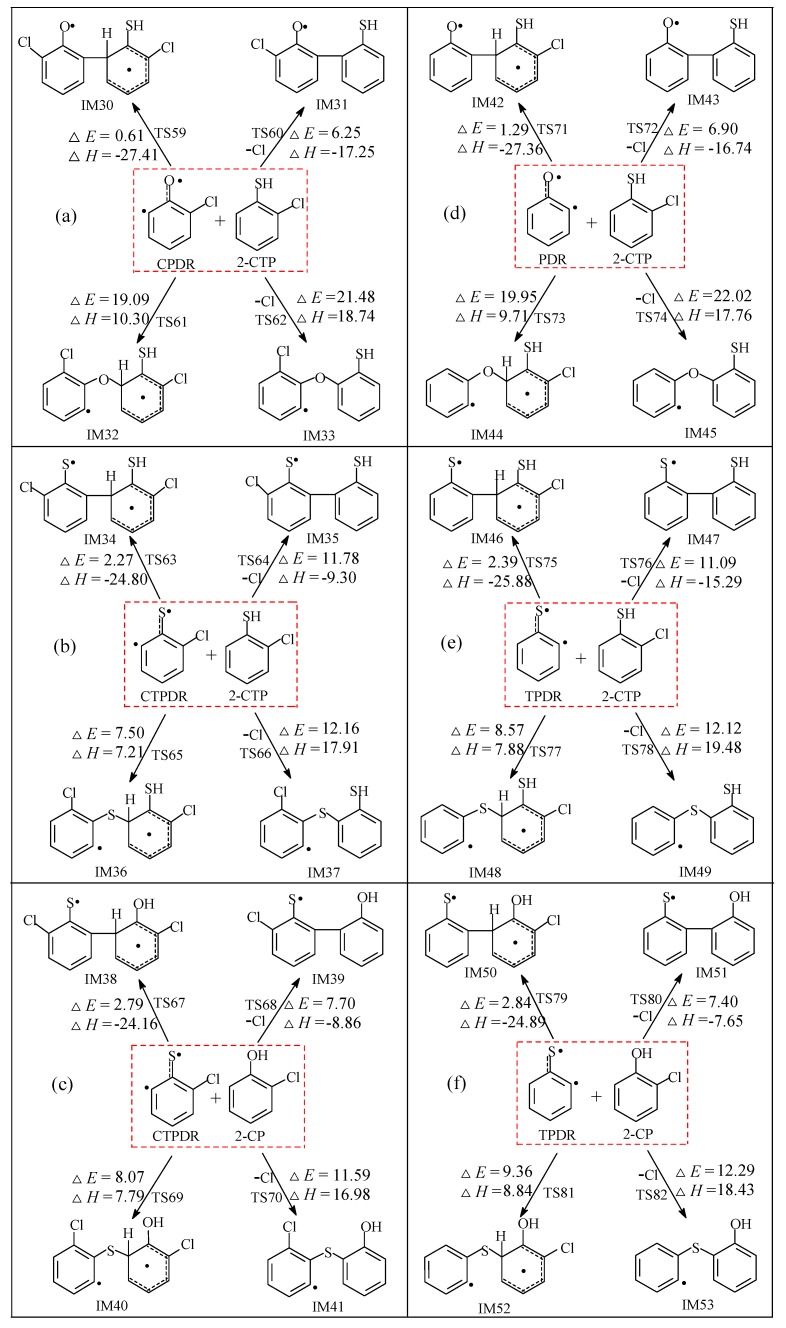
Pre-PCDT/DF formation routes embedded with the potential barriers Δ*E* (in kcal/mol) and reaction heats Δ*H* (in kcal/mol) from the coupled reactions of CPDR with 2-CTP (**a**), CTPDR with 2-CTP (**b**), CTPDR with 2-CP (**c**), PDR with 2-CTP (**d**), TPDR with 2-CTP (**e**), and TPDR and 2-CP (**f**). Δ*H* is calculated at 0 K.

The radical/radical or radical/molecule recondensation of C(T)P and C(T)PRs plays a crucial role in the homogeneous gas-phase formation of PCTA/PT/DTs. The radical/molecule coupling reaction routes via cross-condensation of 2-C(T)P molecule with chlorinated (thio)phenoxy radicals C(T)PR1, chlorinated or unchlorinated hydroxyl(sulfydryl)-substituted phenyl radicals C(T)PR2/(T)PR2, and chlorinated or unchlorinated (thio)phenoxyl diradicals C(T)PDR/(T)PDR are exhibited in Figure 3, Figure 4 and Figure 5, respectively. The potential barrier Δ*E* (in kcal/mol) and reaction heats Δ*H* (in kcal/mol) are calculated at the MPWB1K/6-311+G(3df,2p)//MPWB1K/6-31+G(d,p) level in Figure 3, Figure 4 and Figure 5. In order to compare with the PCDD/DF formation from 2-CP and corresponding oxygen-substituted radicals in Pan’s study [41], the radical/molecule coupling reaction routes via cross-condensation of 2-CP with chlorophenoxy radical CPR1, chlorinated or unchlorinated hydroxyl-substituted phenyl radicals CPR2/PR2, and phenoxyl diradicals CPDR/PDR are calculated at the MPWB1K/6-311+G(3df,2p)//MPWB1K/6-31+G(d,p) level and presented in Figure S1. Several typical optimized transition state geometries in PCTA/PT/DT/DF formation are shown in Figure S3.

#### 2.2.1. Coupling Reaction of C(T)PR1 with 2-C(T)P

In Figure 3, the cross-condensation reactions of CPR1 with 2-CTP, CTPR1 with 2-CTP, and CTPR1 with 2-CP are depicted in Figure 3a–c, respectively. They exhibit four kinds of sulfur/oxygen–carbon coupling modes (S/C or O/C for short): (1) The coupling of the phenolic oxygen with the *ortho* carbon bonded to hydrogen of 2-C(T)P molecule (O/σ-CH for short), (2) the coupling of the thiophenolic sulfur with the *ortho* carbon bonded to hydrogen of 2-C(T)P molecule (S/σ-CH for short), (3) the coupling of the phenolic oxygen with the *ortho* carbon bonded to chlorine of 2-C(T)P molecule (O/σ-CCl for short), and (4) the coupling of the thiophenolic sulfur with the *ortho* carbon bonded to chlorine of 2-C(T)P molecule (S/σ-CCl for short). In Figure 3, all the four coupling modes produce four (thio)ether-(thio)enol structures: IM1 are formed from the O/σ-CH coupling, IM2 are produced from O/σ-CCl coupling, IM7 and IM13 are generated from the S/σ-CH coupling, and IM8 and IM14 are shaped from the S/σ-CCl coupling. The O/σ-CH and S/σ-CH couplings are stepwise reactions, while the O/σ-CCl and S/σ-CCl couplings are one-step synergetic reactions accompanied by the Cl elimination. These intermediates are pre-intermediates for the formation of PCTA/PTs.

There are four kinds of carbon-carbon coupling modes (C/C for short) to produce 12 (thio)keto-(thio)enol adducts: (1) The coupling of carbon (hydrogen)-centered radical and carbon (hydrogen)-centered molecule (C_R_H/C_M_H for short), (2) the coupling of carbon (chlorine)-centered radical and carbon (hydrogen)-centered molecule (C_R_Cl/C_M_H for short), (3) the coupling of carbon (hydrogen)-centered radical and carbon (chlorine)-centered molecule (C_R_H/C_M_Cl for short), and (4) the coupling of carbon (chlorine)-centered radical and carbon (chlorine)-centered molecule (C_R_Cl/C_M_Cl for short). In Figure 3, the IM4, IM10, and IM16 are generated from C_R_H/C_M_H coupling; the IM3, IM9, and IM15 are formed from C_R_Cl/C_M_H coupling; the IM5, IM11, and IM17 are generated from the C_R_H/C_M_Cl coupling; and the IM6, IM12, and IM18 are built from the C_R_Cl/C_M_Cl coupling. The C_R_H/C_M_Cl and CCl_R_/CCl_M_ couplings are also accompanied by a synchronously elimination of Cl atom. These intermediates serve as building foundation for analogues of PCDT/DF structures.

#### 2.2.2. Coupling Reactions of C(T)PR2 and (T)PR2 with 2-C(T)P

The pre-PCDT/DF formation reaction pathways for cross-couplings of CPR2 and 2-CTP, CTPR2 and 2-CTP, CTPR2 and 2-CP, PR2 and 2-CTP, TPR2 and 2-CTP, and TPR2 and 2-CP embedded with the potential barriers Δ*E* (in kcal/mol) and reaction heats Δ*H* (in kcal/mol) are illustrated in Figure 4a–f, respectively. After the *ortho*-H/Cl abstraction in benzene ring of 2-CP and 2-CTP, they form the extremely high reactive carbon (radcial)-centered hydroxyl(sulfydryl)-substituted phenyl radicals [47,48], C(T)PR2 and (T)PR2, which may attack the 2-C(T)P molecule by two C/C coupling modes to form biphenyl intermediates: (1) The coupling of carbon (radical)-centered radical and carbon (hydrogen)-centered molecule (C•/CH for short), and (2) the coupling of carbon (radical)-centered radical and carbon (chlorine)-centered molecule (C•/CCl for short). The C•/CCl coupling is a synergetic reaction with the Cl elimination occurring at the same time. Six intermediates (IM19, IM21, IM23, IM25, IM27, and IM29) are produced by C•/CH coupling, and five other intermediates (IM20, IM22, IM24, IM26, and IM28) are produced by C•/CCl coupling. All 11 intermediates can result in the formation of PCDT/DFs, which are not the precursors for PCTA/PTs due to the absence of O/S centered radicals in C(T)PR2 and (T)PR2.

#### 2.2.3. Coupling Reactions of C(T)PDR and (T)PDR with 2-C(T)P

In this section, the condensation reaction pathways between CPDR and 2-CTP, CTPDR and 2-CTP, CTPDR and 2-CP, PDR and 2-CTP, TPDR and 2-CTP, and TPDR and 2-CP are discussed and the corresponding results are illustrated in Figure 5a–f, respectively. C(T)PDR and (T)PDR are diradicals with two typical radical sites located at the (thio)phenoxyl O or S atom and the *ortho* phenyl C atom. In Figure 5a,d, the O/C coupling is comprised of two modes (O/σ-CH and O/σ-CCl) to form ether-thioenol adducts and two kinds of C/C coupling modes(C•/CH and C•/CCl) to form biphenyl intermediates. In Figure 5b,c,e,f, there are two kinds of S/C coupling modes (S/σ-CH and S/σ-CCl) to form thioether-(thio)enol adducts and two kinds of carbon-carbon coupling modes(C•/CH and C•/CCl) to form thioketo-(thio)enol intermediates. The O/σ-CCl, S/σ-CCl and C•/CCl couplings are synergetic reactions accompanied by the Cl elimination occurring at the same time. The O/C or S/C condensations are endothermic reactions, and the C/C coupling are exothermic. As can be seen in Figure 5, the O/C coupling produces four species (IM32, IM33, IM44, and IM45), and the S/C coupling produces eight intermediates (IM36, IM37, IM40, IM41, IM48, IM49, IM52, and IM53), which would be followed by the abstraction of H, ring closure, and SH/OH elimination to form PCDT/DFs. The C/C coupling produces 12 species (IM30, IM31, IM34, IM35, IM38, IM39, IM42, IM43, IM46, IM47, IM50, and IM51) which would be followed by the abstraction of H, ring closure, and intramolecular elimination of SH/OH to form PCDT/DFs.

### 2.3. Rate Constant Calculations

The kinetic parameters of pre-PCTA/PT/DT/DF formation routes are significant to construct the formation kinetic model to predict the potential emission and harm to the environment. The rate constants of the formation of the pre-PCTA/PT/DT/DF intermediates from cross-condensation reactions of 2-C(T)P molecules with related radicals were calculated by using conventional transition state theory (TST) method with one-dimensional Wigner’s formalism contribution in the temperature range of 600–1200 K [49,50]. The calculated rate constants are shown in Table S2 of Supplementary Materials from 600–1200 K for every 100 K. In pyrolysis, combustion, and thermal processes, gas-phase PCDD/Fs can be formed by two main formation pathways, de novo synthesis and precursors synthesis. De novo synthesis proceeds through chlorination and oxidation of the macromolecular residual carbon between 600–900 K in the presence of oxygen. The precursors synthesis involves reaction of structurally relative precursors in the gas phase, at temperatures >900 K [51,52,53,54]. Furthermore, the concentration of PCDDs decreased rapidly because of splitting decomposition at high temperatures, >1200 K [55]. Similar to the structures of PCDD/DFs, PCTA/DTs may be formed by mechanisms that result in the formation of PCDD/Fs. So, we calculated the rate constants over a wide temperature range of 600–1200 K to compare crucial elementary reactions.

To be used more effectively, the rate constants were fitted, and Arrhenius formulas are given in Table 1 for the pre-PCTA/PT/DT/DF intermediates formation routes from cross-condensation of 2-C(T)P with C(T)PR1, C(T)PR2, and (T)PR2 radicals and Table 2 for the pre-PCDT/DF intermediates formation routes from cross-couplings of 2-C(T)P with C(T)PDR and (T)PDR radicals. The pre-exponential factor, the activation energy, and the rate constants can be obtained from these Arrhenius formulas. The average rate constant (1000 K) for the three coupling modes, i.e., for the O/C type, S/C type, and C/C type coupling reactions are listed in Table 3. In order to enrich these results, we calculated the activation enthalpies (∆*H*^≠^), activation Gibbs free energies (∆*G*^≠^), activation entropies (∆*S*^≠^), relative enthalpies (∆_r_*H*), relative Gibbs free energies (∆_r_*G*), and relative entropies (∆_r_*S*) at 298.15 K and under the pressure of 1 atm, as presented in Table S3.

## 3. Discussion

### 3.1. Formation of Radical Species C(T)PR1, C(T)PR2, C(T)PDR, (T)PR2, and (T)PDR from 2-CTP and 2-CP Molecules

From Figure 1, the potential barrier and reaction heat for the formation of TPR2 are rather comparable with those for the formation of PR2 abstracted by both H and OH radicals. Analogically, the potential barrier and reaction heat for the formation of CTPR2 is quite comparable to those for the formation of CPR2. However, for the formation of CTPR1 and CPR1, the potential barrier for the formation of CTPR1 abstracted by H (3.41 kcal/mol) is much lower than that for the formation of CPR1 abstracted by H (12.43 kcal/mol), while the potential barrier for the formation of CTPR1 abstracted by OH (8.67 kcal/mol) is much higher than that for the formation of CPR1 abstracted by OH (3.20 kcal/mol). This indicates that the sulfydryl-substitution or hydroxyl-substitution of phenyl have no influence on the phenyl-H or phenyl-Cl abstraction, but greatly affect the sulfydryl-H and hydroxyl-H abstraction by H and OH. Moreover, the formation for CTPR1 abstracted by H and OH requires lower potential barriers and are much more exothermic than the formation for TPR2 and CTPR2 abstracted by H and OH, which means that thiophenoxy radical CTPR1 radical is more labile to form and more stable than the sulfydryl-substituted phenyl radicals TPR2 and CTPR2. Similarly, the formation of phenoxy radical CPR1 is energetically preferred to the formation of phenyl radicals PR2 and CPR2.

CTPDR/TPDR can be formed from both CTPR1 and CTPR2/TPR2. Obviously, the potential barriers of CTPDR/TPDR formation from CTPR2/TPR2 abstracted by H and OH radicals are much lower than that those of CTPDR/TPDR formation from CTPR1. In addition, the reactions of CTPDR/TPDR formation from CTPR2/TPR2 release more energy than that those of CTPDR/TPDR formation from CTPR1. For example, the CTPDR formation from CTPR2 abstracted by H has a much lower potential barrier and more exothermic (Δ*E* 3.33 kcal/mol, Δ*H* −21.96 kcal/mol) than that of CTPDR formation from CTPR1 abstracted by H (Δ*E* 9.49 kcal/mol, Δ*H* 2.55 kcal/mol). This implies that CTPDR/TPDR is more likely to form through CTPR2/TPR2 than through CTPR1 abstracted by H and OH radicals. Similarly, CPDR/PDR is more readily produced through CPR2/PR2 than through CPR1. For example, the CPDR formation from CPR2 abstracted by OH has a much lower potential barrier and are more exothermic (Δ*E* 2.01 kcal/mol, Δ*H* −29.02 kcal/mol) than that of CPDR formation from CPR1 abstracted by OH (Δ*E* 3.54 kcal/mol, Δ*H* −3.89 kcal/mol). Comparison of the CTPDR formation from CTPR1 and CTPR2 with the CPDR formation from CPR1 and CPR2 shows that the CTPDR formation can take place by requiring lower potential barriers and releasing more heats than the corresponding CPDR formation reactions, which demonstrates that CTPDR formation reactions are more labile than CPDR formation reactions.

It should be noted in Figure 1 that the potential barriers operating in the formation of C(T)PR1, C(T)PR2, C(T)PDR, (T)PR2, and (T)PDR fall within −2.57 to 43.99 kcal/mol, which could be overcome under high-temperature conditions. The initial radicals C(T)PR1, C(T)PR2, C(T)PDR, (T)PR2, and (T)PDR can be easily formed under the pyrolysis or combustion conditions. The result obtained in this respect suggests that these radical species may be generated in the same temperature condition and there is a great possibility of creating effective collisions between the radicals and molecules.

### 3.2. Formation of Pre-Intermediates of PCTA/PT/DT/DFs via Radical/Molecule Coupling Reactions

#### 3.2.1. Coupling Reaction of C(T)PR1 with 2-C(T)P

As presented in Figure 3, for the O(S)/C couplings, the O/σ-CH coupling is enthalpically more comparable than O/σ-CCl, and S/σ-CH couplings are favored over the S/σ-CCl coupling. For instance, the potential barrier of S/σ-CH and S/σ-CCl couplings of CTPR1 with 2-CTP in Figure 3b is 0.43 and 4.28 kcal/mol, respectively; the reaction heat of S/σ-CH and S/σ-CCl couplings of CTPR1 with 2-CTP in Figure 3b is −0.48 and 10.65 kcal/mol. Furthermore, the ranking of the four carbon–carbon coupling modes is as follows: C_R_H/C_M_H > C_R_Cl/C_M_H > C_R_H/C_M_Cl > C_R_Cl/C_M_Cl. For example, the potential barriers for the formation of IM10 from C_R_H/C_M_H, the formation of IM9 from C_R_Cl/C_M_H, the formation of IM11 from C_R_H/C_M_Cl, and the formation of IM12 from C_R_Cl/C_M_Cl in Figure 3b is 31.88, 35.00, 35.59, and 40.58 kcal/mol, respectively; their reaction heats are 32.28, 35.41, 39.88, and 50.55 kcal/mol, respectively.

In Figure 3a, the potential barriers involved in O/C coupling modes to form ether-thioenoltype intermediates process are within 18.59–21.19 kcal/mol, which are generally lower than those operating in the C/C coupling modes (27.73–35.82 kcal/mol) to form biphenyl intermediates. In addition, the O/C coupling modes (11.45–17.84 kcal/mol) are less endoergic than the C/C coupling modes (25.83–41.17 kcal/mol). Therefore, the O/C coupling modes to form pre-PCPT intermediates are energetically preferable over the C/C coupling modes to form pre-PCDF intermediates in Figure 3a. Similar conclusion could be obtained in the CPR1+2-CP reactions in Figure S1a of Supplementary Materials [41]. Analogously, the S/C coupling modes to produce pre-PCTA/PT intermediates are energetically preferred to the C/C coupling modes in Figure 3b,c to form pre-PCDT intermediates. In other words, the radical/molecule coupling of C(T)PR1 with 2-C(T)P is difficult to occur in the C/C coupling. The combination reactions of C(T)PR1 with 2-C(T)P tend to take place via the phenoxy oxygen or thiophenoxy sulfur sites and to produce (thio)ether-(thio)enol type intermediates analogues, which proceed via elimination of H, (thio)phenolic H abstraction, ring close, and Cl elimination steps and eventually lead to the formation of PCTA/PTs [41]. To sum up, the ranking of the S/C or O/C coupling to form pre-PCTA/PT/DDs intermediates is as follows: CTPR1 + 2-CTP > CTPR1 + 2-CP >> CPR1 + 2-CP ≈ CPR1 + 2-CTP.

In particular, the S/C couplings require overcoming dramatically lower potential barriers (0.43–4.28 kcal/mol in Figure 3b and 1.25–4.52 kcal/mol in Figure 3c) than O/C coupling (18.59–21.19 kcal/mol in Figure 3a and 19.53–20.87 kcal/mol in Figure S1a) by approximate 20 kcal/mol. In addition, the S/C coupling are less endoergic (−0.48–10.65 kcal/mol in Figure 3b and 0.51–9.53 kcal/mol in Figure 3c) than the O/C coupling (11.45–17.84 kcal/mol in Figure 3a and 13.18–19.10 kcal/mol in Figure S1a). Thus, the S/C coupling modes are overwhelmingly superior to the O/C coupling, which indicates that the formation of thioether-(thio)enol type intermediates is much easier than that of ether-(thio)enol type intermediates. In other words, the thiophenolic sulfur centered radical/molecule coupling is more labile to happen than the phenolic oxygen centered radical/molecule coupling. Based on these results, we could deduce that although the radical/molecule coupling is uncompetitive compared with the radical/radical coupling for PCDD formation from CPs as stated in the literature [41], the radical/molecule coupling can play an important role in the PCTA/PT formation from CTPs and CPs. Specifically, the radical/molecule coupling formation of PCPTs can be only achieved via the route of sulfur-centerd thiophenoxy radical attack to 2-CP, but not the route of oxygen-centered phenoxy radical attack to 2-CTP.

The coupling reaction of CTPR1 with 2-CTP were also studied by Dar et al. at the B3LYP/6-311+G(d,p) level [23,24]. Two obvious differences were observed: (1) The S/σ-CCl couplings of CTPR1 with 2-CTP requires crossing a potential barrier of 15.6 kcal/mol in the study of Dar et al. [23,24], whereas the potential barrier is 4.28 kcal/mol in our study; (2) another coupling mode reported by Dar et al. is different from the S/σ-CH coupling mode in our study. The coupling mode provided by Dar et al. was accompanied by the elimination of HCl with a significantly higher potential barrier 26.4 kcal/mol, whereas the S/σ-CH couplings occurred with no atom loss and with a trivial potential barrier (0.43 kcal/mol). The S/σ-CH couplings reported in our study are energetically more favorable than the coupling mode reported by Dar et al. [23,24]. The discrepancy for S/σ-CCl couplings may arise from the different calculation levels. The energies of Dar et al. are calculated at the B3LYP level [23,24], while our energy calculations are carried out at the MPWB1K level. It is well known that the B3LYP method does not consider the electronic correlation and systematically overestimates or underestimates barrier heights. In order to further compare with the S/σ-CCl coupling mode proposed by Dar et al. [23,24], we checked the transition state structures of S/σ-CCl coupling. Figure S2 in Supplementary Materials shows configurations of transition states from the S/C coupling of CTPR1 with 2-CTP located by Dar et al. and us [24]. The -SH in TS-6 reported by Dar et al. point to the direction far away from Cl atom, whereas the -SH in TS36 in our study point to Cl atom and form an intramolecular H bond, which could stabilise the structure and reduce the potential barrier [24]. Thus, the energy value of S/σ-CCl coupling in our study may be more accurate than that from Dar et al. The potential barriers of S/C coupling in our study (0.43 and 4.28 kcal/mol) are significantly lower than those from Dar et al. (26.4 and 15.6 kcal/mol). The almost barrier-less energy value in our study indicate that the radical/molecular coupling of CTPR with CP are nearly comparable with the corresponding steps involved in the radical/radical reactions of CTPRs [26], and can contribute to the PCTA formation, which has been ignored by Dar et al. [23,24].

#### 3.2.2. Coupling Reactions of C(T)PR2 and (T)PR2 with 2-C(T)P

The reactions of C(T)PR2 with 2-C(T)P or (T)PR2 with 2-C(T)P can only occur in the C/C coupling, resulting in the formation of biphenyl intermediates and transference into PCDT/DFs. It is evident from Figure 4 that the C•/CH coupling can take place much easier than the C•/CCl coupling. For example, all C•/CH couplings shown in Figure 4 can occur encountering potential barriers ranging from 1.93 to 4.14 kcal/mol, which are much lower than those of C•/CCl coupling (8.25–12.26 kcal/mol). In addition, the C•/CH coupling are estimated to release heats from −27.63 to −24.94 kcal/mol, which are much more exothermic than the C•/CCl coupling at −19.72 to −16.79 kcal/mol. It is also interesting to compare the formation potential of four kinds of C(T)PR2 with 2-C(T)P couplings and four kinds of (T)PR2 with 2-C(T)P couplings. According to Figure 4 and Figure S1b,d, the formation potential ranking of C(T)PR2 with 2-C(T)P couplings is CPR2 + 2-CTP > CPR2 + 2-CP > CTPR2 + 2-CP > CTPR2 + 2-CTP; the formation potential ranking of (T)PR2 with 2-C(T)P couplings is PR2 + 2-CP > PR2 + 2-CTP > TPR2 + 2-CP > TPR2 + 2-CTP. All the potential barriers of C•/CH coupling in Figure 4 are so trivial that they could be easily be overcome in high-temperature conditions, which means that the radical/molecule coupling of C(T)PR2 and (T)PR2 with 2-C(T)P can compete with the radical/radical combination reactions. Additionally, the similar thermodynamic values for the pre-PCDT formation from CTPR2/TPR2 with 2-CTP and pre-PCDF formation from CPR2/PR2 with 2-CP in Figure S1 imply the oxygen or sulfur substitution have a consistent effect on the PCDT/DF formation. All the results support the fact that the sulfydryl/hydroxyl-substituted phenyl radicals can initialise the feasible reactions to form pre-PCDT/DF intermediates, followed by the elimination of H, phenolic H abstraction, and ring close, and the elimination of OH/SH, to produce PCDT/DFs.

#### 3.2.3. Coupling Reactions of C(T)PDR and (T)PDR with 2-C(T)P

Similar to the O(S)/C couplings from C(T)PR1 with 2-C(T)P in Figure 3, the O(S)/σ-CH couplings are enthalpically preferred to O(S)/σ-CCl. For example, in Figure 5b, the S/σ-CH coupling from CTPDR + 2-CTP (Δ*E* 7.50 kcal/mol, Δ*H* 7.21 kcal/mol) can occur at a much lower potential barrier and is less endothermic than S/σ-CCl coupling (Δ*E* 12.16 kcal/mol, Δ*H* 17.91 kcal/mol), which means the S/σ-CH coupling is more feasible than S/σ-CCl coupling. Furthermore, analogous to the C•/CH coupling from C(T)PR2 and (T)PR2 with 2-C(T)P in Figure 4, the C•/CH coupling can occur much easier than the C•/CCl coupling from the C(T)PDR and (T)PDR with 2-C(T)P in Figure 5. For example, in Figure 5b, the C•/CH coupling from CTPDR + 2-CTP (Δ*E* 2.27 kcal/mol, Δ*H* −24.80 kcal/mol) requires a much lower potential barrier and is more exothermic than C•/CCl coupling (Δ*E* 11.78 kcal/mol, Δ*H* −9.30 kcal/mol). This implies the IM34 from the C•/CH coupling is much easier to form than IM35 from the C•/CCl coupling. To sum up, in Figure 5, the formation of IM30, IM32, IM34, IM36, IM38, IM40, IM42, IM44, IM46, IM48, IM50, and IM52 are much easier to form than IM31, IM32, IM35, IM37, IM39, IM41, IM43, IM45, IM47, IM49, IM51, and IM53, respectively. O(S)/σ-CH couplings can be followed by the elimination of H, ring closure, and elimination of OH/SH to form PCDT/DFs, while the C•/CH coupling can be followed by the abstraction of H, ring closure, and intramolecular elimination of SH/OH elimination to form PCDT/DFs.

As illustrated in Figure 5, in terms of the enthalpically preferred C•/CH and O(S)/σ-CH couplings from C(T)PDR and (T)PDR with 2-C(T)P, the C•/CH coupling modes to form pre-PCDT/DF intermediates are more comparable with the O(S)/σ-CH couplings modes to form pre-PCDT/DF intermediates. For instance, in Figure 5a,d, the potential barriers involved in C•/CH modes to form keto-thioenol intermediates (0.61–1.29 kcal/mol) are generally lower than those involved in the corresponding O/σ-CH coupling modes (19.09–19.95 kcal/mol) to form ether-thioenoltype intermediates. In addition, the C•/CH coupling modes are strongly exothermic, ranging from −27.41 to −27.36 kcal/mol, while the O/σ-CH coupling are endothermic amounting to 9.71–10.30 kcal/mol in Figure 5a,d. This is consistent with the values of CPDR + 2-CP and PDR + 2-CP couplings in Figure S1c,e of Supplementary Materials but totally in contrary to the conclusion that the O/C coupling are favored over the C/C coupling for CPR1 with 2-C(T)P in Figure 3, and Figure S1a that the O/C coupling are favored over the C/C coupling. To sum up, the most feasible pathway in each condensation in Figure 5a–f is the C•/CH coupling to form pre-PCDT/DF intermediates. In other words, IM30, IM34, IM38, IM42, IM46, and IM50 are the main intermediates from reactions of C(T)PDR and (T)PDR with 2-C(T)P. Obviously, according to Figure 5 and Figure S1c,e, the formation potential ranking of most the feasible routes from C(T)PDR with 2-C(T)P coupling is CPDR + 2-CTP > CPDR + 2-CP > CTPDR + 2-CTP > CTPDR + 2-CP; the formation potential ranking of the most feasible routes from(T)PDR with 2-C(T)P couplings is PDR + 2-CTP > PDR + 2-CP > TPDR + 2-CTP > TPDR + 2-CP.

In Figure 5, a much lower potential barrier is involved in the S/C couplings than that required in the O/C condensations by about 10 kcal/mol, indicating that the S/C coupling pathways are more comparable than the O/C pathways. This agrees with the conclusion above that the S/C coupling modes are overwhelmingly superior to the O/C coupling modes. In contrary, the oxygen or sulfur substitution have little effect on the C•/CH or C•/CCl coupling, as the potential barriers and reaction heats of these coupling are similar, which is consistent with the coupling reactions of C(T)PR2 and (T)PR2 with 2-C(T)P.

#### 3.2.4. Comparing the Reactions of Thiophenoxy Radicals with 2-C(T)P Couplings, Sulfydryl/Hydroxyl-Substituted Phenyl Radicals with 2-C(T)P Couplings, and (thio)phenoxyl Diradicals with 2-C(T)P Couplings

To sum up, the thiophenoxy radicals with 2-C(T)P couplings in Figure 3 mainly produce the pre-PCTA/PT intermediates, with the potential barriers involved falling within 0.43 to 4.52 kcal/mol and reaction heats ranging from −0.48 to 10.65 kcal/mol. The sulfydryl/hydroxyl-substituted phenyl radicals with 2-C(T)P couplings in Figure 4 and the (thio)phenoxyl diradicals with 2-C(T)P couplings in Figure 5 can only generate PCDT/DF products. The potential barriers of the energetically favorable C•/CH coupling in Figure 4 range 1.93–4.14 kcal/mol, accompanied by reaction heats varying from −27.63 to −24.94 kcal/mol. The potential barriers of the energetically more comparable to C•/CH coupling in Figure 5 are amounted to 0.61–2.84 kcal/mol, and are simultaneously associated with strong exothermicities in the range of −27.41 to −24.16 kcal/mol. Thus, the radical/molecule coupling of the three kinds radicals with 2-C(T)P demands virtually the same potential barriers. However, the pre-PCDT/DF intermediates formation from the sulfydryl/hydroxyl-substituted phenyl radicals with 2-C(T)P couplings and (thio)phenoxyl diradicals with 2-C(T)P couplings are much more exothermic than the pre-PCTA/PT intermediates formation from thiophenoxy radicals with 2-C(T)P couplings. It is evident that the pre-PCDT/DF intermediates are much easier to form and more stable than the pre-PCTA/PT intermediates. Thus, the PCDT/DFs are much more liable to form than PCTA/PTs from the radical/molecule coupling from 2-C(T)P as precursors. In particular, for the formation of PCTA/DTs from the three radical/molecule couplings from 2-CTP, the pre-PCDT intermediates formation pathways (Δ*E* 2.27–4.14 kcal/mol, Δ*H* −25.88–−24.80 kcal/mol) in Figure 4b,e and Figure 5b,e are overwhelmingly superior to the pre-PCTA intermediates formation pathways (Δ*E* 0.43–4.28 kcal/mol, Δ*H* −0.48–10.65 kcal/mol) in Figure 3b. This provides reasonable explanation for the high PCDT-to-PCTA ratio under the pyrolysis or combustion conditions [16,17].

### 3.3. Rate Constant Calculations

The thermodynamic analysis of formation from reactions of C(T)PR1 with 2-C(T)P and C(T)PDR and (T)PDR with 2-C(T)P shows that S/C coupling are preferred over O/C coupling. Comparing the average calculated TST rate constants in these two couplings also proved this conclusion. For example, in Table 3, at 1000 K, the average TST rate constant for S/C coupling from reactions of C(T)PR1 with 2-C(T)P is 2.60 × 10^−16^ cm^3^ molecule^−1^ s^−1^, which is larger than the value 1.75 × 10^−20^ cm^3^ molecule^−1^ s^−1^ for O/C coupling from reactions of C(T)PR1 with 2-C(T)P. Analogously, the average TST rate constant for S/C coupling from reactions of C(T)PDR and (T)PDR with 2-C(T)P (1.59 × 10^−17^ cm^3^ molecule^−1^ s^−1^) is larger than that of O/C coupling from reactions of C(T)PDR and (T)PDR with 2-C(T)P (3.24 × 10^−20^ cm^3^ molecule^−1^ s^−1^).

As presented in Table 1, Table 2 and Table S2 of Supplementary Materials, the TST rate constants for C•/CH couplings are larger than those of C•/CCl couplings from coupling reactions of C(T)PR2 and (T)PR2 with 2-C(T)P and coupling reactions of C(T)PDR and (T)PDR with 2-C(T)P over the whole studied temperature range. For instance, at 1000 K, the TST rate constants for the formation of IM19, IM21, IM23, IM25, IM27, and IM29 from C•/CH coupling are 6.32 × 10^−16^, 1.33 × 10^−16^, 5.54 × 10^−16^, 3.11 × 10^−16^, 1.84 × 10^−16^, and 9.70 × 10^−16^, while the values are 7.01 × 10^−18^, 6.31 × 10^−19^, 7.82 × 10^−18^, 3.72 × 10^−18^, 3.95 × 10^−19^, and 1.02 × 10^−17^ for the formation of IM20, IM22, IM24, IM26, IM28, IM26 from C•/CCl coupling in Table 1 and Table S2 of Supplementary Materials. This is in good agreement with the comparison of activation Gibbs free energies that the ∆G^≠^ of C•/CH coupling (−14.30 to 16.99 kcal/mol) are all less than that of C•/CCl coupling (21.74–26.06 kcal/mol) in Table S3. In addition, at 800 K, the TST rate constants for the C•/CH coupling reaction of CTPDR + 2-CTP → IM34 via TS63 (1.73 × 10^−16^ cm^3^ molecule^−1^ s^−1^) is larger than that of for the C•/CCl reaction of CTPDR + 2-CTP → IM35 via TS64 (1.15 × 10^−19^ cm^3^ molecule^−1^ s^−1^) in Table 2 and Table S2 of Supplementary Materials. Similarly, the activation entropies of CTPDR + 2-CTP → IM34 via TS63 (−43.82 cal mol^−1^ K^−1^) is higher than that of CTPDR + 2-CTP → IM35 via TS64 (−46.74 cal mol^−1^ K^−1^) in Table S3. This perfectly matches the thermodynamic analysis above that C•/CH coupling are kinetically more efficient than the C•/CCl coupling.

Similarly, the kinetic data also can confirm the thermodynamic analysis from reactions of C(T)PR1 with 2-C(T)P and C(T)PDR and (T)PDR with 2-C(T)P that the S/σ-CH coupling is more likely to occur than the S/σ-CCl coupling over the whole studied temperature range. For instance, at 800 K, the TST rate constants for the S/σ-CH coupling reaction of CTPR1 + 2-CTP → IM7 via TS35 is 1.34 × 10^−16^ cm^3^ molecule^−1^ s^−1^, which is larger than the calculated value for the S/σ-CCl reaction of CTPR1 + 2-CTP → IM8 via TS36 is 7.14 × 10^−18^ cm^3^ molecule^−1^ s^−1^ in Table 1 and Table S2 of Supplementary Materials. In addition, at 1000 K, the TST rate constants for the S/σ-CH coupling reaction of CTPDR + 2-CTP → IM36 via TS65 (1.49 × 10^−17^ cm^3^ molecule^−1^ s^−1^) is larger than that of the S/σ-CCl reaction of CTPDR + 2-CTP → IM37 via TS66 (4.20 × 10^−19^ cm^3^ molecule^−1^ s^−1^) in Table 2 and Table S2 of Supplementary Materials. The activation Gibbs free energy of CTPDR + 2-CTP → IM36 via TS65 (20.87 kcal/mol) is smaller than that CTPDR + 2-CTP → IM37 via TS66 (26.09 kcal/mol) in Table S3. This is consistent with the energy analysis above that the S/σ-CH couplings are energetically more favorable than the S/σ-CCl couplings.

## 4. Materials and Methods

### 4.1. Density Functional Theory

All the quantum chemical calculations on the structure, frequency, and energy of related substances such as reactants, products, intermediates, and transition states were performed by using the Gaussian 09 program by using the MPWB1K method [56]. The MPWB1K method is one of the most efficient and high-precision configuration optimization and frequency calculation methods relative to computational cost [57], and has been successfully performed for formation of PCDT/TAs from 2,4-DCTP as precursor [26], formation of polyhalogenated dibenzo-p-dioxins from hydroxylated polybrominated diphenyl ethers [58], atmospheric degradation of 2,3,7,8-TCDD [59,60], and 2,4-dibrominated diphenyl ether [61]. Geometries were optimized at the MPWB1K/6-31+G(d,p) level of theory. The obtained structures were confirmed as stable configuration or transition state by using corresponding frequency calculation at the same level. The intrinsic reaction coordinate (IRC) was calculated at the MPWB1K/6-31+G(d,p) level to verify that the transition state connects to the right minima along the reaction path [62]. In order to get more precise energy values, a more flexible basis set, 6-311+G(3df,2p), was employed to determine the single point energies, including zero-point energy correction (ZPE).

### 4.2. Kinetic Calculation

The kinetic and statistical thermodynamic (KiSThelP) program, a cross-platform free open-source program developed to estimate molecule and reaction properties from electronic structure data, was used to calculate the reaction rate constants [49]. KiSThelP offers a range of features that can be helpful for users more experienced in computational kinetics. The conventional transition state theory (TST) method was applied to calculate the rate constants for all the radical/molecule combination reactions in the typical temperature of incinerator (from 600–1200 K). The effect of quantum tunneling on rate constants was considered based on the one-dimensional Wigner’s formalism as implemented in the KiSThelP program [50].

### 4.3. Accuracy Verification

It is critical to prove the accuracy of the theoretical calculations, and we calculated optimized geometries and vibrational frequencies of 4-chlorothiophenol, thiophenol, phenol, and dibenzothiophene at the MPWB1K/6-31+G(d,p) level which match well with the available experimental values, and the relative error remains within 1% for the geometry parameters and 9% for the vibrational frequencies [63,64,65]. To check the reliability of the energies, we calculated the reaction enthalpies for the reactions of thiophenol (C_6_H_6_S) + thiophenol (C_6_H_6_S) → dibenzothiophene (C_12_H_8_S) + H_2_S + H_2_, phenol (C_6_H_6_O) + phenol (C_6_H_6_O) → dibenzofuran (C_12_H_8_O) + H_2_O + H_2_, and phenol (C_6_H_6_O) + phenol (C_6_H_6_O) → dibenzo-p-dioxin (C_12_H_8_O_2_) + 2H_2_ at the MPWB1K/6-311+G(3df,2p)//MPWB1K/6-31+G(d,p) level. The calculated values of −7.60, 0.56, and 31.28 kcal/mol at 298.15 K and 1.0 atm are in good agreement with the corresponding experimental values of −7.74, −0.43, and 31.91 kcal/mol, derived from the measured standard enthalpies of formation ∆_f_H (298.15 K) of thiophenol (26.85 kcal/mol), phenol (−96.36 ± 0.59 kcal/mol), dibenzothiophene (50.88 kcal/mol), dibenzofuran (47.3 ± 4.8 kcal/mol), dibenzo-p-dioxin (59.2 ± 4.4 kcal/mol), H_2_S (−4.92 kcal/mol), H_2_O (−241.83 kcal/mol), and H_2_ (0 kcal/mol) [26,66], particularly if the experimental uncertainties are taken into account. So the MPWB1K/6-311+G(3df,2p)//MPWB1K/6-31+G(d,p) level used in this study can meet the calculation accuracy for the species involved in the formation of PCTA/PT/DT/DFs from 2-chlorothiophenol and 2-chlorophenol precursors.

## 5. Conclusions

In this study, the radical/molecule initial formation pathways of PCTA/PT/DT/DFs from the cross-condensation reactions of chloro(thio)phenoxy radical, sulfydryl/hydroxyl-substituted phenyl radical, and (thio)phenoxyl diradical with 2-C(T)P were investigated theoretically using DFT electronic structure theory at the MPWB1K/6-31+G(d,p)//MPWB1K/6-31+G(d,p) level. The kinetic calculation was performed and the rate constants were calculated over the temperature range of 600–1200 K using the conventional transition state theory (TST) method, which can generate accurate input parameters for the dioxin formation models. The values were compared with the previous studies on the radical/radical formation mechanism of PCTA/DFs from CTPRs and the radical/molecule formation mechanism of PCDFs from oxygen-substituted radicals with CP [26,41]. Our study found that the radical/molecule mechanism can contribute to the gas-phase formation of PCTA/PT/DT/DFs under the pyrolysis or combustion conditions, which has been ignored before previous work [26] indicated that the radical/molecule coupling of chlorophenoxy radical, hydroxyl-substituted phenyl radical, and phenoxyl diradical with 2-CP can only form pre-PCDFs intermediates, and the O/C coupling to form pre-PCDDs intermediates was not important. However, we found in this study that the radical/molecule coupling of sulfur-substituted chlorothiophenoxy radical, sulfydryl-substituted phenyl radical, and thiophenoxyl diradical with 2-CTP could contribute to both the formation of pre-PCDTs and pre-PCTAs intermediates. Four conclusions can be summarized, as follows:

(1) The S/C coupling modes are preferable over the O/C coupling modes. The S/C coupling modes can form pre-PCTAs and pre-PCPTs intermediates in the coupling of chloro(thio)phenoxy radical with 2-C(T)P and form pre-PCDTs intermediates in the coupling of (thio)phenoxyl diradical with 2-C(T)P.

(2) For the self-coupling of 2-CTP and corresponding sulfur-substituted radicals, the pre-PCTA intermediates can only be produced from the coupling of chlorothiophenoxy radical with 2-CTP, and the pre-PCDT intermediates can be formed from the coupling of sulfydryl-substituted phenyl radical with 2-CTP and the coupling of thiophenoxyl diradical with 2-CTP. The pre-PCDT intermediates formation pathways are more favorable than the pre-PCTA intermediates formation pathways, which, to some extent, can give reasonable explanation for the high PCDT-to-PCTA ratio under the pyrolysis or combustion conditions.

(3) The S(O)/σ-CH couplings are energetically more favorable than the S(O)/σ-CCl couplings to form the (thio)ether-(thio)enol intermediates, and the C•/CH coupling can take place much easier than the C•/CCl coupling to form the (thio)keto-(thio)enol intermediates.

(4) In the case of chloro(thio)phenoxy radical with 2-C(T)P, the S/C coupling to form pre-PCTA/PTs intermediates are more likely to occur than the C/C coupling to form pre-PCDT/DFs intermediates. However, in the case of (thio)phenoxyl diradical with 2-C(T)P, the C/C coupling are much easier than the S/C coupling, both of which can contribute to the formation of pre-PCDT/DFs intermediates.

## Figures and Tables

**Table 1:** Arrhenius formulas for pre-PCTA/PT/DT/DF formation routes from the cross-condensation reactions of 2-C(T)P with C(T)PR1, C(T)PR2, and (T)PR2, over the temperature range of 600–1200 K (unit is cm^3^ molecule^−1^ s^−1^).

Reactions Arrhenius Formulas	Arrhenius Formulas
CPR1 + 2-CTP → IM1 via TS29	*k*(T) = (2.09 × 10^−15^) exp (−11163/T)
CPR1 + 2-CTP → IM2 + Cl via TS30	*k*(T) = (1.79 × 10^−15^) exp (−12526/T)
CPR1 + 2-CTP → IM3 via TS31	*k*(T) = (1.43 × 10^−15^) exp (−17118/T)
CPR1 + 2-CTP → IM4 + Cl via TS32	*k*(T) = (3.90 × 10^−15^) exp (−15840/T)
CPR1 + 2-CTP → IM5 via TS33	*k*(T) = (1.42 × 10^−15^) exp (−17613/T)
CPR1 + 2-CTP → IM6 + Cl TS34	*k*(T) = (1.21 × 10^−16^) exp (−19231/T)
CTPR1 + 2-CTP → IM7 via TS35	*k*(T) = (2.37 × 10^−15^) exp (−2233/T)
CTPR1 + 2-CTP → IM8 via TS36	*k*(T) = (1.22 × 10^−15^) exp (−4093/T)
CTPR1 + 2-CTP → IM9 via TS37	*k*(T) = (1.40 × 10^−15^) exp (−19723/T)
CTPR1 + 2-CTP → IM10 via TS38	*k*(T) = (5.33 × 10^−15^) exp (−18080/T)
CTPR1 + 2-CTP → IM11 via TS39	*k*(T) = (1.99 × 10^−15^) exp (−19993/T)
CTPR1 + 2-CTP → IM12 + Cl TS40	*k*(T) = (5.24 × 10^−16^) exp (−22527/T)
CTPR1 + 2-CP → IM13 via TS41	*k*(T) = (1.14 × 10^−14^) exp (−2790/T)
CTPR1 + 2-CP → IM14 + Cl via TS42	*k*(T) = (1.01 × 10^−14^) exp (−4524/T)
CTPR1 + 2-CP → IM15 via TS43	*k*(T) = (6.13 × 10^−15^) exp (−19327/T)
CTPR1 + 2-CP → IM16 + Cl via TS44	*k*(T) = (1.06 × 10^−14^) exp (−17908/T)
CTPR1 + 2-CP → IM17 via TS45	*k*(T) = (4.46 × 10^−15^) exp (−19296/T)
CTPR1 + 2-CP → IM18 + Cl TS46	*k*(T) = (1.29 × 10^−15^) exp (−21760/T)
CPR2 + 2-CTP → IM19 via TS47	*k*(T) = (1.42 × 10^−14^) exp (−3082/T)
CPR2 + 2-CTP → IM20 + Cl via TS48	*k*(T) = (3.37 × 10^−15^) exp (−6141/T)
CTPR2 + 2-CTP → IM21 via TS49	*k*(T) = (7.46 × 10^−15^) exp (−3994/T)
CTPR2 + 2-CTP → IM22 + Cl via TS50	*k*(T) = (1.20 × 10^−15^) exp (−7520/T)
CTPR2 + 2-CP → IM23 via TS51	*k*(T) = (3.19 × 10^−14^) exp (−4022/T)
CTPR2 + 2-CP → IM24 + Cl TS52	*k*(T) = (5.48 × 10^−15^) exp (−6518/T)
PR2 + 2-CTP → IM25 via TS53	*k*(T) = (1.26 × 10^−14^) exp (−3668/T)
PR2 + 2-CTP → IM26 + Cl via TS54	*k*(T) = (1.53 × 10^−15^) exp (−5987/T)
TPR2 + 2-CTP → IM27 via TS55	*k*(T) = (1.21 × 10^−14^) exp (−4148/T)
TPR2 + 2-CTP → IM28 + Cl via TS56	*k*(T) = (1.21 × 10^−15^) exp (−7993/T)
TPR2 + 2-CP → IM29 via TS57	*k*(T) = (4.85 × 10^−14^) exp (−3879/T)
TPR2 + 2-CP → IM26 + Cl via TS58	*k*(T) = (9.06 × 10^−15^) exp (−6757/T)

**Table 2:** Arrhenius formulas for pre-PCDT/DF formation routes from the cross-condensation reactions of 2-C(T)P with C(T)PDR and (T)PDR over the temperature range of 600–1200 K (unit is cm^3^ molecule^−1^ s^−1^).

Reactions Arrhenius Formulas	Arrhenius Formulas
CPDR + 2-CTP → IM30 via TS59	*k*(T) = (3.33 × 10^−14^) exp (−2430/T)
CPDR + 2-CTP → IM31 + Cl via TS60	*k*(T) = (8.91 × 10^−15^) exp (−5222/T)
CPDR + 2-CTP → IM32 via TS61	*k*(T) = (2.36 × 10^−15^) exp (−11424/T)
CPDR + 2-CTP → IM33 + Cl via TS62	*k*(T) = (5.26 × 10^−15^) exp (−12811/T)
CTPDR + 2-CTP → IM34 via TS63	*k*(T) = (1.05 × 10^−14^) exp (−3225/T)
CTPDR + 2-CTP → IM35 + Cl via TS64	*k*(T) = (2.45 × 10^−15^) exp (−7902/T)
CTPDR + 2-CTP → IM36 via TS65	*k*(T) = (5.65 × 10^−15^) exp (−5904/T)
CTPDR + 2-CTP → IM37 + Cl via TS66	*k*(T) = (2.03 × 10^−15^) exp (−8446/T)
CTPDR + 2-CP → IM38 via TS67	*k*(T) = (1.43 × 10^−13^) exp (−3672/T)
CTPDR + 2-CP → IM39 + Cl via TS68	*k*(T) = (1.72 × 10^−14^) exp (−6042/T)
CTPDR + 2-CP → IM40 via TS69	*k*(T) = (1.31 × 10^−14^) exp (−6256/T)
CTPDR + 2-CP → IM41 + Cl TS70	*k*(T) = (1.04 × 10^−14^) exp (−8632/T)
PDR + 2-CTP → IM42 via TS71	*k*(T) = (3.51 × 10^−14^) exp (−2775/T)
PDR + 2-CTP → IM43 + Cl via TS72	*k*(T) = (4.24 × 10^−15^) exp (−5424/T)
PDR + 2-CTP → IM44 via TS73	*k*(T) = (6.46 × 10^−15^) exp (−11909/T)
PDR + 2-CTP → IM45 + Cl TS74	*k*(T) = (2.54 × 10^−14^) exp (−13132/T)
TPDR + 2-CTP → IM46 via TS75	*k*(T) = (1.37 × 10^−14^) exp (−3265/T)
TPDR + 2-CTP → IM47 + Cl via TS76	*k*(T) = (1.88 × 10^−15^) exp (−7484/T)
TPDR + 2-CTP → IM48 via TS77	*k*(T) = (4.21 × 10^−14^) exp (−6501/T)
TPDR + 2-CTP → IM49 + Cl TS78	*k*(T) = (7.95 × 10^−15^) exp (−8890/T)
TPDR + 2-CP → IM50 via TS79	*k*(T) = (8.93 × 10^−14^) exp (−3646/T)
TPDR + 2-CP → IM51 + Cl via TS80	*k*(T) = (9.45 × 10^−15^) exp (−5768/T)
TPDR + 2-CP → IM52 via TS81	*k*(T) = (2.02 × 10^−14^) exp (−6885/T)
TPDR + 2-CP → IM53 + Cl TS82	*k*(T) = (3.08 × 10^−14^) exp (−9091/T)

**Table 3:** Average rate constants calculated at 1000 K for pre-PCTA/PT/DT/DF formation routes from the cross-condensation reactions between C(T)PR1 with 2-C(T)P, between C(T)PR2 and (T)PR2 with 2-C(T)P, and between C(T)PDR and (T)PDR with 2-C(T)P (unit is cm^3^ molecule^−1^ s^−1^).

C(T)PR1 with 2-C(T)P			C(T)PR2 and (T)PR2 with 2-C(T)P	C(T)PDR and (T)PDR with 2-C(T)P		
C/C type	O/C type	S/C type	C/C type	C/C type	O/C type	S/C type
7.28 × 10^−23^	1.75 × 10^−20^	2.60 × 10^−16^	2.34 × 10^−16^	9.82 × 10^−16^	3.24 × 10^−20^	1.59 × 10^−17^

## Supplementary Materials

Supplementary materials can be found at https://www.mdpi.com/1422-0067/20/7/1542/s1. Figure S1. Pre-PCDD/DF formation pathways embedded with the potential barriers Δ*E* (in kcal/mol) and reaction heats Δ*H* (in kcal/mol) from the coupling reactions of CPR1 with 2-CP (a), CPR2 with 2-CP (b) and CPDR with 2-CP (c), PR2 with 2-CP (d) and PDR with 2-CP (e). Δ*H* is calculated at 0 K. Figure S2. The optimized geometries for transition states for pre-PCTA formation routes from the S/C coupling of cross-condensation reactions of CTPR1 with 2-CTP. The red dotted line represents intramolecular hydrogen bonds (^a^Reference [24], previously calculated by Dar et al. at B3LYP/6-311+G(d,p) level of theory) Figure S3. MPWB1K/6-31+G(d,p) optimized geometries for transition states of the coupling reactions of CTPR1 with 2-CTP, CTPR2 with 2-CTP, CTPDR with 2-CTP, CPR1 with 2-CP and PR2 with 2-CP. Distances are in Å. Table S1. Imaginary frequencies (in cm^−1^), zero point energies (ZPE, in a.u.) and total energies (without ZPE, in a.u.) for transition states involved in the formation of pre-PCTA/PT/DT/DF intermediates. Table S2. TST rate constants for for pre-PCTA/PT/DT/DF formation routes from the cross-condensation reactions of 2-C(T)P with C(T)PR1, C(T)PR2 and (T)PR2, and C(T)PDR and (T)PDR over the temperature range of 600–1200 K (units are cm^3^ molecule^−1^ s^−1^). Table S3. Activation enthalpies (∆*H*^≠^), activation Gibbs free energies (∆*G*^≠^), activation entropies (∆*S*^≠^), relative enthalpies (∆*_r_H*), relative Gibbs free energies (∆*_r_G*), relative entropies (∆*_r_S*) calculated at 298.15 K and 1 atm. (unit is kcal /mol for ∆*G*^≠^, ∆*H*^≠^, ∆*_r_G* and ∆*_r_H*, unit is cal mol^−1^ K^−1^ for ∆*S*^≠^ and ∆*_r_S*). Table S4. Cartesian coordinates for transition states involved in the formation routes of pre-intermediates for PCTA/PT/DT/DFs, x coordinate, y coordinate and z coordinate. Table S5. Cartesian coordinates for reactions, intermediate and products involved in the formation routes of pre-intermediates for PCTA/PT/DT/DFs, x coordinate, y coordinate and z coordinate.

## Abbreviations

PCDTs	polychlorinated dibenzothiophenes
PCPTs	polychlorinated phenoxathiins
PCTAs	polychlorinated thianthrenes
PCDDs	polychlorinated dibenzo-p-dioxins
PCDFs	polychlorinated dibenzofurans
2-CP	2-chlorophenol
2-CTP	2-chlorothiophenol
CPR1	2-chlorophenoxy
CPR2	2-hydroxyl-3-chloro-phenyl
CPDR	chlorinated phenoxyl diradical
PR2	2-hydroxylphenyl radical
PDR	phenoxyl diradical
CTPR1	2-chlorothiophenoxy
CTPR2	2-sulfydryl-3-chloro-phenyl
CTPDR	chlorinated thiophenoxyl diradical
TPR2	2-sulfydrylphenyl radical
TPDR	thiophenoxyl diradical
TST	transition state theory
IRC	intrinsic reaction coordinate
KiSThelP	kinetic and statistical thermodynamic
ZPE	zero-point energy
