# Supplementary material for: Quantum Chemical and Kinetic Study on Radical/Molecule Formation Mechanism of Pre-Intermediates for PCTA/PT/DT/DFs from 2-Chlorothiophenol and 2-Chlorophenol Precursors
Source: Int J Mol Sci. 2019 Mar 27;20(7):1542. doi: 10.3390/ijms20071542 (PMC6480007; doi:10.3390/ijms20071542)
Supplement: Supplementary file 1 [file ijms-20-01542-s001.pdf]

Supplementary Materials for

Quantum chemical and kinetic study on radical/molecule formation mechanism of pre-intermediates for PCTA/PT/DT/DFs from 2-chlorothiophenol and 2-chlorophenol precursors

Chenpeng Zuo[1], Hetong Wang[1], Wenxiao Pan[2], Siyuan Zheng[1], Fei Xu[1,3] *, Qingzhu Zhang[1]

[1] Environment Research Institute, Shandong University, Qingdao 266237, P. R. China; zuochenpeng@126.com (C.Z.), Kishi_Wang@163.com (H.W.), zhengsiyuan1991@126.com (S.Z.), zqz@sdu.edu.cn (Q.Z.)

[2] State Key Laboratory of Environmental Chemistry and Ecotoxicology, Research Center for Eco-Environmental Sciences, Chinese Academy of Sciences, Beijing 100085, P. R. China; wxpan@rcees.ac.cn (W.P.)

[3] Shenzhen Research Institute, Shandong University, Shenzhen 518057, P. R. China

* Correspondence: xufei@sdu.edu.cn (F.X.); Tel.: +86-532-58631992

Contains three figures and five tables

Figure S1. Pre-PCDD/DF formation pathways embedded with the potential barriers Δ*E* (in kcal/mol) and reaction heats Δ*H* (in kcal/mol) from the coupling reactions of CPR1 with 2-CP (**a**), CPR2 with 2-CP (**b**) and CPDR with 2-CP (**c**), PR2 with 2-CP (**d**) and PDR with 2-CP (**e**). Δ*H* is calculated at 0 K.

Figure S2. The optimized geometries for transition states for pre-PCTA formation routes from the S/C coupling of cross-condensation reactions of CTPR1 with 2-CTP. The red dotted line represents intramolecular hydrogen bonds ([a]Reference [24], previously calculated by Dar et al. at B3LYP/6-311+G(d,p) level of theory)

Figure S3. MPWB1K/6-31+G(d,p) optimized geometries for transition states of the coupling reactions of CTPR1 with 2-CTP, CTPR2 with 2-CTP, CTPDR with 2-CTP, CPR1 with 2-CP and PR2 with 2-CP. Distances are in Å.

Table S1. Imaginary frequencies (in cm^{-1}), zero point energies (ZPE, in a.u.) and total energies (without ZPE, in a.u.) for transition states involved in the formation of pre-PCTA/PT/DT/DF intermediates.

Table S2. TST rate constants for for pre-PCTA/PT/DT/DF formation routes from the cross-condensation reactions of 2-C(T)P with C(T)PR1, C(T)PR2 and (T)PR2, and C(T)PDR and (T)PDR over the temperature range of 600–1200 K (units are cm^3 $molecule^{-1}$ s^{-1}).

Table S3. Activation enthalpies ($\Delta H^{\neq}$), activation Gibbs free energies ($\Delta G^{\neq}$), activation entropies ($\Delta S^{\neq}$), relative enthalpies ($\Delta_r H$), relative Gibbs free energies ($\Delta_r G$), relative entropies ($\Delta_r S$) calculated at 298.15 K and 1 atm. (unit is kcal /mol for $\Delta G^{\neq}$, $\Delta H^{\neq}$, $\Delta_r G$ and $\Delta_r H$, unit is cal mol^{-1} K^{-1} for $\Delta S^{\neq}$ and $\Delta_r S$)

Table S4. Cartesian coordinates for transition states involved in the formation routes of pre-intermediates for PCTA/PT/DT/DFs, x coordinate, y coordinate and z coordinate.

Table S5. Cartesian coordinates for reactions, intermediate and products involved in the formation routes of pre-intermediates for PCTA/PT/DT/DFs, x coordinate, y coordinate and z coordinate.

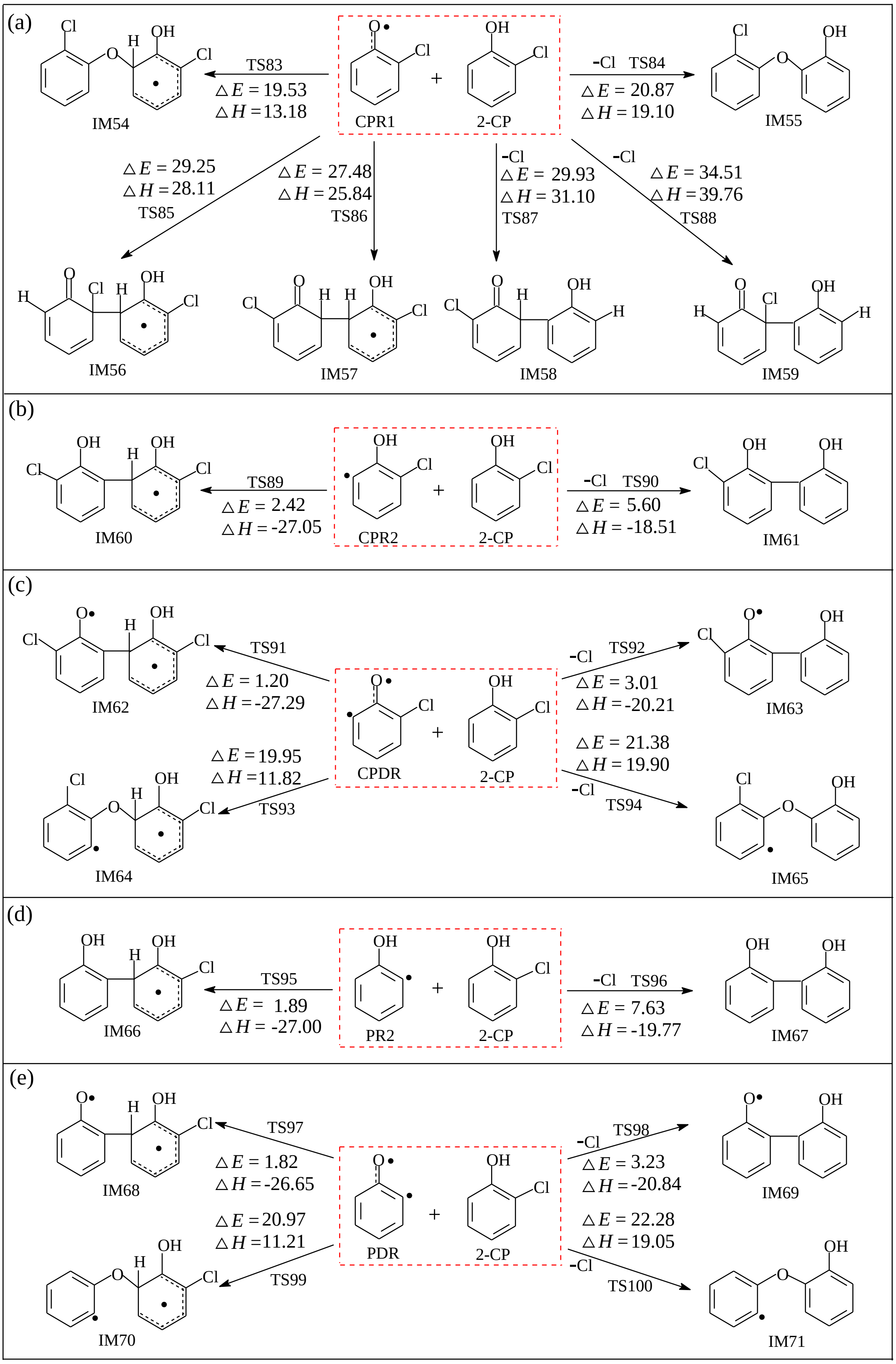

Figure S1. Pre-PCDD/DF formation pathways embedded with the potential barriers Δ*E* (in kcal/mol) and reaction heats Δ*H* (in kcal/mol) from the coupling reactions of CPR1 with 2-CP (**a**), CPR2 with 2-CP (**b**) and CPDR with 2-CP (**c**), PR2 with 2-CP (**d**) and PDR with 2-CP (**e**). Δ*H* is calculated at 0 K.

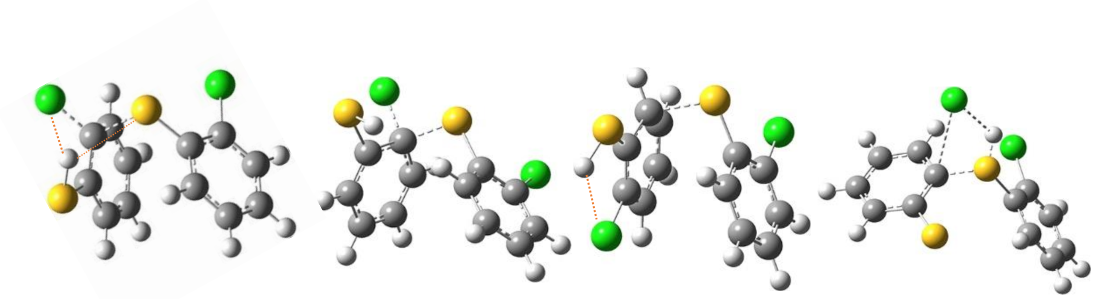

TS36 **TS-6**[a] **TS35** **TS-7**[a]

Figure S2. The optimized geometries for transition states for pre-PCTA formation routes from the S/C coupling of cross-condensation reactions of CTPR1 with 2-CTP. The red dotted line represents intramolecular hydrogen bonds ([a]Reference [24], previously calculated by Dar et al. at B3LYP/6-311+G(d,p) level of theory)

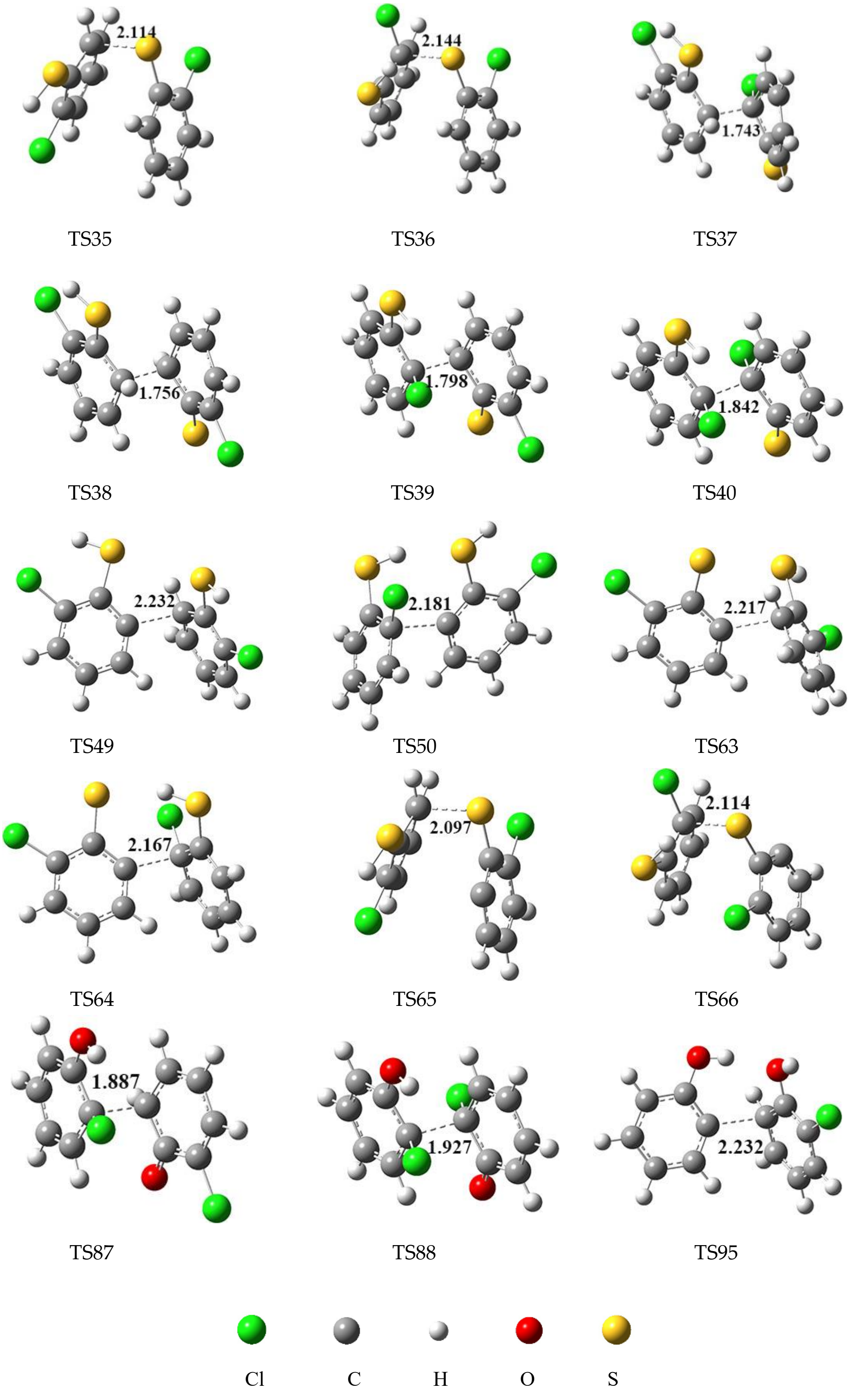

Figure S3. MPWB1K/6-31+G(d,p) optimized geometries for transition states of the coupling reactions of CTPR1 with 2-CTP, CTPR2 with 2-CTP, CTPDR with 2-CTP, CPR1 with 2-CP and PR2 with 2-CP. Distances are in Å.

Table S1. Imaginary frequencies (in cm^{-1}), zero point energies (ZPE, in a.u.) and total energies (without ZPE, in a.u.) for transition states involved in the formation of pre-PCTA/PT/DT/DF intermediates.

TS	Imaginary Frequencies	ZPE	Total Energies
TS1	−938i	0.09316	−1090.60937
TS2	−105i	0.10337	−1165.79578
TS3	−888i	0.09229	−1090.62576
TS4	−761i	0.10215	−1165.86250
TS5	−980i	0.09043	−1090.60320
TS6	−1722i	0.09812	−1165.84924
TS7	−853i	0.08834	−630.25694
TS8	−741i	0.09850	−705.49463
TS9	−936i	0.08393	−1089.96518
TS10	−142i	0.09384	−1165.16275
TS11	−1025i	0.08102	−1089.96954
TS12	−1767i	0.08845	−1165.21758
TS13	−886i	0.07910	−1089.93839
TS14	−791i	0.08886	−1165.17547
TS15	−910i	0.09904	−767.57783
TS16	−4i	0.10921	−842.76241
TS17	−2222i	0.09446	−767.57658
TS18	−2012i	0.10439	−842.82259
TS19	−896i	0.09627	−767.57039
TS20	−1767i	0.10362	−842.81822
TS21	−2182i	0.09065	−307.20602
TS22	−1428i	0.10143	−382.45499
TS23	−915i	0.08503	−766.92722
TS24	−125i	0.09485	−842.11252
TS25	−964i	0.08220	−766.92213
TS26	−1781i	0.09046	−842.17120
TS27	−2209i	0.08138	−766.88792
TS28	−1582i	0.09170	−842.13484
TS29	−625i	0.17903	−1856.55767
TS30	−636i	0.17850	−1856.55300
TS31	−564i	0.17813	−1856.53843
TS32	−584i	0.17867	−1856.54274
TS33	−647i	0.17836	−1856.53692
TS34	−601i	0.17825	−1856.52944
TS35	−337i	0.17673	−2179.62296
TS36	−399i	0.17624	−2179.61632
TS37	−275i	0.17571	−2179.56684
TS38	−384i	0.17615	−2179.57226
TS39	−547i	0.17585	−2179.56605
TS40	−545i	0.17559	−2179.55783

Table S1. *Cont*.

TS	Imaginary Frequencies	ZPE	Total Energies
TS41	−325i	0.18212	−1856.59007
TS42	−382i	0.18174	−1856.58447
TS43	−215i	0.18139	−1856.53709
TS44	−287i	0.18218	−1856.54196
TS45	−500i	0.18186	−1856.53736
TS46	−503i	0.18150	−1856.52936
TS47	−295i	0.17848	−1856.54362
TS48	−484i	0.17869	−1856.53370
TS49	−339i	0.17306	−2179.57473
TS50	−531i	0.17317	−2179.56316
TS51	−328i	0.17847	−1856.54353
TS52	−484i	0.17877	−1856.53534
TS53	−359i	0.18786	−1396.85838
TS54	−512i	0.18837	−1396.85110
TS55	−358i	0.18243	−1719.89213
TS56	−532i	0.18307	−1719.87982
TS57	−349i	0.18819	−1396.86203
TS58	−491i	0.18808	−1396.85247
TS59	−281i	0.16471	−1855.89841
TS60	−484i	0.16434	−1855.88904
TS61	−615i	0.16568	−1855.86993
TS62	−630i	0.16469	−1855.86513
TS63	−346i	0.16339	−2178.94438
TS64	−533i	0.16328	−2178.92911
TS65	−318i	0.16293	−2178.93558
TS66	−375i	0.16279	−2178.92802
TS67	−344i	0.16860	−1855.91178
TS68	−491i	0.16835	−1855.90371
TS69	−292i	0.16871	−1855.90348
TS70	−358i	0.16858	−1855.89774
TS71	−306i	0.17401	−1396.21549
TS72	−493i	0.17429	−1396.20684
TS73	−643i	0.17485	−1396.18659
TS74	−651i	0.17389	−1396.18234
TS75	−340i	0.17319	−1719.26436
TS76	−528i	0.17324	−1719.25053
TS77	−342i	0.17230	−1719.25362
TS78	−409i	0.17260	−1719.24825
TS79	−341i	0.17846	−1396.23194
TS80	−494i	0.17860	−1396.22480
TS81	−326i	0.17835	−1396.22144
TS82	−390i	0.17810	−1396.21651

Table S1. *Cont*.

TS	Imaginary Frequencies	ZPE	Total Energies
TS83	−611i	0.18411	−1533.52428
TS84	−613i	0.18378	−1533.52182
TS85	−537i	0.18347	−1533.50815
TS86	−573i	0.18434	−1533.51184
TS87	−623i	0.18375	−1533.50736
TS88	−588i	0.18372	−1533.50002
TS89	−286i	0.18377	−1533.51117
TS90	−442i	0.18394	−1533.50628
TS91	−287i	0.17014	−1532.86594
TS92	−435i	0.17047	−1532.86338
TS93	−604i	0.17119	−1532.83709
TS94	−606i	0.17053	−1532.83415
TS95	−358i	0.19363	−1073.82954
TS96	−458i	0.19292	−1073.81968
TS97	−293i	0.17960	−1073.18328
TS98	−442i	0.18006	−1073.18148
TS99	−629i	0.18032	−1073.15346
TS100	−632i	0.17994	−1073.15100

Table S2. TST rate constants for for pre-PCTA/PT/DT/DF formation routes from the cross-condensation reactions of 2-C(T)P with C(T)PR1, C(T)PR2 and (T)PR2, and C(T)PDR and (T)PDR over the temperature range of 600–1200 K (units are cm^3 $molecule^{-1}$ s^{-1}).

T(K)	TST Rate Constants			
	TS29	TS30	TS31	TS32
600	1.91×10^{-23}	1.69×10^{-24}	6.42×10^{-28}	1.47×10^{-26}
700	2.40×10^{-22}	2.92×10^{-23}	3.31×10^{-26}	5.59×10^{-25}
800	1.68×10^{-21}	2.62×10^{-22}	6.72×10^{-25}	9.06×10^{-24}
900	7.99×10^{-21}	1.50×10^{-21}	7.30×10^{-24}	8.24×10^{-23}
1000	2.87×10^{-20}	6.29×10^{-21}	5.09×10^{-23}	4.98×10^{-22}
1100	8.42×10^{-20}	2.09×10^{-20}	2.57×10^{-22}	2.23×10^{-21}
1200	2.11×10^{-19}	5.82×10^{-20}	1.01×10^{-21}	7.97×10^{-21}
	TS33	TS34	TS35	TS36
600	2.79×10^{-28}	1.55×10^{-30}	6.32×10^{-17}	1.46×10^{-18}
700	1.62×10^{-26}	1.57×10^{-28}	9.39×10^{-17}	3.50×10^{-18}
800	3.59×10^{-25}	5.28×10^{-27}	1.34×10^{-16}	7.14×10^{-18}
900	4.18×10^{-24}	8.51×10^{-26}	1.84×10^{-16}	1.30×10^{-17}
1000	3.08×10^{-23}	8.51×10^{-26}	2.45×10^{-16}	1.30×10^{-17}
1100`	1.62×10^{-22}	5.32×10^{-24}	3.20×10^{-16}	3.42×10^{-17}
1200	6.64×10^{-22}	2.60×10^{-23}	4.08×10^{-16}	5.10×10^{-17}
	TS37	TS38	TS39	TS40
600	8.21×10^{-30}	4.82×10^{-28}	7.43×10^{-30}	2.88×10^{-32}
700	7.80×10^{-28}	3.11×10^{-26}	7.53×10^{-28}	5.31×10^{-30}
800	2.52×10^{-26}	7.50×10^{-25}	2.55×10^{-26}	2.82×10^{-28}
900	3.93×10^{-25}	9.32×10^{-24}	4.13×10^{-25}	6.51×10^{-27}
1000	3.67×10^{-24}	7.24×10^{-23}	3.98×10^{-24}	8.32×10^{-26}
1100	2.35×10^{-23}	3.99×10^{-22}	2.62×10^{-23}	6.90×10^{-25}
1200	1.14×10^{-22}	1.70×10^{-21}	1.29×10^{-22}	4.12×10^{-24}
	TS41	TS42	TS43	TS44
600	1.20×10^{-16}	5.93×10^{-18}	6.96×10^{-29}	1.28×10^{-27}
700	2.04×10^{-16}	1.51×10^{-17}	6.02×10^{-27}	7.94×10^{-26}
800	3.20×10^{-16}	3.24×10^{-17}	1.81×10^{-25}	1.86×10^{-24}
900	4.76×10^{-16}	6.13×10^{-17}	2.68×10^{-24}	2.25×10^{-23}
1000	6.76×10^{-16}	1.06×10^{-16}	2.39×10^{-23}	1.72×10^{-22}
1100	9.28×10^{-16}	1.70×10^{-16}	1.48×10^{-22}	9.31×10^{-22}
1200	1.24×10^{-15}	2.59×10^{-16}	6.91×10^{-22}	3.90×10^{-21}
	TS45	TS46	TS47	TS48
600	5.33×10^{-29}	2.54×10^{-31}	9.20×10^{-17}	1.33×10^{-19}
700	4.58×10^{-27}	3.90×10^{-29}	1.68×10^{-16}	5.02×10^{-19}
800	1.37×10^{-25}	1.81×10^{-27}	2.79×10^{-16}	1.44×10^{-18}
900	2.02×10^{-24}	3.76×10^{-26}	4.31×10^{-16}	3.40×10^{-18}
1000	1.80×10^{-23}	4.41×10^{-25}	6.32×10^{-16}	7.01×10^{-18}
1100	1.11×10^{-22}	3.40×10^{-24}	8.89×10^{-16}	1.30×10^{-17}
1200	5.16×10^{-22}	1.92×10^{-23}	1.21×10^{-15}	2.23×10^{-17}

Table S2. *Cont.*

T(K)	TST Rate Constants			
	TS49	TS50	TS51	TS52
600	1.05×10^{-17}	4.78×10^{-21}	4.31×10^{-17}	1.15×10^{-19}
700	2.39×10^{-17}	2.51×10^{-20}	9.85×10^{-17}	4.77×10^{-19}
800	4.68×10^{-17}	9.18×10^{-20}	1.93×10^{-16}	1.46×10^{-18}
900	8.20×10^{-17}	2.63×10^{-19}	3.40×10^{-16}	3.64×10^{-18}
1000	1.33×10^{-16}	6.31×10^{-19}	5.54×10^{-16}	7.82×10^{-18}
1100	2.03×10^{-16}	1.33×10^{-18}	8.48×10^{-16}	1.50×10^{-17}
1200	2.96×10^{-16}	2.53×10^{-18}	1.24×10^{-15}	2.65×10^{-17}
	TS53	TS54	TS55	TS56
600	3.07×10^{-17}	7.81×10^{-20}	1.32×10^{-17}	2.18×10^{-21}
700	6.44×10^{-17}	2.85×10^{-19}	3.10×10^{-17}	1.28×10^{-20}
800	1.19×10^{-16}	7.93×10^{-19}	6.22×10^{-17}	5.11×10^{-20}
900	1.99×10^{-16}	1.84×10^{-18}	1.12×10^{-16}	1.56×10^{-19}
1000	3.11×10^{-16}	3.72×10^{-18}	1.84×10^{-16}	3.95×10^{-19}
1100	4.61×10^{-16}	6.81×10^{-18}	2.86×10^{-16}	8.69×10^{-19}
1200	6.56×10^{-16}	1.15×10^{-17}	4.21×10^{-16}	1.72×10^{-18}
	TS57	TS58	TS59	TS60
600	8.31×10^{-17}	1.28×10^{-19}	6.37×10^{-16}	1.63×10^{-18}
700	1.83×10^{-16}	5.61×10^{-19}	9.99×10^{-16}	4.94×10^{-18}
800	3.50×10^{-16}	1.79×10^{-18}	1.47×10^{-15}	1.20×10^{-17}
900	6.05×10^{-16}	4.62×10^{-18}	2.08×10^{-15}	2.50×10^{-17}
1000	9.70×10^{-16}	1.02×10^{-17}	2.84×10^{-15}	4.65×10^{-17}
1100	1.47×10^{-15}	2.00×10^{-17}	3.76×10^{-15}	7.96×10^{-17}
1200	2.12×10^{-15}	3.60×10^{-17}	4.86×10^{-15}	1.27×10^{-16}
	TS61	TS62	TS63	TS64
600	1.40×10^{-23}	3.10×10^{-24}	5.39×10^{-17}	5.15×10^{-21}
700	1.86×10^{-22}	5.71×10^{-23}	1.02×10^{-16}	2.95×10^{-20}
800	1.37×10^{-21}	5.37×10^{-22}	1.73×10^{-16}	1.15×10^{-19}
900	6.75×10^{-21}	3.21×10^{-21}	2.73×10^{-16}	3.49×10^{-19}
1000	2.50×10^{-20}	1.39×10^{-20}	4.07×10^{-16}	8.75×10^{-19}
1100	7.49×10^{-20}	4.74×10^{-20}	5.80×10^{-16}	1.91×10^{-18}
1200	1.91×10^{-19}	1.35×10^{-19}	7.97×10^{-16}	3.75×10^{-18}
	TS65	TS66	TS67	TS68
600	3.32×10^{-19}	1.72×10^{-21}	3.47×10^{-16}	8.04×10^{-19}
700	1.18×10^{-18}	1.12×10^{-20}	7.26×10^{-16}	2.96×10^{-18}
800	3.24×10^{-18}	4.84×10^{-20}	1.34×10^{-15}	8.31×10^{-18}
900	7.42×10^{-18}	1.58×10^{-19}	2.25×10^{-15}	1.94×10^{-17}
1000	1.49×10^{-17}	4.20×10^{-19}	3.52×10^{-15}	3.95×10^{-17}
1100	2.71×10^{-17}	9.65×10^{-19}	5.23×10^{-15}	7.29×10^{-17}
1200	4.57×10^{-17}	1.97×10^{-18}	7.44×10^{-15}	1.24×10^{-16}

Table S2. *Cont.*

T(K)	TST Rate Constants			
	TS69	TS70	TS71	TS72
600	4.30×10^{-19}	6.46×10^{-21}	3.78×10^{-16}	5.53×10^{-19}
700	1.66×10^{-18}	4.39×10^{-20}	6.42×10^{-16}	1.76×10^{-18}
800	4.86×10^{-18}	1.96×10^{-19}	1.01×10^{-15}	4.43×10^{-18}
900	1.17×10^{-17}	6.56×10^{-19}	1.49×10^{-15}	9.50×10^{-18}
1000	2.44×10^{-17}	1.78×10^{-18}	2.12×10^{-15}	1.81×10^{-17}
1100	4.59×10^{-17}	4.17×10^{-18}	2.89×10^{-15}	3.15×10^{-17}
1200	7.94×10^{-17}	8.65×10^{-18}	3.84×10^{-15}	5.11×10^{-17}
	TS73	TS74	TS75	TS76
600	1.70×10^{-23}	8.75×10^{-24}	6.55×10^{-17}	7.93×10^{-21}
700	2.55×10^{-22}	1.74×10^{-22}	1.25×10^{-16}	4.12×10^{-20}
800	2.04×10^{-21}	1.73×10^{-21}	2.14×10^{-16}	1.50×10^{-19}
900	1.08×10^{-20}	1.08×10^{-20}	3.40×10^{-16}	4.27×10^{-19}
1000	4.21×10^{-20}	4.86×10^{-20}	5.08×10^{-16}	1.02×10^{-18}
1100	1.32×10^{-19}	1.71×10^{-19}	7.26×10^{-16}	2.15×10^{-18}
1200	3.50×10^{-19}	4.98×10^{-19}	1.00×10^{-15}	4.08×10^{-18}
	TS77	TS78	TS79	TS80
600	9.16×10^{-19}	3.23×10^{-21}	2.26×10^{-16}	6.95×10^{-19}
700	3.76×10^{-18}	2.34×10^{-20}	4.71×10^{-16}	2.40×10^{-18}
800	1.15×10^{-17}	1.09×10^{-19}	8.63×10^{-16}	6.44×10^{-18}
900	2.85×10^{-17}	3.78×10^{-19}	1.44×10^{-15}	1.45×10^{-17}
1000	6.12×10^{-17}	1.06×10^{-18}	2.26×10^{-15}	2.86×10^{-17}
1100	1.18×10^{-16}	2.53×10^{-18}	3.34×10^{-15}	5.13×10^{-17}
1200	2.08×10^{-16}	5.36×10^{-18}	4.74×10^{-15}	8.55×10^{-17}
	TS81	TS82		
600	2.31×10^{-19}	8.96×10^{-21}		
700	1.04×10^{-18}	6.79×10^{-20}		
800	3.40×10^{-18}	3.29×10^{-19}		
900	8.91×10^{-18}	1.17×10^{-18}		
1000	2.00×10^{-17}	3.36×10^{-18}		
1100	3.97×10^{-17}	8.18×10^{-18}		
1200	7.21×10^{-17}	1.76×10^{-17}		

Table S3. Activation enthalpies ($\Delta H^{\neq}$), activation Gibbs free energies ($\Delta G^{\neq}$), activation entropies ($\Delta S^{\neq}$), relative enthalpies ($\Delta_r H$), relative Gibbs free energies ($\Delta_r G$), relative entropies ($\Delta_r S$) calculated at 298.15 K and 1 atm. (unit is kcal /mol for $\Delta G^{\neq}$, $\Delta H^{\neq}$, $\Delta_r G$ and $\Delta_r H$, unit is cal mol^{-1} K^{-1} for $\Delta S^{\neq}$ and $\Delta_r S$)

**Reactions**	$\Delta H^{\neq}$	$\Delta G^{\neq}$	$\Delta S^{\neq}$	$\Delta_r H$	$\Delta_r G$	$\Delta_r S$
2-CTP + H → TPR2 + HCl via TS1	13.36	20.25	−23.11	−5.16	−8.06	9.72
2-CTP + OH → TPR2 + HClO via TS2	42.15	49.96	−26.17	44.17	42.28	6.35
2-CTP + H → CTPR1 + H_2 via TS3	2.25	9.72	−25.07	−14.33	−14.64	1.04
2-CTP + OH → CTPR1 + H_2O via TS4	−0.74	7.97	−29.24	−28.15	−28.09	−0.20
2-CTP + H → CTPR2 + H_2 via TS5	15.50	22.45	−23.29	10.50	9.65	2.87
2-CTP + OH → CTPR2 + H_2O via TS6	5.01	14.39	−31.45	−3.32	−3.81	1.63
TPR2 + H → TPDR + H_2 via TS7	1.95	8.36	−21.49	−22.92	−23.67	2.53
TPR2 + OH → TPDR + H_2O via TS8	−1.53	6.01	−25.28	−36.75	−37.13	1.29
CTPR1 + H → TPDR + HCl via TS9	13.34	19.72	−21.39	−13.75	−17.09	11.21
CTPR1 + OH → TPDR + HClO via TS10	34.95	41.73	−22.74	35.58	33.24	7.83
CTPR1 + H → CTPDR + H_2 via TS11	8.68	15.34	−22.35	3.12	1.78	4.49
CTPR1 + OH → CTPDR + H_2O via TS12	−3.20	5.83	−30.29	−10.71	−11.67	3.25
CTPR2 + H → CTPDR + H_2 via TS13	2.31	9.35	−23.60	−21.71	−22.50	2.66
CTPR2 + OH → CTPDR + H_2O via TS14	−0.92	7.28	−27.48	−35.54	−35.96	1.42
2-CP + H → PR2 + HCl via TS15	13.73	20.12	−21.43	−2.79	−6.36	11.97
2-CP + OH → PR2 + HClO via TS16	43.65	50.84	−24.13	46.54	43.97	8.59
2-CP + H → CPR1 + H_2 via TS17	11.49	18.10	−22.18	−12.89	−14.34	4.87
2-CP + OH → CPR1 + H_2O via TS18	2.45	11.40	−30.04	−26.71	−27.79	3.62
2-CP + H → CPR2 + H_2 via TS19	16.72	23.07	−21.30	12.34	10.83	5.06
2-CP + OH → CPR2 + H_2O via TS20	4.91	13.59	−29.10	−1.49	−2.63	3.82
PR2 + H → PDR + H_2 via TS21	9.86	16.61	−22.63	−15.81	−16.95	3.83
PR2 + OH → PDR + H_2O via TS22	−0.40	8.29	−29.14	−29.63	−30.41	2.59
CPR1 + H → PDR + HCl via TS23	13.56	20.07	−21.81	−5.72	−8.98	10.93
CPR1 + OH → PDR + HClO via TS24	42.89	49.65	−22.68	43.61	41.36	7.56
CPR1 + H → CPDR + H_2 via TS25	15.00	21.50	−21.80	10.22	8.89	4.47
CPR1 + OH → CPDR + H_2O via TS26	2.80	12.15	−31.36	−3.61	−4.57	3.23
CPR2 + H → CPDR + H_2 via TS27	10.60	17.44	−22.97	−15.00	−16.28	4.28
CPR2 + OH → CPDR + H_2O via TS28	1.34	10.25	−29.86	−28.83	−29.73	3.03
CPR1 + 2-CTP → IM1 via TS29	18.26	32.13	−46.49	11.21	24.53	−44.69
CPR1 + 2-CTP → IM2 + Cl via TS30	20.83	34.88	−47.10	18.15	22.39	−14.22
CPR1 + 2-CTP → IM3 via TS31	28.74	42.76	−47.04	28.74	42.76	−47.04
CPR1 + 2-CTP → IM4 + Cl via TS32	25.77	38.82	−43.79	25.77	38.82	−43.79
CPR1 + 2-CTP → IM5 via TS33	30.90	45.12	−47.68	32.17	37.50	−17.89
CPR1 + 2-CTP → IM6 + Cl TS34	35.39	50.46	−50.56	41.44	47.02	−18.73
CTPR1 + 2-CTP → IM7 via TS35	0.15	14.12	−46.83	−0.52	13.05	−45.49
CTPR1 + 2-CTP → IM8 via TS36	4.05	18.34	−47.93	11.01	15.85	−16.24
CTPR1 + 2-CTP → IM9 via TS37	34.67	49.08	−48.34	35.34	49.23	−46.59
CTPR1 + 2-CTP → IM10 via TS38	32.22	45.36	−44.07	32.22	45.36	−44.07
CTPR1 + 2-CTP → IM11 via TS39	35.34	49.50	−47.51	40.18	44.92	−15.89
CTPR1 + 2-CTP → IM12 + Cl TS40	40.18	55.25	−50.57	50.95	56.32	−18.01
CTPR1 + 2-CP → IM13 via TS41	0.70	13.32	−42.35	0.70	13.32	−42.35
CTPR1 + 2-CP → IM14 + Cl via TS42	4.50	17.75	−44.43	10.13	13.60	−11.67
CTPR1 + 2-CP → IM15 via TS43	34.56	47.41	−43.11	34.56	47.41	−43.11
CTPR1 + 2-CP → IM16 + Cl via TS44	32.02	44.54	−41.99	32.02	44.54	−41.99

Table S3. *Cont.*

Reactions	$\Delta H^{\neq}$	$\Delta G^{\neq}$	$\Delta S^{\neq}$	$\Delta_r H$	$\Delta_r G$	$\Delta_r S$
CTPR1 + 2-CP → IM17 via TS45	33.98	47.64	−45.84	39.91	44.70	−16.07
CTPR1 + 2-CP → IM18 + Cl TS46	38.68	53.20	−48.71	47.19	52.09	−16.43
CPR2 + 2-CTP → IM19 via TS47	−27.85	−14.30	−45.43	−27.85	−14.30	−45.43
CPR2 + 2-CTP → IM20 + Cl via TS48	8.03	21.74	−46.00	−17.95	−12.81	−17.25
CTPR2 + 2-CTP → IM21 via TS49	3.76	16.99	−44.36	−25.11	−11.91	−44.29
CTPR2 + 2-CTP → IM22 + Cl via TS50	10.82	25.12	−47.94	−16.87	−11.62	−17.59
CTPR2 + 2-CP → IM23 via TS51	3.73	16.15	−41.66	−26.04	−11.48	−48.85
CTPR2 + 2-CP → IM24 + Cl TS52	8.80	22.21	−44.99	−16.30	−11.96	−14.55
PR2 + 2-CTP → IM25 via TS53	−25.80	−11.91	−46.58	−25.80	−11.91	−46.58
PR2 + 2-CTP → IM26 + Cl via TS54	7.80	21.94	−47.40	−19.69	−14.07	−18.85
TPR2 + 2-CTP → IM27 via TS55	3.99	16.98	−43.56	−25.43	−11.83	−45.62
TPR2 + 2-CTP → IM28 + Cl via TS56	11.79	26.06	−47.88	−17.46	−11.82	−18.92
TPR2 + 2-CP → IM29 via TS57	3.44	15.62	−40.85	−25.43	−11.98	−45.09
TPR2 + 2-CP → IM26 + Cl via TS58	9.22	22.37	−44.11	−17.33	−12.37	−16.61
CPDR + 2-CTP → IM30 via TS59	−27.58	−14.38	−44.25	−27.58	−14.38	−44.25
CPDR + 2-CTP → IM31 + Cl viaTS60	6.12	19.31	−44.25	−17.54	−10.94	−22.16
CPDR + 2-CTP → IM32 via TS61	10.05	23.42	−44.87	10.05	23.42	−44.87
CPDR + 2-CTP → IM33 + Cl via TS62	21.30	34.77	−45.18	19.02	23.41	−14.75
CTPDR + 2-CTP → IM34 via TS63	2.16	15.22	−43.82	−25.03	−11.55	−45.23
CTPDR + 2-CTP → IM35 + Cl via TS64	11.48	25.42	−46.74	−9.03	−3.84	−17.40
CTPDR + 2-CTP → IM36 via TS65	7.36	20.87	−45.31	7.20	20.52	−44.69
CTPDR + 2-CTP → IM37 + Cl via TS66	11.88	26.09	−47.65	18.35	22.75	−14.76
CTPDR + 2-CP → IM38 via TS67	2.94	14.53	−38.88	−24.22	−11.31	−43.30
CTPDR + 2-CP → IM39 + Cl via TS68	7.70	20.54	−43.06	−8.37	−4.09	−14.35
CTPDR + 2-CP → IM40 via TS69	8.03	21.06	−43.67	8.02	20.63	−42.30
CTPDR + 2-CP → IM41 + Cl TS70	11.51	25.24	−46.07	17.62	20.77	−10.55
PDR + 2-CTP → IM42 via TS71	−27.61	−14.25	−44.82	−27.61	−14.25	−44.82
PDR + 2-CTP → IM43 + Cl via TS72	6.60	20.18	−45.56	−16.68	−11.20	−18.37
PDR + 2-CTP → IM44 via TS73	9.48	22.48	−43.60	9.48	22.48	−43.60
PDR + 2-CTP → IM45 + Cl TS74	21.92	34.47	−42.09	18.01	22.18	−13.98
TPDR + 2-CTP → IM46 via TS75	2.26	15.16	−43.26	−26.09	−13.01	−43.86
TPDR + 2-CTP → IM47 + Cl via TS76	10.72	24.77	−47.13	−15.02	−10.03	−16.74
TPDR + 2-CTP → IM48 via TS77	8.52	20.86	−41.38	7.93	20.57	−42.38
TPDR + 2-CTP → IM49 + Cl TS78	11.90	25.69	−46.27	19.92	23.85	−13.16
TPDR + 2-CP → IM50 via TS79	2.92	14.77	−39.75	−24.93	−12.71	−40.98
TPDR + 2-CP → IM51 + Cl via TS80	7.28	20.39	−43.99	−7.22	−2.86	−14.61
TPDR + 2-CP → IM52 via TS81	9.30	22.06	−42.80	9.01	21.26	−41.08
TPDR + 2-CP → IM53 + Cl TS82	12.30	25.19	−43.23	19.06	21.67	−8.76

Table S4. Cartesian coordinates for transition states involved in the formation routes of pre-intermediates for PCTA/PT/DT/DFs, x coordinate, y coordinate and z coordinate.

TS1			
0	2		
C	2.25897600	−1.14838400	0.00017200
C	0.93989600	−1.56578900	−0.00006100
C	−0.05451600	−0.61656600	−0.00046000
C	0.18476500	0.74289100	−0.00002200
C	1.52303200	1.13851600	0.00020800
C	2.54251900	0.20802200	0.00030800
H	3.05488100	−1.87624700	0.00030200
H	0.68442500	−2.61351800	−0.00010600
H	1.75510600	2.19328300	0.00037000
H	3.56633800	0.54781700	0.00055000
Cl	−1.84986500	−1.30490100	−0.00076700
H	−3.30857400	−1.87059100	0.01112500
S	−1.03610000	2.00350500	0.00002000
H	−2.09489600	1.19434700	−0.00039100
TS2			
0	2		
C	−2.18101900	−1.87355600	0.01179200
C	−0.79571100	−1.75420300	0.00122200
C	−0.29505100	−0.48522600	−0.00408500
C	−1.00786100	0.68273900	0.00340900
C	−2.39608000	0.52878900	0.01348000
C	−2.96589000	−0.73128300	0.01929200
H	−2.63512200	−2.85228100	0.01370300
H	−0.15157000	−2.61944200	−0.00493300
H	−3.02328500	1.40844800	0.01503600
H	−4.04093200	−0.82041700	0.02778300
Cl	2.15510500	−0.47863900	−0.07555500
O	3.87864200	−0.40404600	0.00103500
H	4.07080400	−0.56221500	0.92851400
S	−0.31199900	2.29471800	0.00810200
H	0.95584200	1.89609700	−0.10424200
TS3			
0	2		
C	−0.06851500	0.70050500	−0.02142300
C	−1.30695500	1.31615300	0.01874200
C	−2.45786700	0.55104300	0.04174600
C	−2.36904300	−0.82987800	0.03927900
C	−1.12947200	−1.43860400	0.02294200

C	0.04214000	−0.68894700	−0.01638700
H	−1.35747000	2.39295100	0.02187900
H	−3.41988300	1.03828300	0.06467200
H	−3.26228200	−1.43385800	0.06419500
H	−1.05043800	−2.51407300	0.04595500
H	2.22814800	−0.88929400	0.89238800
Cl	1.33852200	1.69602100	−0.06977800
H	2.71771400	−0.31937000	1.94132300
S	1.57047600	−1.54803900	−0.14709800
TS4			
0	2		
C	−0.47384100	0.73296400	−0.09650600
C	−1.76264800	1.10007100	0.24675100
C	−2.73941700	0.13517200	0.40632000
C	−2.42580200	−1.20184100	0.23267600
C	−1.13384100	−1.56640900	−0.08909300
C	−0.13783600	−0.60996900	−0.26481700
H	−1.99092200	2.14517000	0.37907000
H	−3.74273600	0.43238400	0.66783800
H	−3.18130400	−1.96090600	0.35970700
H	−0.87550500	−2.60748900	−0.20479900
H	2.20861700	−0.32317500	0.02581000
Cl	0.70180900	1.97158200	−0.30701400
O	2.73414800	−0.36003500	1.47825200
H	2.65580900	−1.26231300	1.81181900
S	1.44765100	−1.16251300	−0.76613700
TS5			
0	2		
C	0.08290900	−0.74144700	−0.00008300
C	0.02627900	0.65301100	0.00235500
C	−1.24408800	1.18851200	0.00104000
C	−2.41048100	0.47659700	−0.00211300
C	−2.31620100	−0.90954600	−0.00144500
C	−1.07163700	−1.50830600	0.00130900
H	−1.35526500	2.66277000	0.01997900
H	−3.36761000	0.97495900	−0.00238200
H	−3.20619600	−1.51924500	−0.00210800
H	−0.97942200	−2.58209300	0.00220400
Cl	1.60088600	−1.57198800	−0.00069800
H	−1.42170300	3.50141500	0.03414600
S	1.39878700	1.74551200	−0.01038500
H	2.33384800	0.80486000	0.11981700

TS6			
0	2		
C	−1.06879600	0.32112200	0.00389000
C	0.19905600	−0.26048500	−0.02598400
C	1.26862100	0.61380900	−0.04677000
C	1.15381900	1.98043500	−0.04184600
C	−0.12073100	2.52680600	−0.00633600
C	−1.22471100	1.69640700	0.02257900
H	2.43368300	0.15021200	−0.07486600
H	2.03209700	2.60563500	−0.05449400
H	−0.25476800	3.59698400	0.00318400
H	−2.22126800	2.10581600	0.05408100
Cl	−2.48614600	−0.66436000	0.03089800
O	3.50262900	−0.38980200	0.06302100
H	3.24853600	−1.15236300	0.59524700
S	0.52146300	−1.98676900	−0.09575800
H	−0.58177200	−2.37400400	0.54634600
TS7			
0	3		
C	−0.22041400	1.14017100	−0.13506900
C	−1.57840900	1.27313000	−0.09073000
C	−2.31486000	0.09718400	0.02789800
C	−1.66349300	−1.12341200	0.10758100
C	−0.27998600	−1.19532900	0.08391600
C	0.47941800	−0.03313900	−0.04836100
H	−2.06430700	2.23448000	−0.15417900
H	−3.39307600	0.14039800	0.05858100
H	−2.23728500	−2.03196000	0.20325400
H	0.22338700	−2.14665800	0.17180500
H	2.49245800	0.87364700	0.79368800
H	2.62997400	1.74327500	1.75916700
S	2.23845700	−0.11030100	−0.15648300
TS8			
0	3		
C	−0.45627200	0.92274900	−0.59939800
C	−1.74072000	1.34907000	−0.42239000
C	−2.62104300	0.44805500	0.17046900
C	−2.17318500	−0.80574700	0.55922700
C	−0.85285400	−1.17787700	0.37635700
C	0.04661700	−0.29711100	−0.22857300
H	−2.06581900	2.33033000	−0.73164500

H	−3.65105200	0.72991800	0.32762000
H	−2.85797800	−1.49901800	1.02192200
H	−0.50674400	−2.14826100	0.70067500
H	2.28542800	0.44616400	−0.36634700
O	2.97166100	1.16517800	0.83534200
H	2.54306800	0.87281700	1.64905000
S	1.70403500	−0.79301300	−0.52613500
TS9			
0	3		
C	2.27709000	−1.08683400	0.00000000
C	0.96725600	−1.53852600	0.00004200
C	−0.03959400	−0.60683100	0.00005300
C	0.17207200	0.75541500	0.00002600
C	1.49676300	1.19243600	−0.00001600
C	2.53125700	0.27550500	−0.00003700
H	3.09077800	−1.79474400	−0.00000300
H	0.73364900	−2.59150000	0.00009600
H	1.71029600	2.25048900	−0.00004600
H	3.54881600	0.63391800	−0.00007900
Cl	−1.85623600	−1.21951900	−0.00003700
H	−3.33147400	−1.73245700	0.00005500
S	−1.16407000	1.87619500	0.00001200
TS10			
0	3		
C	−2.19433700	−1.79485300	0.00496300
C	−0.80422000	−1.71784600	0.00704100
C	−0.26984800	−0.46439100	0.00385100
C	−0.96108800	0.73788200	0.00210900
C	−2.37076300	0.60553800	0.00058400
C	−2.96616600	−0.63506100	0.00182300
H	−2.67266700	−2.76265200	0.00707400
H	−0.18805200	−2.60310600	0.01127200
H	−2.96138900	1.50882700	−0.00176200
H	−4.04205000	−0.71097300	0.00090200
Cl	2.11171800	−0.55158800	−0.02576100
O	3.83528800	−0.40132000	0.03160500
H	3.97338500	0.54938200	0.03405300
S	−0.20576300	2.26365500	0.00070800
TS11			
0	3		
C	0.20580800	−0.71033500	−0.00001000

C	−0.01607900	0.69300300	0.00000700
C	−1.35897100	1.07300500	0.00003000
C	−2.43000900	0.22609600	0.00003400
C	−2.16031700	−1.13813800	0.00001400
C	−0.85036000	−1.59819900	−0.00000800
H	−1.66078700	2.50762000	0.00002600
H	−3.44273500	0.59834800	0.00005000
H	−2.97072600	−1.85122600	0.00001700
H	−0.64577800	−2.65622800	−0.00002300
Cl	1.80009400	−1.34096400	−0.00003100
H	−1.87665500	3.32283300	−0.00001500
S	1.22841600	1.85015300	0.00000500
TS12			
0	3		
C	1.07993900	0.28013900	0.00007800
C	−0.21385300	−0.30448000	0.00001300
C	−1.28266500	0.60124500	0.00008300
C	−1.15247500	1.96239500	0.00006900
C	0.13521600	2.48735100	−0.00005100
C	1.24112100	1.64978700	−0.00008000
H	−2.45482700	0.14979700	0.00002800
H	−2.01829200	2.60460500	0.00026400
H	0.28215800	3.55644800	0.00006200
H	2.23731500	2.06067900	−0.00014900
Cl	2.47890300	−0.70794400	−0.00002400
O	−3.50601700	−0.42239800	−0.00011700
H	−3.17541900	−1.32951000	−0.00005000
S	−0.48799100	−1.98040100	0.00003200
TS13			
0	3		
C	−0.11199600	0.66340500	−0.02744400
C	0.07856100	−0.72333400	−0.02542200
C	−1.07670900	−1.45893800	0.00955100
C	−2.35755700	−0.99106500	0.04238100
C	−2.50025100	0.39233900	0.05134000
C	−1.38377400	1.20985300	0.02299900
H	−3.21168000	−1.64901800	0.06535100
H	−3.48456500	0.83420500	0.07903700
H	−1.49070200	2.28207300	0.03099200
Cl	1.24265700	1.72784700	−0.07495700
H	2.26372300	−0.78431600	0.86922400
H	2.74163600	−0.17357700	1.90546300

S	1.63542400	−1.52727100	−0.13226400
TS14			
0	3		
C	−0.49458400	0.69624000	−0.10384000
C	−0.11841800	−0.64166900	−0.28204000
C	−1.12547600	−1.55409100	−0.10401000
C	−2.42456800	−1.30089400	0.22321300
C	−2.75403200	0.03855700	0.40216100
C	−1.79417000	1.02394900	0.24351900
H	−3.15496400	−2.08518200	0.34211600
H	−3.76355700	0.31586700	0.66466300
H	−2.04893000	2.06163000	0.38260200
Cl	0.65421200	1.96006800	−0.30281600
H	2.20763500	−0.31397500	0.01532000
O	2.70984400	−0.33032300	1.48305200
H	2.65973900	−1.23254400	1.82246200
S	1.47295100	−1.18730700	−0.76360800
TS15			
0	2		
C	−1.34374900	−1.34374900	0.00000200
C	−1.46885400	−1.46885400	−0.00001900
C	−0.32462600	−0.32462600	−0.00005000
C	0.94803300	0.94803300	−0.00000900
C	1.05621100	1.05621100	0.00002500
C	−0.07931900	−0.07931900	0.00002600
H	−2.22589900	−2.22589900	0.00000900
H	−2.43585700	−2.43585700	−0.00002100
H	2.04367400	2.04367400	0.00004900
H	0.02550700	0.02550700	0.00005000
O	2.06871800	2.06871800	−0.00001900
H	1.84201300	1.84201300	−0.00001300
Cl	−0.46709300	−0.46709300	−0.00000700
H	−0.58478400	−0.58478400	0.00035000
TS16			
0	2		
C	2.49412900	−1.38891500	0.01082000
C	1.10783900	−1.51220100	−0.00825600
C	0.39219700	−0.35280600	−0.01398300
C	0.89922700	0.92259500	−0.00031400
C	2.28702100	1.01842300	0.01950000
C	3.06498600	−0.12557400	0.02510500

H	3.11212200	−2.27306300	0.01417400
H	0.62745000	−2.47838400	−0.01984400
H	2.73074100	2.00243100	0.02967500
H	4.13917700	−0.02652300	0.04043400
Cl	−2.19659100	−0.32187100	−0.05642200
O	−3.88819300	−0.10601900	−0.01101800
H	−4.14029700	−0.42650600	0.85839900
O	0.15947600	2.04261400	−0.00448700
H	−0.76981100	1.81196400	−0.03686100
TS17			
0	2		
C	−0.35515700	−0.41173000	−0.03030600
C	0.53715100	−1.46238800	0.04320000
C	1.89750900	−1.20643900	0.06012900
C	2.36501200	0.09972200	0.02243500
C	1.47336200	1.14911100	−0.02389900
C	0.09890600	0.91310300	−0.07074300
H	0.16237800	−2.47247000	0.07801100
H	2.59112100	−2.03106100	0.10690200
H	3.42553300	0.29532500	0.03852800
H	1.80436200	2.17516400	−0.04533600
O	−0.73334600	1.93326600	−0.20587700
H	−1.46307700	2.01777800	0.67045700
Cl	−2.04575100	−0.70763800	−0.04094400
H	−1.97647200	2.09071300	1.48960600
TS18			
0	2		
C	−0.18576900	0.66208900	−0.07117600
C	−1.40346800	1.22484100	0.25390800
C	−2.53183500	0.42825600	0.34515000
C	−2.44054200	−0.93841400	0.11711700
C	−1.22476600	−1.50457700	−0.19141400
C	−0.07729100	−0.71650700	−0.29947100
H	−1.46049100	2.28725300	0.42738400
H	−3.47974500	0.87709600	0.59598600
H	−3.31958500	−1.55914100	0.18820400
H	−1.11957300	−2.56319100	−0.36697700
O	1.04818200	−1.29661700	−0.67218200
H	1.91761200	−0.95939200	−0.20344300
Cl	1.21356700	1.64611600	−0.19952400
O	2.81913100	−1.07796000	0.79345800
H	3.07465700	−2.00411800	0.85586600

TS19			
0	2		
C	0.46659700	−0.35701600	−0.00000500
C	−0.16358300	0.88474800	0.00000900
C	−1.54014700	0.86153400	0.00001500
C	−2.29419800	−0.27838200	0.00000700
C	−1.63738100	−1.50391900	−0.00000800
C	−0.25558800	−1.53696100	−0.00001300
H	−2.25713000	2.18252500	0.00002900
H	−3.37241800	−0.22681100	0.00001100
H	−2.19759000	−2.42547100	−0.00001400
H	0.27635900	−2.47445300	−0.00002400
Cl	2.20010000	−0.39175600	−0.00001200
O	0.49340900	2.05062100	0.00001800
H	1.43834900	1.88843300	0.00001200
H	−2.69074600	2.89062500	0.00002500
TS20			
0	2		
C	0.97884700	0.16587500	−0.00001700
C	−0.25989000	−0.46354200	0.00001100
C	−1.37618000	0.34537300	0.00002200
C	−1.31363600	1.71428900	0.00002600
C	−0.06335500	2.32143100	0.00001600
C	1.07990400	1.54468500	−0.00001300
H	−2.49431400	−0.26011700	−0.00004100
H	−2.21621800	2.30455000	0.00004000
H	0.01759500	3.39661300	0.00002400
H	2.05754100	1.99857300	−0.00002900
Cl	2.39929300	−0.82385000	−0.00002200
O	−0.41880900	−1.79633300	0.00005300
H	0.43730100	−2.22877600	0.00013300
O	−3.33984900	−1.08976700	−0.00009200
H	−2.79476700	−1.88527400	0.00029000
TS21			
0	3		
C	−0.14720600	−1.18824400	−0.08678900
C	1.21199000	−1.24292700	0.00536200
C	1.88960100	−0.02673600	0.06858300
C	1.18028400	1.16767700	0.05371200
C	−0.19925400	1.17052200	−0.01792500
C	−0.90750200	−0.03424200	−0.10173500

H	1.74643000	−2.18048300	0.01563500
H	2.96732400	−0.01739100	0.12775700
H	1.71210300	2.10484300	0.10356500
H	−0.76315100	2.09118200	−0.02087900
O	−2.23034300	−0.02782900	−0.24837800
H	−2.77239100	−0.33314200	0.68016000
H	−3.21504200	−0.51869200	1.55353500
TS22			
0	3		
C	−0.08039100	0.97507800	−0.34308100
C	−1.34674500	1.42259900	−0.11904800
C	−2.31271200	0.46699200	0.19319400
C	−1.95744000	−0.87449200	0.28318300
C	−0.65599100	−1.28076000	0.07546200
C	0.33043400	−0.34419200	−0.26186300
H	−1.59990900	2.46934400	−0.18932000
H	−3.33309700	0.77269400	0.36558200
H	−2.70826800	−1.60901700	0.52856500
H	−0.36479900	−2.31670900	0.15778600
O	1.55721300	−0.76008500	−0.51647000
H	2.27817700	−0.04807800	−0.33004900
O	3.18814500	0.58015300	0.48828500
H	3.90209700	−0.02012400	0.72582900
TS23			
0	3		
C	1.76062100	−1.38197300	−0.00004100
C	0.36690300	−1.44988100	−0.00029000
C	−0.34572000	−0.28735300	−0.00026600
C	0.26482100	1.01682500	−0.00010800
C	1.70848000	1.01849000	0.00022800
C	2.42054000	−0.14482400	0.00025500
H	2.33425000	−2.29600400	−0.00006000
H	−0.13955300	−2.40240400	−0.00050300
H	2.18655900	1.98557400	0.00041700
H	3.49909400	−0.12022800	0.00049500
O	−0.38560900	2.07001900	−0.00029000
Cl	−2.23877700	−0.35470900	0.00014000
H	−3.79014500	−0.32474600	0.00092000
TS24			
0	3		
C	2.27397100	−1.46945300	0.01618500

C	0.87458300	−1.45416200	0.01819400
C	0.27318000	−0.24076300	0.01008200
C	0.92116500	1.03452600	0.00551200
C	2.36732700	0.93879100	0.00391700
C	3.00216500	−0.26913900	0.00873800
H	2.79490000	−2.41463300	0.02472100
H	0.30549200	−2.37113200	0.02950400
H	2.90780500	1.87294900	−0.00389200
H	4.08029500	−0.30929200	0.00782500
O	0.33591200	2.12415400	0.00180400
Cl	2.13482100	−0.26442200	−0.08173000
O	3.84428700	−0.16204200	0.09542300
H	4.00387500	0.78157600	0.17766400
TS25			
0	3		
C	0.53705300	−0.30666400	−0.00000200
C	−0.18688200	0.95892800	−0.00000200
C	−1.61179800	0.81268600	0.00000200
C	−2.27511400	−0.37079300	0.00000300
C	−1.51769200	−1.54916800	0.00000200
C	−0.12192200	−1.50769600	0.00000000
H	−2.36849900	2.07841300	0.00001800
H	−3.35384400	−0.41167700	0.00000600
H	−2.01515200	−2.50687400	0.00000400
H	0.44588300	−2.42407100	−0.00000100
Cl	2.23947600	−0.24361200	−0.00000600
O	0.38773900	2.04968700	−0.00000400
H	−2.82326200	2.78435500	0.00008500
TS26			
0	3		
C	−1.01007700	0.11733300	−0.00001100
C	0.28824400	−0.53436200	−0.00019600
C	1.41220800	0.35716400	−0.00028000
C	1.31397700	1.71054400	−0.00009100
C	0.03202400	2.27939200	0.00002800
C	−1.11433400	1.48391700	0.00008000
H	2.52863900	−0.25123300	0.00034200
H	2.19133000	2.33788000	0.00005500
H	−0.07431600	3.35310700	0.00017300
H	−2.09025100	1.94170100	0.00020100
Cl	−2.39377900	−0.87202500	0.00006000
O	0.42019400	−1.76299200	−0.00009000

O	3.31689800	−1.13160900	0.00028400
H	2.70985400	−1.88414900	−0.00053000
TS27			
0	3		
C	−0.30474100	−0.39569500	−0.03375900
C	0.04986400	0.96555000	−0.08093800
C	1.40731600	1.21462600	−0.03261700
C	2.40145500	0.28554200	0.02221700
C	2.00361600	−1.04909800	0.06369500
C	0.65823500	−1.38349200	0.04383900
H	3.44441800	0.56045400	0.03645600
H	2.74575200	−1.83108800	0.11118700
H	0.34939100	−2.41519800	0.08175000
Cl	−1.97221300	−0.80364600	−0.03643900
O	−0.84238500	1.92971300	−0.21336600
H	−1.53207500	2.00600400	0.68024700
H	−2.03524800	2.07950900	1.52213800
TS28			
0	3		
C	−0.21610000	0.61807500	−0.07493700
C	−0.05245800	−0.75767900	−0.32291300
C	−1.20576000	−1.51096800	−0.21086100
C	−2.44825600	−1.05287700	0.09840400
C	−2.56466800	0.31480000	0.34193200
C	−1.45230900	1.13746200	0.25774200
H	−3.30283000	−1.70842200	0.15735300
H	−3.52403700	0.73819500	0.59585500
H	−1.53928600	2.19522700	0.44435300
Cl	1.15257000	1.64812000	−0.18525600
O	1.08525500	−1.30660200	−0.69422700
H	1.93766700	−0.94411800	−0.21961700
O	2.83380800	−1.04677600	0.79247900
H	3.11960200	−1.96478100	0.84919900
TS29			
0	2		
C	2.34790500	2.52960000	−0.15273200
C	1.10604000	2.52485600	−0.77018600
C	0.50779000	1.32891200	−1.10609400
C	1.13032400	0.10487200	−0.83554800
C	2.38808600	0.13833400	−0.21308100
C	2.99245800	1.33476000	0.11800600

H	2.82396000	3.46152000	0.10909000
H	0.60874100	3.45570800	−0.99414800
H	3.96683200	1.32357700	0.57952100
O	0.57850900	−1.03475300	−1.20220400
C	−0.62099400	−1.73586400	−0.07137900
C	−1.72253500	−0.81359200	−0.14211800
C	−1.82096100	0.15046600	0.83866300
C	−0.97811500	0.14478800	1.94526800
C	−0.04850000	−0.88419100	2.11592800
C	0.07831300	−1.85586800	1.16649500
H	−1.07579900	0.92650200	2.68035400
H	0.54963400	−0.91272500	3.01277300
H	0.77928200	−2.66485100	1.28142500
H	−0.44915700	1.30218400	−1.60515000
H	−0.70675500	−2.62358900	−0.67741200
Cl	3.21858100	−1.33960800	0.08777200
Cl	−3.01015900	1.40257600	0.74939000
S	−2.69994500	−0.92623100	−1.57253900
H	−3.62772300	−0.04351200	−1.19928300
TS30			
0	2		
C	−3.31340700	−1.66087800	−0.16737100
C	−2.17127400	−2.33134100	−0.57894700
C	−1.02244200	−1.62409400	−0.86215300
C	−0.98117800	−0.23044000	−0.74480200
C	−2.15203500	0.42499100	−0.33266600
C	−3.30479500	−0.28157200	−0.05153000
H	−4.21743200	−2.20706000	0.05215900
H	−2.18152200	−3.40508600	−0.68458900
H	−4.19164100	0.25359100	0.24770800
O	0.10607700	0.44495500	−1.06693400
C	1.33400800	−1.41884800	1.44874700
C	0.52703000	−0.83420800	2.41299400
C	0.20698500	0.52743600	2.33512500
C	0.66762600	1.27961400	1.29863300
H	1.59239600	−2.46381400	1.52793000
H	0.15946200	−1.43043200	3.23253400
H	−0.38104500	0.99007600	3.11178600
H	0.46176300	2.33328000	1.22342700
H	−0.12543200	−2.12069400	−1.19972200
Cl	−2.17074900	2.14345200	−0.23907100
C	1.35589200	0.66368000	0.20756600
Cl	2.35790700	1.74711000	−0.71231100

C	1.80723900	−0.69317000	0.37648600
S	2.78192600	−1.49933900	−0.81728500
H	2.62043100	−0.58665000	−1.77818900
TS31			
0	2		
C	−3.31884000	1.42951700	−0.54037300
C	−3.35834900	0.09905100	−0.74646300
C	−2.44878900	−0.79319600	−0.04665500
C	−1.25586200	−0.12497500	0.56778700
C	−1.42837700	1.26412900	0.95171900
C	−2.37576800	2.01891400	0.36613300
C	−0.11696500	−0.11217600	−0.91319200
C	1.14641000	0.50020100	−0.52027000
C	−0.03161200	−1.48552000	−1.35893200
C	2.21729300	−0.31586000	−0.22055900
C	1.05904300	−2.24761100	−1.07990600
C	2.17894400	−1.68375600	−0.46064100
O	−2.58815200	−1.99824000	−0.03272600
H	3.03501700	−2.28615100	−0.20634200
H	−0.73649500	1.68185000	1.66395900
H	−0.88838000	−1.92314100	−1.84235800
H	−4.04294100	2.06893000	−1.02337500
H	1.07212600	−3.29229000	−1.34660500
H	−2.45274200	3.06893400	0.59956700
H	−4.11731900	−0.36751600	−1.35427200
H	−0.73370900	0.53418300	−1.53262100
Cl	−0.47307900	−1.09794400	1.78388100
Cl	3.69804000	0.33601200	0.39483900
S	1.16818100	2.23726700	−0.47457400
H	2.45165900	2.36537100	−0.13310000
TS32			
0	2		
C	2.79065500	−1.60986800	0.09225700
C	2.93357100	−0.26892800	0.06507300
C	1.92363500	0.60927000	0.65287200
C	0.61895800	−0.03201700	0.94009800
C	0.61719200	−1.46074100	1.13931300
C	1.64077200	−2.21591200	0.68788200
C	−0.31949600	0.33201300	−0.64149200
C	−1.64125800	−0.25166100	−0.47907800
C	−0.27878400	1.76913500	−0.76042300
C	−2.65264700	0.54621800	0.01525800

C	−1.31628200	2.52788600	−0.30874200
C	−2.48973700	1.92068500	0.14627400
O	2.10022800	1.80004200	0.82515000
H	−3.30278300	2.50956700	0.53759600
H	0.32018200	−0.21334600	−1.32779700
H	−0.23784400	−1.92208700	1.60583700
H	0.62880600	2.23454300	−1.10439900
H	3.57624300	−2.23524600	−0.30288000
H	0.02092500	0.55523700	1.62551000
H	−1.24186900	3.60350100	−0.30668700
H	1.61663300	−3.28960500	0.78643900
Cl	4.34830400	0.47285100	−0.55659500
Cl	−4.20343200	−0.11396100	0.41142900
S	−1.78295100	−1.93008900	−0.89652400
H	−3.07718600	−2.05908800	−0.59836600
TS33			
0	2		
C	−2.42462300	−1.52082700	−0.44653600
C	−2.51202900	−0.18995900	−0.26692200
C	−1.41769100	0.70039100	−0.64835700
C	−0.13784200	0.04731100	−1.02747700
C	−0.17922500	−1.35924000	−1.34508900
C	−1.25334700	−2.10915900	−1.02160400
C	0.98068000	0.31536900	0.47424200
C	2.26300700	−0.21949400	0.03776000
C	0.93938600	1.74677800	0.66848700
C	3.13973900	0.62891500	−0.60929600
C	1.83985200	2.55295700	0.04586200
C	2.91724500	1.99593800	−0.65400300
H	3.62155800	2.63315400	−1.16428800
H	0.67216100	−1.80816500	−1.83014400
H	0.11126800	2.15620600	1.22029700
H	−3.26013400	−2.15043600	−0.18318300
H	0.43840700	0.67310300	−1.69952700
H	1.73028600	3.62342300	0.11189800
H	−1.26319400	−3.16704100	−1.23365500
Cl	−3.92771500	0.54532200	0.35550200
Cl	0.18713600	−0.56590600	1.78532600
H	4.03963900	0.21993300	−1.04354800
S	2.69189600	−1.89901600	0.14434800
H	1.65470400	−2.28020500	0.89211900
O	−1.51951100	1.91254000	−0.62700400

TS34			
0	2		
C	3.29293800	0.69103800	0.02001500
C	2.98964800	−0.53567300	0.47466400
C	1.75126000	−1.19466500	0.09929500
C	0.74753100	−0.36981900	−0.66054500
C	1.22400800	0.87673300	−1.22615100
C	2.40682500	1.40226800	−0.85735900
C	−0.57102400	0.03909100	0.69144100
C	−1.64636600	0.75562200	0.02124600
C	−0.93323400	−1.24222600	1.25231700
C	−2.80885600	0.08710700	−0.30629100
C	−2.09347500	−1.86265100	0.91526100
C	−3.03557700	−1.21393200	0.10639000
H	−3.95812100	−1.70514700	−0.15765100
H	0.60186900	1.36470000	−1.95790400
H	−0.22342700	−1.71842300	1.90587300
H	4.23643100	1.14543800	0.28289900
H	−2.29813600	−2.84807000	1.30212500
H	2.72629800	2.34585000	−1.27190500
Cl	0.34900400	1.02210800	1.85312700
H	−3.57216100	0.61349000	−0.85937200
Cl	−0.13822600	−1.33524900	−1.81684500
H	3.66511600	−1.11147800	1.08683500
O	1.51357800	−2.34866300	0.39685600
S	−1.52446700	2.41983000	−0.47025900
H	−0.42030100	2.71170800	0.21990900
TS35			
0	2		
C	1.82175900	2.67170900	−0.47131900
C	0.69541500	2.38015900	−1.22211900
C	0.36319900	1.06359600	−1.46808600
C	1.12847900	0.01097900	−0.96432500
C	2.26684800	0.33201000	−0.21928300
C	2.61235400	1.64982400	0.02148200
H	2.09545900	3.69653000	−0.27335400
H	0.07709800	3.17310500	−1.61233000
H	3.49829700	1.86533500	0.59656500
Cl	3.29229200	−0.89868200	0.41980500
C	−0.75609300	−1.81291800	0.34046300
C	−1.75734200	−0.78448000	0.16513100
C	−1.65134100	0.36314700	0.91961600
C	−0.71140800	0.48444400	1.94038700

C	0.13186300	−0.58691700	2.24319900
C	0.07663700	−1.73419100	1.50750500
H	−0.66035200	1.40530100	2.49705200
H	0.82231400	−0.50203300	3.06722100
H	0.72680000	−2.56586400	1.72488500
H	−0.51174700	0.81600400	−2.05047100
H	−1.06972100	−2.80692200	0.04665500
Cl	−2.70388400	1.70872600	0.64914700
S	0.61132300	−1.62780800	−1.26080500
S	−2.89698300	−1.06011200	−1.12161600
H	−3.73274000	−0.06965400	−0.80556300
TS36			
0	2		
C	3.29551800	1.71340100	0.02019700
C	2.20879100	2.34237500	−0.56367800
C	1.14263200	1.58728000	−1.00771500
C	1.13012100	0.19663800	−0.88365400
C	2.24489400	−0.41486100	−0.30024900
C	3.31622500	0.33657600	0.14802200
H	4.13715500	2.29063100	0.37064200
H	2.19254000	3.41535200	−0.67325400
H	4.16236900	−0.16417100	0.58993000
Cl	2.34385000	−2.12867100	−0.14117700
C	−1.49601300	−0.61230500	0.28399100
C	−1.69893300	0.79639600	0.52360200
C	−0.97471800	1.41563000	1.51874100
C	−0.14151800	0.69544300	2.36351100
C	−0.03694300	−0.69311800	2.22928500
C	−0.74194100	−1.34370600	1.26203900
H	−1.06095500	2.48537200	1.63415200
H	0.41654700	1.21109700	3.12828300
H	0.58626000	−1.25852100	2.90375100
H	−0.68802000	−2.41338000	1.14508200
Cl	−2.87042300	−1.50726900	−0.36483400
H	0.28423700	2.06105300	−1.46000900
S	−0.25054700	−0.69841100	−1.45901600
S	−2.73881600	1.76859900	−0.48269500
H	−2.73729300	0.94204300	−1.53356300
TS37			
0	2		
C	−2.85310800	2.01875400	−0.62619400
C	−3.10664200	0.70004300	−0.77545300

C	−2.38242600	−0.29391700	−0.03227000
C	−1.06835300	0.17529500	0.50650700
C	−1.05647000	1.59171600	0.90833500
C	−1.86679800	2.47456900	0.30730100
C	0.00382200	0.04228000	−0.86102300
C	1.37311200	0.44588500	−0.48056000
C	−0.08217700	−1.31831200	−1.39388100
C	2.31399600	−0.52730900	−0.22745100
C	0.88687200	−2.23496800	−1.16087600
C	2.07456800	−1.86758500	−0.51074200
H	2.83290300	−2.59795100	−0.28364500
H	−0.32533100	1.89629900	1.63767000
H	−0.97638300	−1.59658900	−1.92475000
H	−3.45399700	2.74296500	−1.15505800
H	0.76127800	−3.25293700	−1.49415400
H	−1.80070600	3.52454000	0.54475500
H	−3.92907500	0.35535200	−1.38160700
Cl	−0.40548600	−0.84224200	1.78008800
H	−0.46068400	0.77746100	−1.51780800
Cl	3.88318900	−0.12256500	0.38186800
S	−2.92612800	−1.82823500	0.09574300
S	1.66655100	2.15577400	−0.40742100
H	2.96589700	2.07323700	−0.11396800
TS38			
0	2		
C	−2.49882400	1.88354400	0.06489100
C	−2.74746200	0.55505400	−0.03082800
C	−1.85600300	−0.40981200	0.55688300
C	−0.49169100	0.09659600	0.83420600
C	−0.40536800	1.51143800	1.18356800
C	−1.35287500	2.36835600	0.76485800
C	0.40777000	−0.12366800	−0.65811500
C	1.78864200	0.33553700	−0.43522300
C	0.30774100	−1.53360900	−1.01838900
C	2.73361900	−0.58658400	−0.04347700
C	1.28602500	−2.41116200	−0.67774800
C	2.48040300	−1.95387000	−0.10469400
H	3.24325400	−2.64440200	0.21443300
H	−0.14178800	0.53478500	−1.32694800
H	0.46266700	1.86245800	1.71656400
H	−0.60359200	−1.88097700	−1.47445900
H	−3.21525400	2.58310200	−0.33622400
H	0.04064400	−0.56681000	1.50590400

Cl	4.32256700	−0.09472900	0.44046700
H	1.15593800	−3.46684400	−0.85488600
H	−1.26939300	3.42632700	0.95678300
Cl	−4.21106900	0.02477800	−0.76081200
S	−2.24813700	−1.97135400	0.85341900
S	2.06958900	2.03004900	−0.67612100
H	3.37696300	2.01147500	−0.40765600
TS39			
0	2		
C	−2.13669500	−1.79969900	−0.48782100
C	−2.35446300	−0.49581100	−0.20170600
C	−1.40584400	0.52108700	−0.57096400
C	−0.05456600	0.04444000	−0.94905300
C	0.03126900	−1.32970500	−1.42540400
C	−0.95001900	−2.21139400	−1.16507800
C	1.02808000	0.22606500	0.47520400
C	2.35324100	−0.21318400	0.01135900
C	0.97217600	1.63586300	0.84126300
C	3.19976000	0.72163500	−0.54586500
C	1.85337600	2.52660400	0.32100900
C	2.93766600	2.08021900	−0.44852400
H	3.61996800	2.78833200	−0.89042700
H	0.91040100	−1.63124800	−1.97043400
H	0.15228500	1.95618000	1.46074100
H	−2.89159200	−2.52870900	−0.23996500
H	0.44228500	0.76676300	−1.58841400
H	1.72685300	3.57879900	0.51966100
H	−0.86864400	−3.23674300	−1.49112400
Cl	−3.83705000	−0.03757600	0.53488200
Cl	0.32886200	−0.78512400	1.75870400
H	4.11416600	0.38748800	−1.01289000
S	−1.75563100	2.12358500	−0.59841600
S	2.86689400	−1.87112800	0.00160400
H	1.80937300	−2.37098500	0.64437400
TS40			
0	2		
C	2.77290800	1.78824100	−0.00816500
C	2.87192100	0.50030300	0.38253600
C	1.92078600	−0.49456200	−0.01482600
C	0.66499100	−0.00636700	−0.65864200
C	0.72376200	1.35201800	−1.19657900
C	1.69727800	2.21000700	−0.84913100

C	−0.63743000	−0.04242700	0.64334400
C	−1.89901800	0.41568200	0.04640200
C	−0.68848700	−1.39948100	1.16435600
C	−2.86856200	−0.50172100	−0.30126300
C	−1.67190500	−2.26662400	0.81869100
C	−2.75785900	−1.83791600	0.04037800
H	−3.53626100	−2.52927400	−0.23889700
H	−0.04837800	1.63838200	−1.88999400
H	0.10704900	−1.70469700	1.82122000
H	3.53542500	2.49760700	0.27400600
H	−1.63170800	−3.28245600	1.17846800
H	1.69512500	3.21311800	−1.24710000
Cl	−0.01721400	1.06834900	1.89881400
H	−3.75666300	−0.15105300	−0.80523800
Cl	0.04642100	−1.10924200	−1.88794700
H	3.71791600	0.15368000	0.95369500
S	2.23058300	−2.08659300	0.24162200
S	−2.23798000	2.08351300	−0.30603700
H	−1.23096100	2.60621800	0.39711400
TS41			
0	2		
C	1.56123300	2.78222300	−0.00651500
C	0.52610400	2.55426900	−0.89752700
C	0.28484500	1.27160600	−1.34546800
C	1.05389500	0.18820600	−0.91794000
C	2.10343600	0.44875400	−0.03206200
C	2.35437200	1.73266100	0.41880400
H	1.76310300	3.77967000	0.35213800
H	−0.08939800	3.37025600	−1.24208300
H	3.17235200	1.90066600	1.10056000
Cl	3.14293000	−0.81222000	0.51859600
C	−0.78443300	−1.94337700	−0.08712400
C	−1.85448700	−0.99097500	−0.22069000
C	−1.95486600	0.04211400	0.67806700
C	−1.13576000	0.11514300	1.80056700
C	−0.22363300	−0.91450900	2.04953400
C	−0.09617200	−1.95530900	1.17438900
H	−1.23541700	0.94961700	2.47437500
H	0.36866900	−0.88790400	2.95026500
H	0.59908800	−2.75626700	1.36414300
O	−2.64498600	−1.13577400	−1.28429800
H	−3.28894300	−0.42465300	−1.31118300
H	−0.51335500	1.07620400	−2.04560800

H	−0.99068900	−2.90179500	−0.54665100
Cl	−3.16060500	1.25529600	0.40019100
S	0.66673500	−1.40854600	−1.50782300
TS42			
0	2		
C	3.37928200	1.31311000	−0.08636100
C	2.42747600	2.00396600	−0.81693700
C	1.27358000	1.35923000	−1.21364600
C	1.03790300	0.02079100	−0.89566800
C	2.02024700	−0.65752300	−0.16718500
C	3.17914100	−0.01699000	0.23388000
H	4.28718900	1.80339700	0.22931300
H	2.58367400	3.03906100	−1.07713300
H	3.91913600	−0.56794500	0.79146200
Cl	1.84340300	−2.32414400	0.23656900
C	−1.70108000	−0.22588000	0.21791200
C	−1.74416200	1.21527100	0.21276200
C	−1.00272200	1.92640400	1.12356600
C	−0.30112500	1.26937900	2.12451700
C	−0.37196600	−0.12469700	2.24247200
C	−1.11297100	−0.85523000	1.36245100
H	−0.99278400	3.00153000	1.04283300
H	0.28119200	1.84127600	2.82875000
H	0.14027600	−0.62500300	3.04881000
H	−1.19752900	−1.92628700	1.44092400
Cl	−3.18510800	−0.98573600	−0.38500200
O	−2.40859600	1.86731700	−0.74088500
H	−2.87600800	1.24405700	−1.30208600
H	0.52006800	1.88253500	−1.78257700
S	−0.44306800	−0.73313700	−1.42477700
TS43			
0	2		
C	−2.88337100	1.90408100	−0.80619000
C	−3.06355100	0.56476300	−0.83227600
C	−2.27258000	−0.31724500	−0.02203600
C	−0.98596300	0.27365400	0.46060000
C	−1.04015500	1.72228100	0.71993700
C	−1.90712800	2.49853900	0.05667400
C	0.08576800	0.06289300	−0.88471800
C	1.40060700	0.60115900	−0.52956400
C	0.13157400	−1.34487000	−1.28405100
C	2.46058200	−0.22767800	−0.27267200

C	1.20887000	−2.13251700	−1.03211800
C	2.38347100	−1.59971100	−0.48208600
H	3.23371200	−2.22271700	−0.26152500
O	1.47089300	1.92785000	−0.42997000
H	2.35536400	2.18298500	−0.15265300
H	−0.30684300	2.13628200	1.38995700
H	−0.74474200	−1.76527700	−1.74654200
H	−3.53433800	2.54204000	−1.38472100
H	1.17510200	−3.18165900	−1.27972500
H	−1.89127600	3.56885900	0.18700300
H	−3.87490400	0.12223400	−1.38766900
Cl	−0.26553300	−0.58364600	1.82111700
H	−0.41135800	0.70626800	−1.61126800
Cl	3.94706400	0.48735700	0.25951400
S	−2.71641500	−1.86918700	0.23544800
TS44			
0	2		
C	−2.48773400	1.85797900	−0.21833800
C	−2.65906600	0.51670000	−0.12557700
C	−1.71924700	−0.30146300	0.59170600
C	−0.39101500	0.32283200	0.79753100
C	−0.38864000	1.77780300	0.94489400
C	−1.37970800	2.50689600	0.40643600
C	0.51634600	−0.03383000	−0.64352600
C	1.86327900	0.50090600	−0.45578500
C	0.49890100	−1.47361200	−0.88896600
C	2.90670800	−0.32502300	−0.12853700
C	1.56472100	−2.26525800	−0.59230800
C	2.77445800	−1.71046800	−0.15538200
H	3.61571500	−2.33118600	0.10369800
H	−0.03818200	0.54686300	−1.37874300
O	1.98016000	1.82430900	−0.55610600
H	2.89093500	2.08218600	−0.38842200
H	0.45671000	2.25176100	1.41461300
H	−0.41444200	−1.91243500	−1.25297800
H	−3.23994600	2.44958900	−0.71618900
H	0.16561400	−0.20836600	1.56026900
Cl	4.44334000	0.39530900	0.22771300
H	1.48954100	−3.33454100	−0.71005500
H	−1.36142500	3.58449600	0.44246500
Cl	−4.08406000	−0.19616900	−0.77273300
S	−2.00672400	−1.83431200	1.09041500

TS45			
0	2		
C	−2.22853700	−1.61416400	−0.44948800
C	−2.25484700	−0.28677600	−0.18989600
C	−1.16959900	0.57555500	−0.57566000
C	0.09348400	−0.09892700	−0.95017600
C	−0.01542600	−1.48472900	−1.39148900
C	−1.11407800	−2.20897000	−1.11289000
C	1.19315300	−0.04051800	0.46270100
C	2.40681000	−0.71892500	0.00346000
C	1.38715100	1.35628200	0.80736900
C	3.44071800	0.00346200	−0.53061800
C	2.44320100	2.04745300	0.30244300
C	3.44794200	1.38929800	−0.42145200
H	4.27162600	1.95045000	−0.83238400
O	2.44715100	−2.04791800	−0.02238400
H	1.69802400	−2.40367400	0.46554800
H	0.81310500	−1.92165000	−1.92550900
H	0.62772200	1.84055200	1.39648400
H	−3.08119000	−2.22068000	−0.18923400
H	0.68427400	0.52975400	−1.60765000
H	2.51744900	3.10798200	0.48205300
H	−1.18231100	−3.24286300	−1.41420200
Cl	−3.65681400	0.39543800	0.53073700
Cl	0.38772800	−0.96309200	1.75951400
H	4.26394000	−0.53025900	−0.97766900
S	−1.27470200	2.21310000	−0.61740300
TS46			
0	2		
C	3.03476800	−1.17371400	0.12338000
C	2.81443300	0.06551700	−0.36388100
C	1.63636300	0.81669900	−0.04705100
C	0.54403400	0.08701700	0.65852800
C	0.92582900	−1.18018200	1.28554500
C	2.08498700	−1.79251300	0.99427600
C	−0.72874800	−0.28595300	−0.60072800
C	−1.74124600	−1.11324400	0.05359400
C	−1.23606300	0.95763600	−1.14702100
C	−2.94843400	−0.58882100	0.43577700
C	−2.44765000	1.44508000	−0.77329800
C	−3.30193800	0.69513000	0.05014700
H	−4.26066300	1.09464200	0.33866900
O	−1.46182900	−2.38117900	0.34879900

H	−0.66412400	−2.65441700	−0.11393800
H	0.23115700	−1.60508900	1.99144400
H	−0.60428200	1.49933500	−1.82894800
H	3.95489100	−1.68790400	−0.10717500
H	−2.76741200	2.40531100	−1.14550700
H	2.32508600	−2.73632600	1.45931200
Cl	0.15681200	−1.24612000	−1.83037700
H	−3.62579600	−1.21888800	0.98924700
Cl	−0.30894400	1.09176500	1.83232000
H	3.55517400	0.56754400	−0.96502900
S	1.51992700	2.40083400	−0.46607000
TS47			
0	2		
C	1.04840200	0.07208300	1.49330700
C	1.73069600	−0.61380600	0.46542300
C	2.75135800	0.05520500	−0.19358800
C	3.14783400	1.32713400	0.19355500
C	2.52934700	1.95436200	1.26423600
C	1.50247400	1.32032600	1.92873100
H	3.95163600	1.80740300	−0.34038100
H	2.86505000	2.93057300	1.57644900
H	1.01406200	1.79178200	2.76645500
C	−3.00188600	0.22836300	−0.34719100
C	−1.94417000	−0.06164600	0.50959100
C	−0.79230100	0.66372600	0.31937300
C	−0.63760100	1.62247300	−0.64017300
C	−1.71010500	1.89454600	−1.48353500
C	−2.89175200	1.19267400	−1.33293300
H	0.30022700	2.15070800	−0.74060500
H	−1.62555700	2.64430200	−2.25464000
H	−3.73608300	1.38253200	−1.97542000
O	−2.00751300	−0.99042400	1.47888700
H	−2.86157700	−1.42526200	1.45095100
H	0.36487900	−0.49102800	2.10962500
Cl	−4.48086100	−0.66077900	−0.15375200
Cl	3.59937300	−0.68082200	−1.50989500
S	1.14898300	−2.21985100	0.10556600
H	1.99527000	−2.47542300	−0.89136900
TS48			
0	2		
C	1.65128400	−0.13605200	0.65739900
C	1.96243100	−0.51512400	−0.67625800

C	2.66990200	0.37824000	−1.46744000
C	3.16857400	1.55935300	−0.95273500
C	2.97199900	1.87194700	0.39103400
C	2.25470200	1.02223100	1.19215900
H	2.84451100	0.12514200	−2.50189000
H	3.72959900	2.22458400	−1.58971000
H	3.40023900	2.76875100	0.81095700
H	2.11038200	1.22973300	2.24004200
C	−2.70451100	0.40578900	−0.10607500
C	−1.48213500	−0.21207100	0.14716100
C	−0.37948000	0.61198900	0.25526300
C	−0.45749800	1.97028800	0.10274100
C	−1.68758900	2.56219200	−0.15703900
C	−2.81698200	1.77396400	−0.25727400
H	0.43075400	2.57725700	0.18848100
H	−1.76372400	3.63163800	−0.27630700
H	−3.78493200	2.20519000	−0.45395700
Cl	−4.11638900	−0.59870700	−0.23300600
O	−1.37664700	−1.54660400	0.27697600
H	−2.24426300	−1.94288800	0.17141700
Cl	1.23190100	−1.35861400	1.82751000
S	1.49537000	−2.03911400	−1.38432000
H	0.49680600	−2.30275900	−0.53590600
TS49			
0	2		
C	1.08622000	−0.22440900	1.35926300
C	1.89063900	−0.64177100	0.27328000
C	2.92594000	0.18971400	−0.12591600
C	3.23019800	1.35086200	0.57087100
C	2.50008900	1.69628500	1.69867700
C	1.45356500	0.90033100	2.10797300
H	4.05041900	1.96249100	0.23191200
H	2.76605400	2.58171200	2.25409600
H	0.88055200	1.15338600	2.98561400
C	−2.95078300	0.52486300	−0.39443300
C	−1.89048100	−0.13723000	0.23070300
C	−0.68881100	0.53257000	0.23698300
C	−0.47028800	1.75704600	−0.33310900
C	−1.54143200	2.38614000	−0.95051200
C	−2.78122000	1.77291500	−0.96809100
H	0.50431200	2.22120200	−0.28749900
H	−1.41714200	3.35410800	−1.41153200
H	−3.62723400	2.25167800	−1.43344500

Cl	−4.52170600	−0.19614400	−0.45922400
H	0.39571500	−0.93393300	1.79210800
Cl	3.91303900	−0.20531200	−1.49055900
S	−1.99372900	−1.76218300	0.90528800
H	−3.24057800	−1.65744100	1.37065200
S	1.44276800	−2.15517900	−0.47247300
H	2.26879900	−2.07454900	−1.51477400
TS50			
0	2		
C	−1.71258600	−0.23498900	−0.62013600
C	−2.08463400	−0.32988900	0.75115500
C	−2.88159100	0.66732000	1.29208100
C	−3.41634600	1.67271800	0.50687200
C	−3.14126000	1.70941900	−0.85981200
C	−2.32569800	0.76111800	−1.41776100
H	−3.10176800	0.63442900	2.34808800
H	−4.05527600	2.41724600	0.95448000
H	−3.58315300	2.46915900	−1.48533600
H	−2.12358300	0.75257300	−2.47646300
Cl	−1.30471000	−1.68699100	−1.50388000
C	2.61497400	0.70924900	0.07283700
C	1.49396300	−0.09785700	−0.16363800
C	0.28457700	0.56985600	−0.27457400
C	0.15721600	1.92682200	−0.12599300
C	1.28203000	2.69279600	0.13168300
C	2.51389600	2.07929000	0.22575700
H	−0.81113200	2.39478000	−0.20153900
H	1.19944100	3.76146200	0.25568300
H	3.40749300	2.65034100	0.41681300
Cl	4.20308800	0.02599100	0.20092800
S	1.61950500	−1.85329000	−0.31408200
H	2.75685900	−1.95210200	0.37631400
S	−1.56847200	−1.62523600	1.79875100
H	−0.48507600	−1.98958800	1.09661900
TS51			
0	2		
C	1.23000100	−0.14101600	1.42886000
C	1.85129500	−0.76045200	0.32904400
C	2.90811500	−0.12203800	−0.29330100
C	3.41653900	1.07123300	0.19386500
C	2.86193000	1.63611600	1.33363300
C	1.79988000	1.02010800	1.96308400

H	4.24729900	1.53559900	−0.31197900
H	3.27901800	2.54746700	1.73174700
H	1.37370400	1.44648000	2.85713200
O	1.33676100	−1.91992000	−0.08790600
H	1.79412600	−2.21963400	−0.87593700
C	−2.81005800	0.43158500	−0.37829100
C	−1.73216600	−0.16743000	0.27946400
C	−0.57846800	0.58228400	0.33315600
C	−0.42110600	1.82363100	−0.21900800
C	−1.50958300	2.38943000	−0.86800700
C	−2.70288800	1.69471900	−0.93570600
H	0.52028700	2.34751800	−0.13697200
H	−1.43415600	3.36875500	−1.31546200
H	−3.56193900	2.12030800	−1.42811300
Cl	−4.32972600	−0.38664900	−0.51121400
H	0.54003100	−0.73149100	2.01181100
Cl	3.60874300	−0.86669500	−1.69051200
S	−1.75184400	−1.79366600	0.95753300
H	−3.04719300	−1.79915700	1.27907200
TS52			
0	2		
C	−1.82027600	−0.36983600	−0.45209100
C	−1.99941100	−0.56884200	0.94423000
C	−2.74849300	0.34265800	1.66561600
C	−3.41031600	1.37446500	1.02849900
C	−3.34442900	1.50281800	−0.35868500
C	−2.59584700	0.61838900	−1.09224900
H	−2.81416300	0.20406200	2.73273900
H	−4.00106100	2.06596900	1.60815500
H	−3.90296700	2.27704800	−0.86083200
H	−2.55931200	0.67387800	−2.16829300
Cl	−1.39385500	−1.75469100	−1.43513200
O	−1.43696200	−1.58147600	1.59498200
H	−0.75569500	−2.00687700	1.05964500
C	2.50562000	0.66764900	0.01445300
C	1.35768300	−0.12762300	−0.06160800
C	0.16922700	0.53032000	−0.31995400
C	0.08379700	1.88938000	−0.47976400
C	1.23596900	2.65398200	−0.38276500
C	2.44737700	2.04145200	−0.13372700
H	−0.86907100	2.35824200	−0.66850500
H	1.19059000	3.72576400	−0.50123200
H	3.35626400	2.61510200	−0.05646100

Cl	4.05686600	−0.03862200	0.31524700
S	1.43348100	−1.88847300	0.11403400
H	2.35883900	−1.88837700	1.07670200
TS53			
0	2		
C	−0.37489400	−0.16555600	1.38449700
C	−1.28472200	0.41620600	0.47605200
C	−2.16561800	−0.40938500	−0.20227500
C	−2.21926100	−1.76904100	0.06248600
C	−1.41068700	−2.31057500	1.05354800
C	−0.51898700	−1.51592900	1.73525400
H	−2.91436900	−2.38429800	−0.48475700
H	−1.49570600	−3.35979000	1.28986600
H	0.11422300	−1.93008700	2.50321500
C	3.59558500	0.66814900	−0.39947400
C	2.34587000	0.83672000	0.18650800
C	1.47874000	−0.23376900	0.15119300
C	1.77297400	−1.42686600	−0.45405200
C	3.01728800	−1.57819000	−1.05346100
C	3.92301300	−0.52980700	−1.00905900
H	1.05663400	−2.23616000	−0.44729500
H	3.27680900	−2.50733400	−1.53709600
H	4.89648900	−0.64181900	−1.46121900
O	2.06172600	2.02006000	0.77188100
H	1.11254800	2.17840300	0.74048300
H	0.19489300	0.48623200	2.03090900
Cl	−3.23456300	0.23670500	−1.39253300
S	−1.23021400	2.16359000	0.29318000
H	−1.30992300	2.20671800	−1.03978000
H	4.28978900	1.49448200	−0.37449500
TS54			
0	2		
C	1.03466700	0.15549000	0.68683900
C	1.65420900	−0.16512600	−0.55257400
C	2.13377600	0.86188200	−1.34283100
C	2.13590000	2.17182700	−0.89150700
C	1.69786700	2.45730100	0.39783000
C	1.19912700	1.45987900	1.19718800
H	2.54818600	0.62089600	−2.30910100
H	2.53011200	2.95414000	−1.51994100
H	1.77000800	3.46186700	0.78509700
H	0.87263000	1.66025500	2.20458000

C	−3.20444200	−0.68682900	−0.37454800
C	−1.84489700	−0.87973800	−0.14542900
C	−1.06839400	0.24636700	0.06243600
C	−1.58241400	1.51753000	0.00772500
C	−2.93705100	1.69547400	−0.23717700
C	−3.74299300	0.58362000	−0.41656100
H	−0.94592100	2.37375100	0.16724500
H	−3.35202900	2.69074400	−0.27528300
H	−4.79982700	0.70504900	−0.59796800
O	−1.40457400	−2.14825800	−0.13774900
H	−0.44656600	−2.17300700	−0.20592400
Cl	0.84406600	−1.10332100	1.88602300
S	1.71205900	−1.80790800	−1.18240200
H	2.28175800	−2.36780800	−0.11007400
H	−3.81596300	−1.56288900	−0.52895300
TS55			
0	2		
C	−0.46798400	0.24618500	1.37004400
C	−1.32037300	0.62231700	0.30704600
C	−2.34809100	−0.23674700	−0.04662300
C	−2.60063800	−1.39529100	0.67350100
C	−1.83311700	−1.69450800	1.79032100
C	−0.79527700	−0.86770700	2.15688600
H	−3.41206500	−2.03623400	0.36995000
H	−2.06191900	−2.57301600	2.37305400
H	−0.19305500	−1.08997100	3.02322300
C	3.43842600	−0.36682600	−0.73385800
C	2.42100800	0.22948500	0.00807100
C	1.26348200	−0.49123100	0.18470700
C	1.05548200	−1.73856600	−0.34389000
C	2.08000600	−2.32098300	−1.07869200
C	3.26900700	−1.63496900	−1.26378000
H	0.12250300	−2.25912200	−0.18030400
H	1.94937200	−3.30621700	−1.50062300
H	4.06821900	−2.08256300	−1.83396600
H	0.22623500	0.97758300	1.75758000
Cl	−3.37967800	0.11725300	−1.38705000
S	2.69381100	1.83437000	0.71787400
H	1.65075200	2.43071800	0.12910900
H	4.35631900	0.17762900	−0.89835400
S	−0.99243900	2.15520800	−0.47511200
H	−1.44538000	1.82767200	−1.68640500

TS56			
0	2		
C	−1.19973000	−0.22299800	−0.64107000
C	−1.57058200	−0.33940300	0.72853200
C	−2.35452800	0.65769000	1.28776300
C	−2.87836900	1.68297900	0.52114000
C	−2.61084000	1.73623100	−0.84605300
C	−1.80876700	0.78717200	−1.42259900
H	−2.57154000	0.60969500	2.34378500
H	−3.50592300	2.42842900	0.98306000
H	−3.04802000	2.50963000	−1.45813900
H	−1.61386700	0.79137600	−2.48271500
C	3.12122300	0.69314200	0.25156400
C	2.00588200	−0.07957900	−0.07084600
C	0.80510000	0.57528000	−0.27778700
C	0.70001300	1.94130600	−0.16975700
C	1.81528200	2.69490900	0.16468400
C	3.02834500	2.06634900	0.37636900
H	−0.24787000	2.42951400	−0.33175000
H	1.73012500	3.76707000	0.25766300
H	3.90569000	2.64016500	0.63125200
Cl	−0.82071500	−1.67224300	−1.54907400
H	4.06784000	0.19474600	0.39799500
S	2.26647600	−1.83794300	−0.07369200
H	1.76564600	−2.09136400	−1.28280200
S	−1.07138400	−1.66270900	1.74907200
H	0.03043900	−1.99915300	1.05819600
TS57			
0	2		
C	0.55229200	−0.34050000	1.34015000
C	1.35445600	−0.76436400	0.26607000
C	2.40796000	0.02996900	−0.14331700
C	2.74154200	1.19127200	0.53631900
C	2.00865000	1.56012100	1.65513400
C	0.94586800	0.78592000	2.07214900
H	3.57565800	1.78120500	0.19310000
H	2.28697300	2.44642500	2.20282200
H	0.38015600	1.06231300	2.94735200
O	1.00997900	−1.90811800	−0.34216700
H	1.58465800	−2.06590400	−1.09398400
C	−3.41060400	0.23532900	−0.65363700
C	−2.32862100	−0.37146300	−0.01971400
C	−1.19330900	0.38377900	0.15356900

C	−1.06161900	1.67314300	−0.29095100
C	−2.14562700	2.26459600	−0.92712500
C	−3.31816600	1.54572300	−1.09215700
H	−0.14026800	2.21721700	−0.13647100
H	−2.07482400	3.28018000	−1.28652700
H	−4.16586100	2.00048700	−1.58100300
H	−0.13861500	−1.05065800	1.76780800
Cl	3.33197700	−0.47808800	−1.51757100
S	−2.48488700	−2.04192400	0.55038200
H	−1.27350600	−2.43840400	0.15065100
H	−4.31656600	−0.33078300	−0.81275400
TS58			
0	2		
C	−1.28822000	−0.34380200	−0.48716300
C	−1.51397200	−0.53172000	0.90309300
C	−2.26649400	0.39956800	1.59454200
C	−2.87892400	1.44724100	0.93339300
C	−2.75937100	1.57222100	−0.44972400
C	−2.01101800	0.66456500	−1.15542700
H	−2.37055900	0.26692700	2.65937900
H	−3.47194400	2.15469600	1.49106200
H	−3.27741700	2.36074500	−0.97265100
H	−1.93780100	0.71451500	−2.22994000
C	3.04149300	0.57560900	0.27785300
C	1.87864800	−0.16803500	0.09349700
C	0.72809900	0.49882300	−0.27860500
C	0.71087800	1.85768600	−0.47940900
C	1.87610900	2.58830500	−0.29302300
C	3.03985600	1.94615600	0.08998700
H	−0.20195800	2.35619400	−0.76708700
H	1.86867000	3.65737100	−0.44427400
H	3.94936400	2.50694800	0.23881900
Cl	−0.88611500	−1.74858500	−1.45533000
H	3.94716100	0.06304500	0.56548900
S	1.97967400	−1.91678800	0.42625900
H	1.76811200	−2.33899100	−0.82277500
O	−0.99553100	−1.55322200	1.57675300
H	−0.26270300	−1.96081900	1.09432300
TS59			
0	3		
C	−0.86826600	1.06624600	0.83721200
C	−1.93339000	0.15151000	0.71224100

C	−2.78950600	0.30080500	−0.36890200
C	−2.65918900	1.36177600	−1.25271500
C	−1.66196200	2.30569700	−1.06616300
C	−0.78236600	2.17294900	−0.01430600
H	−3.35331500	1.44546900	−2.07307100
H	−1.58559100	3.14228600	−1.74227000
H	0.00706200	2.88809000	0.15370700
C	1.75620500	−1.85093100	−1.36582000
C	0.62592500	−1.22676300	−0.82697100
C	0.81986600	−0.16315500	−0.00760300
C	2.09398900	0.37869000	0.35074300
C	3.22766000	−0.32768200	−0.23111900
C	3.04537100	−1.40011800	−1.06140300
H	1.64047000	−2.69567900	−2.02806600
H	−0.36770700	−1.58584800	−1.05496500
H	3.90440200	−1.89902700	−1.48000500
O	2.22984400	1.36054600	1.08958700
H	−0.27683100	1.06130800	1.74049100
Cl	4.79375300	0.22439100	0.16117500
Cl	−4.08254900	−0.81615800	−0.64848600
S	−2.00000100	−1.13246600	1.89636100
H	−3.14373000	−1.67562500	1.47881700
TS60			
0	3		
C	1.64612100	−0.06906300	0.65809800
C	1.96376900	−0.56430000	−0.63716700
C	2.64841600	0.27337700	−1.50704500
C	3.12093200	1.50741100	−1.10227000
C	2.93046000	1.93126600	0.21137900
C	2.23844500	1.13867000	1.08921600
H	2.82672100	−0.06786400	−2.51511400
H	3.66158800	2.12654400	−1.80052600
H	3.34695500	2.86783000	0.54831100
H	2.10038000	1.43077800	2.11778200
C	−2.76927700	0.32957400	−0.12709500
C	−1.46687100	−0.28736900	0.09858000
C	−0.37386200	0.63895600	0.22296800
C	−0.52120000	1.98391000	0.09920500
C	−1.79280100	2.51209800	−0.15054700
C	−2.91182900	1.68401200	−0.25259200
H	0.32807300	2.64419200	0.18902500
H	−1.91644000	3.57859300	−0.26326000
H	−3.88793000	2.10436400	−0.43273500

Cl	−4.11947300	−0.70595400	−0.24245800
O	−1.34950200	−1.51582300	0.15967400
Cl	1.22859700	−1.18231000	1.93134500
S	1.54531900	−2.16287200	−1.19116500
H	0.48266100	−2.32266000	−0.38970300
TS61			
0	3		
C	2.27785500	2.55187000	−0.26256900
C	1.00904200	2.50498500	−0.83507100
C	0.50205400	1.26726700	−1.09386400
C	1.10934100	0.04620100	−0.84612600
C	2.38298900	0.15213500	−0.24982100
C	2.95874300	1.37877300	0.02259200
H	2.73848000	3.50357600	−0.04576500
H	0.46393700	3.40701500	−1.06632400
H	3.94385600	1.40983600	0.45880300
O	0.58158900	−1.11457700	−1.15748900
C	−0.62866400	−1.74598300	−0.00756200
C	−1.71992800	−0.81521700	−0.12355300
C	−1.80568400	0.19619400	0.81032000
C	−0.95721700	0.23574100	1.91201700
C	−0.03524000	−0.79230800	2.12994900
C	0.07693500	−1.81228600	1.23108300
H	−1.04561500	1.05290300	2.60876600
H	0.56869500	−0.77878200	3.02326900
H	0.77865900	−2.61589300	1.37801000
H	−0.72480600	−2.66146500	−0.56962000
Cl	3.25080400	−1.29205700	0.10608900
Cl	−2.99001300	1.44764500	0.67142400
S	−2.70566700	−1.00345900	−1.53655700
H	−3.54005300	0.00553700	−1.28439800
TS62			
0	3		
C	3.22543600	1.74291800	−0.12728300
C	2.05525500	2.39520300	−0.50942300
C	0.98572200	1.60438000	−0.80035100
C	0.93440600	0.22003700	−0.76232800
C	2.14147000	−0.38966700	−0.36335200
C	3.26411800	0.35951600	−0.06108000
H	4.11021900	2.31403800	0.10942000
H	2.00800900	3.47121200	−0.57544800
H	4.17159900	−0.14894800	0.22086500

O	−0.12587300	−0.47725000	−1.10362700
C	−1.21079700	1.28175300	1.57723300
C	−0.47250600	0.54116000	2.48711000
C	−0.25592800	−0.82718900	2.27636900
C	−0.76288000	−1.43600100	1.16980500
H	−1.37324100	2.33475800	1.74870900
H	−0.06967400	1.02396100	3.36291900
H	0.29238400	−1.40620700	3.00232400
H	−0.62752000	−2.48797100	0.98492200
Cl	2.23162700	−2.10802400	−0.31125500
C	−1.40147200	−0.66674900	0.14771900
Cl	−2.49628000	−1.57290900	−0.85447000
C	−1.72559400	0.70554500	0.43526600
S	−2.57703100	1.70200200	−0.70505300
H	−2.43658700	0.87556200	−1.74460200
TS63			
0	3		
C	−1.10028100	−0.35357200	1.38280700
C	−1.83499200	−0.69275200	0.22182900
C	−2.87726300	0.14087300	−0.15499700
C	−3.25391700	1.22538700	0.62492800
C	−2.58830100	1.49283200	1.81291700
C	−1.53858600	0.69277800	2.20525900
H	−4.07779900	1.84009300	0.30073500
H	−2.90958800	2.31770200	2.42905600
H	−1.01838800	0.88156800	3.13082200
C	1.41887300	2.33280100	−0.92562100
C	0.40740100	1.73782100	−0.18269100
C	0.68473500	0.54574100	0.42368900
C	1.90767900	−0.12577500	0.36132300
C	2.91104600	0.52254500	−0.41214700
C	2.66399100	1.72442400	−1.03794000
H	1.24480600	3.27365700	−1.42610200
H	−0.56706900	2.19522600	−0.09003600
H	3.44729000	2.18682900	−1.61565800
Cl	4.46854200	−0.17944700	−0.58469200
H	−0.41178400	−1.08641200	1.77777900
Cl	−3.78302000	−0.15536200	−1.59871600
S	2.16125800	−1.60217000	1.16264600
S	−1.28710800	−2.10756700	−0.64090000
H	−2.15003900	−2.01973000	−1.65271200
TS64			

0	3		
C	1.64815400	−0.10982200	0.66578700
C	2.17233400	−0.39871100	−0.62973600
C	2.98182000	0.54624700	−1.24221200
C	3.39769200	1.68552800	−0.57700300
C	2.99245900	1.91090700	0.73873200
C	2.15722600	1.02012500	1.35648700
H	3.31374000	0.36179800	−2.25241700
H	4.05148200	2.38481100	−1.07353200
H	3.35009800	2.77281700	1.28004800
H	1.85340700	1.15959000	2.38131400
Cl	1.20518400	−1.42449700	1.72741900
C	−2.70495200	0.61131300	−0.11312300
C	−1.51369500	−0.15850200	0.02473500
C	−0.31638500	0.57438900	0.05790800
C	−0.26208500	1.92629200	−0.14823700
C	−1.44291000	2.62674000	−0.35243300
C	−2.66440800	1.97332500	−0.31087400
H	0.68094100	2.44829200	−0.14284500
H	−1.41562600	3.69198000	−0.52610600
H	−3.58584000	2.51644900	−0.44025300
Cl	−4.24668100	−0.14579900	−0.07839700
S	−1.57653000	−1.85882300	0.03575000
S	1.83125700	−1.87272600	−1.48977900
H	0.71011700	−2.18292000	−0.81530800
TS65			
0	3		
C	1.80210500	2.67471600	−0.53653000
C	0.66844000	2.37165200	−1.28297300
C	0.41524400	1.04248800	−1.45752300
C	1.13535100	−0.01927000	−0.97087300
C	2.27371000	0.33567700	−0.23004000
C	2.60061100	1.66489700	−0.02313100
H	2.07140800	3.70525700	−0.35925400
H	0.03478700	3.14224200	−1.69219600
H	3.48344000	1.90442000	0.54657800
Cl	3.30246700	−0.87530800	0.43681600
C	−0.76073000	−1.80089200	0.33523200
C	−1.76164500	−0.77043200	0.14763900
C	−1.65645400	0.38243900	0.89496700
C	−0.71708600	0.51155500	1.91498700
C	0.12231300	−0.55990600	2.23224500
C	0.06535900	−1.71409000	1.50937700

H	−0.66681900	1.43679300	2.46447800
H	0.80835600	−0.46899500	3.05934200
H	0.71017400	−2.54669400	1.73916300
H	−1.08548900	−2.79767100	0.06156700
Cl	−2.71271200	1.72438700	0.61983700
S	0.60155800	−1.65378200	−1.25201200
S	−2.90244100	−1.06520500	−1.12938700
H	−3.69087900	−0.01889200	−0.88065900
TS66			
0	3		
C	3.82403400	−0.49711300	0.24905200
C	3.17183500	0.72566500	0.24849700
C	1.91626800	0.85538500	−0.32031600
C	1.27567400	−0.24665800	−0.91018400
C	1.99732600	−1.41370900	−0.87229800
C	3.23326300	−1.61497200	−0.32966100
H	4.80410300	−0.57448700	0.69571800
H	3.63746800	1.59214900	0.68864400
H	3.72877300	−2.57259400	−0.34746700
C	−1.53322800	−0.76918200	−0.05200500
C	−1.42076100	0.33366200	0.88259100
C	−0.56021200	0.20690800	1.95212200
C	0.04818200	−1.00436600	2.25169200
C	−0.22043400	−2.13856800	1.47297600
C	−1.02782500	−2.04508100	0.38207200
H	−0.37869800	1.07068500	2.57302500
H	0.71047400	−1.07318700	3.09966100
H	0.20483400	−3.09247200	1.74205500
H	−1.24957700	−2.89947900	−0.23596600
Cl	−3.05327000	−0.89312300	−0.93754300
S	−0.29274400	−0.21584400	−1.67234000
Cl	1.16516000	2.40133000	−0.30293800
S	−2.28602000	1.82737100	0.66113500
H	−2.32399900	1.77360600	−0.67542900
TS67			
0	3		
C	−1.14691200	−1.25124200	0.59797000
C	−1.93111400	−0.73755000	−0.45266600
C	−3.00395800	0.08302600	−0.15262200
C	−3.36793300	0.34824300	1.15830500
C	−2.64363400	−0.22216000	2.19580100
C	−1.56730100	−1.03838400	1.91831200

H	−4.21673600	0.98364100	1.35192700
H	−2.94148800	−0.03936800	3.21586000
H	−1.01229800	−1.49985700	2.71956200
O	−1.56248700	−1.03997900	−1.70007200
H	−2.14708500	−0.61192200	−2.32847600
C	1.28412400	2.34846000	0.51785200
C	0.27850300	1.40009100	0.65031300
C	0.60335900	0.09243900	0.42341300
C	1.87467000	−0.37161300	0.07642700
C	2.86962700	0.63787400	−0.05420400
C	2.57289300	1.96513200	0.16410700
H	1.07281300	3.39367400	0.68802700
H	−0.72767600	1.68280600	0.92504400
H	3.35208100	2.70212100	0.05942500
Cl	4.47941300	0.22509100	−0.48733600
H	−0.44789900	−2.03860400	0.36045700
Cl	−3.91762800	0.75241000	−1.46327600
S	2.19274300	−2.02475400	−0.15555500
TS68			
0	3		
C	1.76961900	−0.27635200	0.52190100
C	2.10762800	−0.71409700	−0.79066000
C	2.94296200	0.06666800	−1.56948800
C	3.53474800	1.20239400	−1.05149900
C	3.31212200	1.57054200	0.27565400
C	2.48026700	0.81767400	1.06278500
H	3.13140700	−0.25715200	−2.58037100
H	4.19445800	1.78868000	−1.67143800
H	3.81881900	2.42536300	0.69529400
H	2.32686600	1.05574900	2.10314800
Cl	1.25406400	−1.48121700	1.68168500
O	1.61996000	−1.83485700	−1.30377200
H	0.82954500	−2.11733000	−0.81662500
C	−2.58049600	0.58788800	−0.07214300
C	−1.37258800	−0.16554600	−0.07794500
C	−0.18950000	0.56960500	0.09830500
C	−0.16272400	1.93324700	0.20198700
C	−1.36144400	2.63344400	0.15454200
C	−2.56805600	1.96082900	0.03304100
H	0.77179500	2.45758800	0.32607600
H	−1.36087000	3.71089100	0.22584100
H	−3.50032400	2.50062300	0.01587800
Cl	−4.10277400	−0.19355700	−0.21961800

S	−1.38865800	−1.84576000	−0.34010400
TS69			
0	3		
C	1.51460300	2.79568800	−0.09612900
C	0.46078000	2.53737400	−0.96636600
C	0.30397000	1.23165400	−1.32926200
C	1.05091400	0.15247200	−0.92981700
C	2.10650600	0.46343400	−0.05885000
C	2.33236300	1.76825300	0.34630100
H	1.70646600	3.80604500	0.23300200
H	−0.18479400	3.32257600	−1.32627100
H	3.15337600	1.97485600	1.01307200
Cl	3.16284600	−0.76803500	0.52075300
C	−0.77848500	−1.93935200	−0.06479400
C	−1.85303400	−0.98922000	−0.21692800
C	−1.94936700	0.06534800	0.65631900
C	−1.12792400	0.16337700	1.77609600
C	−0.21974800	−0.86439500	2.05170700
C	−0.09387600	−1.92542000	1.20276700
H	−1.22582900	1.01382800	2.42988800
H	0.36921500	−0.81869600	2.95396200
H	0.59655600	−2.72529400	1.41375300
O	−2.64755500	−1.16778000	−1.27001400
H	−3.25999700	−0.43289700	−1.34727900
H	−0.99851100	−2.91151600	−0.48922500
Cl	−3.15430100	1.27268300	0.35425300
S	0.64990400	−1.45131000	−1.48888500
TS70			
0	3		
C	3.60015100	−0.84544800	0.03317800
C	3.09851000	0.42075900	0.28919400
C	1.85561000	0.79703100	−0.19048900
C	1.07831400	−0.09266800	−0.94983400
C	1.65427100	−1.32025600	−1.15992600
C	2.86813800	−1.75934900	−0.71678600
H	4.57286600	−1.11687700	0.41547200
H	3.67244600	1.12859700	0.86435700
H	3.24479300	−2.74671800	−0.93161000
C	−1.72050700	−0.42214300	−0.06599900
C	−1.40409300	0.42405300	1.06352400
C	−0.57346300	−0.04297600	2.05368700
C	−0.17242200	−1.37046800	2.06523200

C	−0.63571900	−2.26110700	1.08439700
C	−1.44507600	−1.82344700	0.08165600
H	−0.26940100	0.64546800	2.82558800
H	0.47496600	−1.72377600	2.85151300
H	−0.36750600	−3.30437400	1.13573800
H	−1.82156900	−2.48749700	−0.67868000
Cl	−3.28696700	−0.05098100	−0.81422400
O	−1.81793500	1.68616200	1.09847700
H	−2.35203500	1.88402800	0.32588500
S	−0.48956600	0.27311800	−1.62149100
Cl	1.28694900	2.38263200	0.15662200
TS71			
0	3		
C	0.41542100	−0.18143300	1.47682900
C	1.18396300	0.50419700	0.51002200
C	2.13614500	−0.22006900	−0.19271100
C	2.38918600	−1.55277600	0.09677000
C	1.69489100	−2.18847800	1.11453600
C	0.73292800	−1.49970400	1.82030800
H	3.14542800	−2.07450800	−0.46685900
H	1.92387400	−3.21419400	1.35688100
H	0.18802900	−1.97614400	2.61967700
C	−2.57007900	−1.28059700	−1.65010800
C	−1.46933100	−1.32306900	−0.79220000
C	−1.44679800	−0.47053800	0.26887500
C	−2.48596900	0.46548800	0.57082700
C	−3.59792600	0.47029400	−0.35122100
C	−3.62401000	−0.37872300	−1.41874800
H	−2.61223800	−1.94175700	−2.50276300
H	−0.65159400	−2.00811300	−0.97092300
H	−4.46138800	−0.36418700	−2.09928900
O	−2.44825200	1.22346100	1.55579100
H	−0.22656400	0.40216000	2.12093600
Cl	3.07763900	0.51587300	−1.44433500
S	0.78244600	2.18521300	0.26080800
H	1.68779900	2.41979700	−0.68901300
H	−4.39686000	1.16845600	−0.15327700
TS72			
0	3		
C	1.07057700	0.12313400	0.68521800
C	1.57972800	−0.22281300	−0.59787500
C	2.05250200	0.79838600	−1.40954200

C	2.14897200	2.10018200	−0.95482100
C	1.78425600	2.41010200	0.35360100
C	1.29077300	1.42946300	1.17439800
H	2.36847200	0.55286700	−2.41166300
H	2.53510500	2.86673000	−1.60788500
H	1.91275300	3.41181500	0.73342300
H	1.02680700	1.63697100	2.19895800
C	−3.22377600	−0.68548600	−0.38124900
C	−1.82538800	−0.90470600	−0.09098100
C	−1.04176400	0.27489900	0.15718300
C	−1.57264400	1.52862000	0.09662100
C	−2.92572300	1.68755200	−0.21279500
C	−3.74518100	0.57225500	−0.44259900
H	−0.95809400	2.39680000	0.28246800
H	−3.34479700	2.68079800	−0.27275100
H	−4.78982500	0.71756000	−0.67025400
O	−1.36616400	−2.06015600	−0.07736100
Cl	0.90531600	−1.10890400	1.90684000
S	1.65404000	−1.85750900	−1.20255900
H	0.59219600	−2.31327200	−0.51910600
H	−3.82229700	−1.56703300	−0.55259200
TS73			
0	3		
C	−3.15158600	−1.46372500	0.10564100
C	−2.07346300	−1.84193700	−0.69370200
C	−1.25119700	−0.85025000	−1.13919500
C	−1.39662800	0.50494200	−0.87854400
C	−2.48212300	0.84523300	−0.05482800
C	−3.34789900	−0.12475300	0.41470500
H	−3.83332800	−2.21380500	0.47729200
H	−1.90581000	−2.87774700	−0.94764600
H	−4.18603500	0.16385500	1.03021900
O	−0.59936900	1.42950200	−1.38121800
C	0.90876800	1.72115800	−0.46322000
C	1.47904400	0.41380700	−0.29164100
C	1.25212300	−0.23951100	0.90252600
C	0.61780400	0.39560500	1.96372800
C	0.24035500	1.73776300	1.85820200
C	0.44460500	2.41539600	0.69280400
H	0.44779000	−0.15458200	2.87434400
H	−0.19404900	2.23097600	2.71336100
H	0.16881000	3.45208700	0.58992900
H	1.31449000	2.31421400	−1.26734200

Cl	1.76556200	−1.87217700	1.13984400
S	2.22298600	−0.25502500	−1.70717600
H	2.58852000	−1.41540800	−1.16158900
H	−2.62657800	1.88944200	0.17979200
TS74			
0	3		
C	3.80105400	0.46360700	−0.09880500
C	2.88132100	1.51076000	−0.09737600
C	1.60867400	1.22164700	−0.49338800
C	1.15083100	−0.01929300	−0.91045100
C	2.10095900	−1.05292500	−0.88129000
C	3.40355600	−0.80765500	−0.49158700
H	4.82219900	0.64575700	0.20047600
H	3.17258800	2.50666600	0.20077600
H	4.12074000	−1.61391500	−0.49608000
O	−0.07624800	−0.23167800	−1.34996700
C	−0.56434800	0.50047800	1.90792400
C	−0.03515000	−0.69623900	2.36309200
C	−0.21450000	−1.87466000	1.62501700
C	−0.89793100	−1.84675900	0.44852800
H	−0.42089200	1.40366700	2.48072200
H	0.51243900	−0.71773400	3.29151800
H	0.16732100	−2.81028200	2.00209400
H	−1.07127900	−2.73746400	−0.13225800
C	−1.32687900	−0.60431200	−0.10954400
Cl	−2.64967600	−0.73693000	−1.23214500
C	−1.25526400	0.56991000	0.71570300
S	−1.84039800	2.11069800	0.16606200
H	−1.90073200	1.78176000	−1.12722600
H	1.78452400	−2.03575100	−1.19776100
TS75			
0	3		
C	0.42560300	0.55055200	1.23343000
C	1.34208200	0.69295800	0.16666600
C	2.38001800	−0.22178500	0.07371600
C	2.56907500	−1.20147100	1.03900500
C	1.71372100	−1.27830100	2.12817700
C	0.66732600	−0.39017200	2.24173400
H	3.39598500	−1.88468200	0.93365300
H	1.88560200	−2.02365300	2.88847600
H	0.00117600	−0.42912800	3.08885900
C	−1.99595700	−2.28835500	−1.06446200

C	−0.99543500	−1.62555900	−0.36434500
C	−1.28328000	−0.39585500	0.16204300
C	−2.52237500	0.23396400	0.04425200
C	−3.51867900	−0.46983800	−0.67421500
C	−3.25447300	−1.70527600	−1.21496400
H	−1.79911100	−3.25948300	−1.49400200
H	−0.01601100	−2.06530700	−0.23762000
H	−4.02473500	−2.22825200	−1.75999200
H	−0.27163400	1.35483400	1.41682400
Cl	3.51185100	−0.15985300	−1.23375200
S	−2.85088200	1.76810800	0.70922000
H	−4.48838800	−0.00905600	−0.78526600
S	1.00306100	1.98855000	−0.95396700
H	2.01502500	1.75053400	−1.78742600
TS76			
0	3		
C	1.14293100	−0.04463800	0.67732900
C	1.68651100	−0.33015000	−0.60852100
C	2.44134600	0.64779400	−1.23815600
C	2.78079400	1.82615100	−0.59774400
C	2.35658200	2.05455500	0.71033700
C	1.57782400	1.12502200	1.34676200
H	2.78914600	0.46132100	−2.24261500
H	3.39172600	2.55376700	−1.10796400
H	2.65638700	2.94947700	1.23297400
H	1.26114500	1.26762200	2.36726100
Cl	0.73349300	−1.35986000	1.75120100
C	−3.24553000	0.35305200	−0.20542100
C	−2.00005100	−0.29379000	−0.02398000
C	−0.86432200	0.52450100	0.05261500
C	−0.93433800	1.88120700	−0.13089600
C	−2.16612900	2.47955800	−0.36781600
C	−3.32580800	1.71290100	−0.38514500
H	−0.04183600	2.48563000	−0.08540100
H	−2.21992300	3.54682500	−0.52305600
H	−4.28435900	2.18108800	−0.54503400
S	−1.99485800	−2.00162500	−0.01364800
H	−4.13098600	−0.26368100	−0.22783900
S	1.43822000	−1.84146300	−1.43835200
H	0.31667400	−2.19200500	−0.78292500
TS77			
0	3		

C	3.00955800	1.59192000	−0.37361700
C	1.95667400	1.60114600	−1.28436000
C	1.24616700	0.44090600	−1.40214600
C	1.46291500	−0.72008300	−0.70052200
C	2.53047500	−0.69771100	0.20495900
C	3.29385000	0.44607800	0.35678500
H	3.61067500	2.47942300	−0.24220500
H	1.72230100	2.48139000	−1.86264000
H	4.11696200	0.44726800	1.05452100
C	−1.09830200	−1.63676300	0.42411800
C	−1.58279100	−0.32436300	0.05645800
C	−1.14783100	0.75907200	0.79076300
C	−0.38508200	0.60214200	1.94364600
C	−0.08266500	−0.67856100	2.41339100
C	−0.48384400	−1.77794400	1.71416300
H	−0.05940700	1.47892800	2.47802700
H	0.45026700	−0.78754400	3.34468100
H	−0.27277500	−2.77353100	2.06923600
H	−1.72707100	−2.45839200	0.10457100
Cl	−1.56197200	2.37436500	0.33037300
S	0.45869000	−2.12938100	−0.92149800
H	2.74516800	−1.58879900	0.77602900
S	−2.55440100	−0.26556900	−1.38211100
H	−2.81596300	1.04120000	−1.34263000
TS78			
0	3		
C	3.86321500	0.33938400	0.10568900
C	3.02385300	1.41936800	−0.13495100
C	1.77228500	1.23196700	−0.69135600
C	1.33162700	−0.05564700	−1.02428400
C	2.21101300	−1.07812000	−0.76435800
C	3.45644000	−0.95203800	−0.21555900
H	4.83983500	0.49980100	0.53803400
H	3.35147200	2.41672100	0.11437200
H	4.09829400	−1.80168200	−0.04084200
C	−1.40169300	−0.59165100	0.05682400
C	−1.16513500	0.61209100	0.81551400
C	−0.28398700	0.57193100	1.87500800
C	0.28398300	−0.62017100	2.29916900
C	−0.04462500	−1.82091500	1.65912700
C	−0.90822200	−1.82247800	0.60622800
H	−0.03603300	1.49361200	2.37893500
H	0.97139900	−0.61780900	3.12953800

H	0.36786200	−2.75307700	2.01118200
H	−1.19089900	−2.73398000	0.10611200
Cl	−2.98297800	−0.74714300	−0.71019200
H	1.11338000	2.06898100	−0.87364600
S	−0.23937500	−0.33800500	−1.72700000
S	−1.85511200	2.14543900	0.35790700
H	−2.12542400	1.80758000	−0.90724600
TS79			
0	3		
C	−0.48943700	−1.18500700	0.69049200
C	−1.35724500	−0.79381900	−0.34609600
C	−2.43251000	0.02491500	−0.04997900
C	−2.71623700	0.40603800	1.25208100
C	−1.90682000	−0.04485100	2.28484600
C	−0.82672600	−0.85796400	2.01052200
H	−3.56901600	1.03666300	1.44379000
H	−2.14012900	0.22774100	3.30172700
H	−0.20459500	−1.22594500	2.81082700
O	−1.06431100	−1.20429200	−1.58320200
H	−1.70179300	−0.84915700	−2.20543000
C	1.85002500	2.46050000	0.11169400
C	0.87947000	1.51067400	0.40472200
C	1.20508500	0.18826100	0.26842700
C	2.45973500	−0.26651800	−0.13816300
C	3.42358900	0.72717700	−0.43367200
C	3.11860700	2.06189800	−0.30915900
H	1.62247200	3.51168500	0.20950200
H	−0.10747700	1.80470000	0.73403700
H	3.86606500	2.80584200	−0.53666500
H	0.22158900	−1.96896400	0.47744700
Cl	−3.45299200	0.54304100	−1.35115100
S	2.84874900	−1.92094400	−0.26992400
H	4.40302800	0.40736500	−0.75556000
TS80			
0	3		
C	1.24393400	−0.21357100	0.54889000
C	1.64620900	−0.63448000	−0.74956000
C	2.45592700	0.19256600	−1.50658200
C	2.95635100	1.36789700	−0.98000200
C	2.66557800	1.73024100	0.33488800
C	1.86079100	0.92794500	1.10261100
H	2.69661200	−0.12419900	−2.50861400

H	3.59684800	1.99169400	−1.58329800
H	3.09944600	2.62021600	0.76302800
H	1.65897200	1.16174200	2.13570000
Cl	0.75741900	−1.44397100	1.69494500
O	1.24467100	−1.78549800	−1.27300400
H	0.45056300	−2.11225100	−0.82062100
C	−3.13813200	0.32397800	−0.25882900
C	−1.87596600	−0.31168400	−0.19182700
C	−0.76062500	0.49908000	0.05773500
C	−0.86516800	1.85948300	0.18433800
C	−2.11473900	2.46016500	0.07975000
C	−3.25249200	1.68701700	−0.13026200
H	0.01203700	2.46092500	0.36764700
H	−2.20125700	3.53260900	0.17258800
H	−4.22165400	2.15599200	−0.19554400
S	−1.80301800	−1.99334400	−0.46738900
H	−4.00676800	−0.29355600	−0.42958200
TS81			
0	3		
C	2.88223600	1.76628800	0.02590600
C	1.90918800	1.82718100	−0.96774600
C	1.26053600	0.65942700	−1.25445900
C	1.47020600	−0.55729300	−0.65319000
C	2.45732900	−0.58435200	0.33873200
C	3.15344600	0.56526900	0.66672500
H	3.43214400	2.65729200	0.29060100
H	1.68780100	2.75023200	−1.48082600
H	3.91451100	0.52717100	1.43067900
C	−1.09048000	−1.76035100	0.17310300
C	−1.74044700	−0.54462400	−0.24013800
C	−1.54383900	0.60450600	0.48567700
C	−0.85555800	0.59381600	1.69472800
C	−0.39756300	−0.62197100	2.21180400
C	−0.56914300	−1.78204800	1.51189000
H	−0.71160100	1.51773400	2.22944100
H	0.07423500	−0.63612000	3.18148700
H	−0.23162100	−2.72544900	1.90948900
O	−2.44103000	−0.60617200	−1.37014200
H	−2.74042100	0.27134400	−1.61807600
H	−1.58056900	−2.65885500	−0.18007500
Cl	−2.21385000	2.08307500	−0.11854200
S	0.56502400	−1.98386800	−1.09892800
H	2.66335000	−1.51942900	0.83828500

TS82			
0	3		
C	−3.77879900	0.06757700	0.11698500
C	−3.05298400	−0.36544600	1.21878700
C	−1.78197700	−0.88780600	1.06647800
C	−1.20716400	−0.99189400	−0.20625900
C	−1.97706100	−0.54669000	−1.25232700
C	−3.23568800	−0.02122200	−1.16110900
H	−4.77215200	0.47051200	0.24840900
H	−3.48499400	−0.29448500	2.20493800
H	−3.78757600	0.30650300	−2.02854900
C	1.54677500	0.12762100	−0.27697700
C	1.23038000	0.64090700	1.03133100
C	0.37417400	1.70667500	1.16133600
C	−0.09824400	2.36901500	0.03797400
C	0.31850000	1.98092600	−1.24248600
C	1.17659100	0.93437900	−1.40069100
H	0.09205900	2.01437400	2.15537000
H	−0.77235600	3.20191800	0.15567400
H	−0.01804800	2.52881200	−2.10810500
H	1.52923100	0.62803400	−2.37158000
Cl	3.14837200	−0.62615600	−0.38617800
O	1.66856700	0.01789300	2.12491500
H	2.23584100	−0.71682700	1.87987800
H	−1.21305100	−1.22374400	1.92165700
S	0.38957300	−1.65611600	−0.44064000
TS83			
0	2		
C	2.01652000	2.59029800	0.30984400
C	0.84044600	2.61509700	−0.42471300
C	0.35539700	1.45302100	−0.98577100
C	1.02650800	0.23497000	−0.83302100
C	2.21601800	0.23883600	−0.08876900
C	2.70787000	1.40212700	0.47050700
H	2.40494700	3.49538700	0.74982600
H	0.30727300	3.54276400	−0.56323000
H	3.63300400	1.37066900	1.02344300
O	0.58055400	−0.86110600	−1.41638900
C	−0.66356700	−1.78448700	−0.52070300
C	−1.79007200	−0.90227600	−0.54946100
C	−2.05123400	−0.11369200	0.54637500
C	−1.32703100	−0.25239000	1.72296200

C	−0.35462100	−1.25254300	1.82135000
C	−0.08321200	−2.05972700	0.75270300
H	−1.54359400	0.39586900	2.55589000
H	0.16003000	−1.39566200	2.75793400
H	0.64998700	−2.84499900	0.82079500
H	−0.54702900	1.45182900	−1.57824500
H	−0.66519400	−2.56009600	−1.26869500
Cl	3.10955100	−1.22341400	0.08019400
Cl	−3.31960200	1.05925100	0.43248600
O	−2.45920900	−0.82740200	−1.69775500
H	−3.15744800	−0.17232300	−1.62796300
TS84			
0	2		
C	−3.37414000	−1.23646300	−0.21189400
C	−2.37501100	−1.98581600	−0.81456600
C	−1.15357000	−1.40861000	−1.08941800
C	−0.89578700	−0.07096400	−0.77492200
C	−1.92427800	0.66793800	−0.17209500
C	−3.15004000	0.09231700	0.10240300
H	−4.33366500	−1.67935400	0.00450600
H	−2.55309700	−3.01774100	−1.07438000
H	−3.92413000	0.69187200	0.55387900
O	0.26187500	0.48427500	−1.08895900
C	1.36415100	−1.93490600	0.98613000
C	0.72792500	−1.40997500	2.10038500
C	0.61098900	−0.02269900	2.27032500
C	1.10564900	0.83092200	1.33025700
H	1.49087800	−2.99790100	0.85884500
H	0.34001600	−2.07723300	2.85310300
H	0.15843100	0.37262400	3.16561000
H	1.06070700	1.89974300	1.44636200
H	−0.36508100	−1.96683400	−1.57079300
Cl	−1.67943100	2.33681200	0.17366700
C	1.59739600	0.31566600	0.09719900
Cl	2.71775400	1.32449200	−0.77855900
C	1.84738600	−1.09760500	0.01048400
O	2.42702600	−1.60970500	−1.07203500
H	2.67921900	−0.90272500	−1.67173500
TS85			
0	2		
C	3.32027500	−1.30609700	−0.74470000
C	3.29520800	0.04118900	−0.75229400

C	2.33175300	0.77635400	0.04829600
C	1.17585500	−0.03568400	0.54451100
C	1.40469200	−1.45859100	0.71363100
C	2.39571500	−2.07033900	0.04041900
C	0.05261300	0.11358200	−0.93819600
C	−1.15580200	−0.60747300	−0.60734200
C	−0.13279300	1.52141700	−1.20680900
C	−2.30475900	0.06893400	−0.28112200
C	−1.29150500	2.16140400	−0.88242900
C	−2.39071400	1.45058300	−0.39059200
O	2.39770600	1.97536100	0.22771200
H	−3.30716500	1.94968700	−0.12363300
H	0.71509600	−2.01310200	1.32740300
H	0.69797600	2.08111100	−1.60176500
H	4.08305600	−1.83001600	−1.30175300
H	−1.37498200	3.22687000	−1.02510600
H	2.51778800	−3.13950700	0.10815300
H	4.03793600	0.62771300	−1.26950100
H	0.69373800	−0.42939900	−1.62743600
Cl	0.32718500	0.71299600	1.87182000
Cl	−3.68585300	−0.85391400	0.21197700
O	−1.05669400	−1.93679400	−0.58444000
H	−1.89742800	−2.31797900	−0.31732500
TS86			
0	2		
C	−2.74934800	1.60603900	−0.18213400
C	−2.83548800	0.27191400	0.00018300
C	−1.78471300	−0.46404600	0.69711200
C	−0.51277600	0.27321700	0.87601400
C	−0.56698400	1.71513400	0.84682400
C	−1.62240800	2.34625400	0.29253900
C	0.42929700	−0.26704200	−0.64803400
C	1.71413700	0.38145200	−0.54539200
C	0.46618800	−1.70852500	−0.61358300
C	2.80046000	−0.31216800	−0.06931500
C	1.56815100	−2.37352700	−0.15812700
C	2.73991800	−1.68189700	0.15755300
O	−1.89926800	−1.62650300	1.03799500
H	3.61079700	−2.19656800	0.52817200
H	−0.22080000	0.18883500	−1.38740200
H	0.27742700	2.27722000	1.21059700
H	−0.43028000	−2.25166400	−0.85911400
H	−3.56475300	2.12837300	−0.65836300

H	0.10834000	−0.17983600	1.63782400
H	1.54592500	−3.44651100	−0.05519500
H	−1.64205700	3.42153100	0.21365300
Cl	−4.22518100	−0.61205700	−0.47810200
Cl	4.28587600	0.54829100	0.16833600
O	1.74568700	1.67667500	−0.85754800
H	2.63362600	2.02043700	−0.72956200
TS87			
0	2		
C	2.43659000	1.36740400	−0.39330500
C	2.36332300	0.02819100	−0.27234300
C	1.16690000	−0.70788100	−0.67163600
C	−0.02172700	0.11092700	−1.01621600
C	0.18030800	1.51910900	−1.26178000
C	1.33873900	2.12024600	−0.91837400
C	−1.15417300	−0.09468700	0.47920300
C	−2.31404400	0.68787700	0.09912300
C	−1.36293900	−1.51475400	0.58938900
C	−3.35908100	0.08308500	−0.55278300
C	−2.42314600	−2.09193100	−0.04297000
C	−3.40178400	−1.30101500	−0.65761800
H	−4.23204700	−1.76853600	−1.16229900
H	−0.62053900	2.08855400	−1.70679800
H	−0.60553300	−2.10566000	1.07435700
H	3.34548000	1.87793000	−0.11560900
H	−0.67064600	−0.40891400	−1.71152600
H	−2.51732000	−3.16585800	−0.04801800
H	1.47235600	3.17910500	−1.07681700
Cl	3.68779900	−0.89650400	0.29706300
Cl	−0.25424900	0.62635700	1.82522200
H	−4.15665100	0.70150100	−0.93197400
O	1.11379200	−1.92406500	−0.68627000
O	−2.30407800	2.00999700	0.25081900
H	−1.55696200	2.26748800	0.79932500
TS88			
0	2		
C	3.26949100	0.41223000	−0.14983100
C	2.82979500	−0.60422300	0.61172500
C	1.51001900	−1.17903000	0.42195500
C	0.61267900	−0.49189000	−0.56675100
C	1.21264000	0.51294400	−1.42188900
C	2.45397700	0.97743300	−1.18712900

C	−0.64993300	0.40130200	0.58268200
C	−1.49286100	1.16697500	−0.31259400
C	−1.30983200	−0.62545500	1.34315700
C	−2.75281100	0.72972300	−0.63569500
C	−2.56780000	−1.02910500	1.01454300
C	−3.29225200	−0.37117100	0.01035600
H	−4.28937400	−0.69830800	−0.23668000
H	0.62617100	0.87667600	−2.25083300
H	−0.75354400	−1.10693900	2.12890700
H	4.26634800	0.80121400	−0.00590100
H	−3.01705000	−1.84786500	1.55343400
H	2.86878000	1.74552800	−1.82141500
Cl	0.40604100	1.45659800	1.55661400
H	−3.31783000	1.29077300	−1.36247800
Cl	−0.37291200	−1.62472900	−1.46116100
H	3.44528700	−1.07183200	1.36352500
O	1.12345700	−2.15040500	1.04359700
O	−1.01681900	2.26955400	−0.89049500
H	−0.18377400	2.51738400	−0.47926200
TS89			
0	2		
C	1.00453100	−1.36311100	−0.29943800
C	1.93962700	−0.44010300	−0.79406500
C	2.94218200	0.01013600	0.04662500
C	3.08280800	−0.48318500	1.33371900
C	2.20944700	−1.45796400	1.79114900
C	1.19872100	−1.91537800	0.97117800
H	3.88066600	−0.11136700	1.95584700
H	2.33363300	−1.86093200	2.78340600
H	0.51816500	−2.67811800	1.31421000
C	−3.03537500	0.40699500	0.08122400
C	−1.96123400	−0.42654500	−0.21291600
C	−0.71276200	0.04620500	0.11342300
C	−0.48478100	1.25263400	0.71162100
C	−1.57528200	2.06830100	0.99824700
C	−2.85078900	1.64104400	0.67906500
H	0.52119500	1.56567100	0.95335000
H	−1.43239400	3.03001500	1.46596400
H	−3.71122000	2.25513900	0.88964700
Cl	−4.63425200	−0.14004500	−0.32065200
H	0.32666600	−1.82136600	−1.00202400
Cl	4.05187400	1.19521700	−0.56094800
O	1.78367500	−0.01090100	−2.05020900

H	2.45857000	0.63706000	−2.26042100
O	−2.09772900	−1.63713400	−0.78358500
H	−3.02497900	−1.80392000	−0.96142200
TS90			
0	2		
C	−1.75574000	−0.38180800	−0.48480300
C	−1.88414900	−0.70426500	0.88996600
C	−2.60150700	0.14161900	1.71576100
C	−3.25283800	1.24433600	1.19769400
C	−3.21426300	1.51041100	−0.16950400
C	−2.51340800	0.68010500	−1.00837200
H	−2.64097900	−0.09565400	2.76666600
H	−3.81243200	1.88888100	1.85702500
H	−3.76078800	2.34641200	−0.57661900
H	−2.50150200	0.83807800	−2.07476800
C	2.58425900	0.38844000	0.06166800
C	1.35551800	−0.25802600	−0.01025200
C	0.25403000	0.51755000	−0.29780700
C	0.33100900	1.86948800	−0.49293500
C	1.56906900	2.49737000	−0.41333800
C	2.69865200	1.75170600	−0.13713900
H	−0.56164800	2.43845300	−0.70725100
H	1.65212100	3.56201800	−0.56692900
H	3.67006400	2.21377400	−0.07059400
Cl	3.99350100	−0.56239100	0.41788200
O	1.22892000	−1.58610700	0.19480900
H	2.08797500	−1.96429900	0.39425700
Cl	−1.30896000	−1.65890800	−1.58782400
O	−1.30256200	−1.77681000	1.42012900
H	−0.60465800	−2.10379600	0.84209800
TS91			
0	3		
C	−0.97612800	1.20131900	0.68928500
C	−1.99993900	0.25604700	0.85454800
C	−2.92367700	0.08920400	−0.16198700
C	−2.90419100	0.88776700	−1.29368100
C	−1.94437700	1.88144400	−1.41432100
C	−1.00452500	2.05504500	−0.41972100
H	−3.64672100	0.73420600	−2.05985100
H	−1.94545600	2.52054500	−2.28265900
H	−0.24891600	2.82097600	−0.49307200
C	1.63114000	−2.14026000	−0.81336600

C	0.50254600	−1.41235900	−0.42183700
C	0.69162600	−0.17952000	0.11412900
C	1.96621500	0.44266700	0.30218900
C	3.09953700	−0.37362000	−0.11257600
C	2.91881500	−1.61799400	−0.65252500
H	1.51533600	−3.12142700	−1.24883200
H	−0.48852600	−1.82488800	−0.54696700
H	3.77769900	−2.19571900	−0.95378300
O	2.10330600	1.57751100	0.77320500
H	−0.35408700	1.43777300	1.53824800
Cl	4.66495400	0.27248100	0.09893100
Cl	−4.14140500	−1.13078800	0.02516600
O	−1.99303100	−0.48111500	1.97077300
H	−2.73411200	−1.08985400	1.96463500
TS92			
0	3		
C	1.74167900	−0.32318000	0.52175700
C	1.90382200	−0.80452200	−0.80450200
C	2.67658200	−0.07300300	−1.69014600
C	3.33604900	1.06861000	−1.27796600
C	3.25121200	1.49607700	0.04597800
C	2.50173200	0.78134000	0.94660400
H	2.74891500	−0.43109500	−2.70447500
H	3.93622500	1.61993200	−1.98460000
H	3.80211900	2.36315300	0.37472100
H	2.45903500	1.05908200	1.98769300
C	−2.65021600	0.33024000	−0.11272800
C	−1.34590000	−0.31413000	−0.07317300
C	−0.24357500	0.55907800	0.20700600
C	−0.37550500	1.89722300	0.39347800
C	−1.65213100	2.46597700	0.31760900
C	−2.78115200	1.67933200	0.07279700
H	0.48780000	2.51461300	0.59502900
H	−1.77518400	3.53008100	0.45225700
H	−3.75880400	2.13090700	0.02650400
Cl	−4.01136600	−0.65203900	−0.41053100
O	−1.22100200	−1.52922200	−0.28211900
Cl	1.22895500	−1.45087400	1.74989900
O	1.31134900	−1.91256300	−1.22782700
H	0.50253700	−2.08113000	−0.71709500
TS93			
0	3		

C	2.01327300	2.61197100	0.18895200
C	0.78903900	2.59745000	−0.47481900
C	0.36609900	1.39097200	−0.94577100
C	1.01566200	0.17235200	−0.83352900
C	2.23937200	0.24200300	−0.13719000
C	2.73174800	1.43810600	0.35115200
H	2.40942600	3.53955300	0.57317200
H	0.21468300	3.50036000	−0.61343000
H	3.68229200	1.44445000	0.85919900
O	0.56680200	−0.95032500	−1.34996600
C	−0.67018200	−1.80759100	−0.40100800
C	−1.80137800	−0.93591600	−0.51133800
C	−2.06954600	−0.05748500	0.51199700
C	−1.35071600	−0.09639800	1.70021900
C	−0.37697500	−1.08305400	1.88934400
C	−0.09650100	−1.97621400	0.89462700
H	−1.57540500	0.61760400	2.47525000
H	0.13073200	−1.14481900	2.83858000
H	0.64208900	−2.74804700	1.03076700
H	−0.66461900	−2.64293400	−1.08192900
Cl	3.15533500	−1.20136800	0.07091700
Cl	−3.34569900	1.09129200	0.29868500
O	−2.46797300	−0.97350500	−1.66037900
H	−3.13301100	−0.28142100	−1.67790000
TS94			
0	3		
C	−3.37833200	−1.26221900	−0.28923100
C	−2.35165600	−1.99890800	−0.87419500
C	−1.17384100	−1.34529000	−1.08051900
C	−0.88435300	−0.02865600	−0.76903100
C	−1.94994600	0.66579200	−0.16331600
C	−3.17426300	0.06313100	0.05985900
H	−4.33976000	−1.71960300	−0.11204100
H	−2.49199800	−3.03107900	−1.15559100
H	−3.96745800	0.63997400	0.50680700
O	0.26426900	0.55535600	−1.04223300
C	1.44213800	−2.05190500	0.70256800
C	0.76422000	−1.71090400	1.86264900
C	0.58191300	−0.36483700	2.21730900
C	1.04893700	0.62945100	1.41203300
H	1.62057200	−3.08093700	0.43594000
H	0.39311000	−2.49000800	2.50888000
H	0.09806300	−0.11588500	3.14825500

H	0.94383000	1.67041200	1.66511200
Cl	−1.72610200	2.31866600	0.26453700
C	1.58379400	0.30700600	0.13080600
Cl	2.66568700	1.47664400	−0.57735300
C	1.90805100	−1.06958400	−0.13771900
O	2.55050800	−1.40250800	−1.25094200
H	2.75249300	−0.61439400	−1.76136700
TS95			
0	2		
C	0.47121400	0.40890300	1.35976400
C	1.30740800	−0.44406400	0.62169900
C	2.25382300	0.08856100	−0.22850100
C	2.44750900	1.45765200	−0.31279000
C	1.68900100	2.30157400	0.48549400
C	0.73716400	1.78420000	1.33868400
H	3.19618600	1.84596700	−0.98352000
H	1.86399700	3.36506700	0.44762200
H	0.15940000	2.43688200	1.97344600
C	−3.43437200	−0.91540300	−0.30575200
C	−2.16380700	−0.93797100	0.25922700
C	−1.41169500	0.21536300	0.17642500
C	−1.84422100	1.35660300	−0.44465100
C	−3.11064500	1.36559000	−1.01513700
C	−3.89822800	0.22774600	−0.93177700
H	−1.21364000	2.23366500	−0.48019800
H	−3.47759100	2.25156800	−1.51000000
H	−4.88677800	0.22557000	−1.36475000
O	−1.74902500	−2.06952500	0.86303600
H	−0.79113600	−2.06800700	0.94256300
H	−0.10674500	−0.02265400	2.16286600
Cl	3.21214100	−0.99742400	−1.17640100
H	−4.03746100	−1.80899900	−0.24765000
O	1.09463700	−1.76799800	0.74875700
H	1.66356400	−2.25519000	0.14796900
TS96			
0	2		
C	−1.25017800	−0.33598100	−0.42182600
C	−1.36203800	−0.27410600	0.98996000
C	−1.90042000	0.86278900	1.56365400
C	−2.38038300	1.89485400	0.77979900
C	−2.34366700	1.80162700	−0.60907500
C	−1.82629000	0.67767800	−1.20468400

H	−1.92847600	0.91210700	2.64025100
H	−2.79826200	2.77019400	1.25136300
H	−2.75083600	2.59176300	−1.21987600
H	−1.82765300	0.55870800	−2.27607400
C	3.09609900	−0.24726400	0.39853600
C	1.80971400	−0.69045900	0.14416500
C	0.89847600	0.18680200	−0.39886900
C	1.21970200	1.48413700	−0.70331100
C	2.51388400	1.92562300	−0.45534400
C	3.44206700	1.06077000	0.10044400
H	0.47655300	2.15130900	−1.11560700
H	2.78976000	2.94279600	−0.68861600
H	4.44505400	1.40342800	0.30196100
Cl	−1.11496400	−1.91166500	−1.18401000
H	3.80825500	−0.93689800	0.82577100
O	−0.93069000	−1.24990300	1.78820600
H	−0.19275200	−1.73396700	1.39263800
O	1.47468400	−1.97951100	0.48594100
H	1.07900200	−2.40463900	−0.27751800
TS97			
0	3		
C	0.41569300	1.38725600	−0.34625300
C	1.30364900	0.39132900	−0.78625400
C	2.26289200	−0.08501200	0.09007400
C	2.41337300	0.45067300	1.35913600
C	1.59259600	1.49335800	1.76129000
C	0.62465300	1.97741500	0.90523200
H	3.17858800	0.05885100	2.00935900
H	1.72712600	1.93067700	2.73764600
H	−0.01175600	2.79454600	1.20531400
C	−2.42525700	−1.75196300	1.09115200
C	−1.26997100	−1.00825300	0.84625500
C	−1.36683300	0.09275900	0.05124200
C	−2.58544100	0.54780300	−0.54451900
C	−3.74937800	−0.26246000	−0.26453000
C	−3.65544600	−1.36938200	0.52737500
H	−2.37734900	−2.62878100	1.71954600
H	−0.32172300	−1.29724700	1.27930800
H	−4.53329500	−1.96387000	0.72888600
O	−2.65354400	1.56917800	−1.25077800
H	−0.23061900	1.85252400	−1.07570500
Cl	3.30763300	−1.35855400	−0.44911200
H	−4.68301800	0.04896800	−0.70764700

O	1.13822000	−0.08287300	−2.02460100
H	1.78168000	−0.77183400	−2.19996800
TS98			
0	3		
C	1.19820200	−0.24519200	0.53717500
C	1.53951000	−0.52486500	−0.81255000
C	2.21902000	0.43628400	−1.54066800
C	2.61910900	1.62085000	−0.95198600
C	2.36139500	1.86264600	0.39564700
C	1.70117800	0.91949900	1.14367200
H	2.42751300	0.22320300	−2.57680000
H	3.15079700	2.35307200	−1.53910300
H	2.71163400	2.76936700	0.86322900
H	1.53397000	1.05715200	2.20007100
C	−3.17159800	−0.35242100	−0.47923800
C	−1.78485900	−0.73080500	−0.35270600
C	−0.89282600	0.28384500	0.12986800
C	−1.30507800	1.54660700	0.42771100
C	−2.65253900	1.87473000	0.26253000
C	−3.57705900	0.91507100	−0.18387800
H	−0.60058800	2.28557600	0.78212800
H	−2.99006500	2.87662200	0.48197800
H	−4.61421900	1.19125100	−0.29398100
O	−1.40668300	−1.87660700	−0.66665800
Cl	0.82459100	−1.58591900	1.58959000
H	−3.85604400	−1.11143600	−0.82581600
O	1.19529800	−1.66121400	−1.40386300
H	0.38328800	−2.01911700	−1.00403200
TS99			
0	3		
C	3.07927500	1.48735900	0.27390800
C	2.02531500	1.86731100	−0.55510400
C	1.23211600	0.87313100	−1.04736100
C	1.37819200	−0.48400200	−0.80291700
C	2.43566100	−0.82418000	0.05575300
C	3.27558300	0.14537500	0.57036900
H	3.74200300	2.23757100	0.67816200
H	1.85508600	2.90514900	−0.79894000
H	4.09293800	−0.14577700	1.21206800
O	0.61681900	−1.41280400	−1.35490300
C	−0.91322700	−1.77321600	−0.51382300
C	−1.61538400	−0.52757500	−0.56765900

C	−1.57198800	0.31101200	0.52201300
C	−0.98379800	−0.08855800	1.71460800
C	−0.47746500	−1.38738400	1.83549300
C	−0.51152800	−2.24277400	0.77143900
H	−0.95130700	0.59946400	2.54305600
H	−0.08094800	−1.70902800	2.78535200
H	−0.13472300	−3.24917200	0.85358700
H	−1.19646100	−2.49315000	−1.26399800
Cl	−2.28996600	1.87919000	0.38340300
H	2.57965200	−1.87022700	0.28299500
O	−2.18784900	−0.21732500	−1.72687900
H	−2.52507800	0.68098700	−1.69619300
TS100			
0	3		
C	3.74127800	−0.02452800	−0.21682500
C	2.95388500	0.42994500	−1.27246900
C	1.65360100	0.01872700	−1.30074300
C	1.04521200	−0.81585700	−0.37722100
C	1.86424800	−1.23808800	0.68131500
C	3.19004000	−0.85479500	0.75017600
H	4.77932100	0.26594500	−0.15727700
H	3.36419200	1.07378300	−2.03579200
H	3.80377000	−1.20732100	1.56474000
O	−0.20502500	−1.23747400	−0.48449600
C	−0.72964100	2.06264400	−0.16692300
C	−0.22262500	2.11848500	1.12100400
C	−0.42558700	1.05790100	2.01774600
C	−1.10117000	−0.05700200	1.62238900
H	−0.60960900	2.88328200	−0.85535800
H	0.31444500	2.99628300	1.44239900
H	−0.07002500	1.13824000	3.03266200
H	−1.29336300	−0.87896400	2.29181900
C	−1.46951400	−0.21184900	0.25581200
Cl	−2.78627500	−1.30884700	−0.07365800
C	−1.40329900	0.94394000	−0.59610700
H	1.42782100	−1.88777000	1.42562600
O	−1.88025900	0.89551300	−1.83477200
H	−2.24618500	0.02546200	−2.01141800

Table S5. Cartesian coordinates for reactions, intermediate and products involved in the formation routes of pre-intermediates for PCTA/PT/DT/DFs, x coordinate, y coordinate and z coordinate.

2-CTP			
0	1		
C	−2.40693700	0.60928100	−0.00002200
C	−1.23620400	1.34006300	0.00002600
C	−0.01153900	0.69432700	−0.00000900
C	0.06931900	−0.69486100	0.00012200
C	−1.12466300	−1.41295100	0.00012500
C	−2.34636100	−0.77451000	−0.00002300
H	−3.35675100	1.11961400	−0.00004500
H	−1.25583700	2.41795400	0.00002200
H	−1.08240200	−2.49162000	0.00031500
H	−3.25261900	−1.35939700	−0.00002500
Cl	1.41527100	1.67383000	−0.00006800
S	1.55185800	−1.63175800	−0.00023100
H	2.39658000	−0.60164200	0.00327600
CTPR1			
0	2		
C	2.35886100	−0.70747300	0.00002100
C	1.15370700	−1.38374900	0.00000500
C	−0.02793300	−0.66935500	0.00001200
C	−0.03619300	0.71959200	0.00006600
C	1.18466500	1.38841100	0.00002900
C	2.36906700	0.67780500	0.00001300
H	3.28401200	−1.26119000	0.00000800
H	1.11810600	−2.46130600	−0.00001600
H	1.20074400	2.46724900	0.00001000
H	3.30559400	1.21307700	−0.00000600
Cl	−1.54089100	−1.50376300	−0.00000700
S	−1.54539700	1.59092300	−0.00004700
CTPR2			
0	2		
C	2.46039800	0.41505600	−0.00009800
C	1.33466000	1.21607800	−0.00005600
C	0.06539400	0.65776500	−0.00002000
C	−0.11861400	−0.72779300	0.00017600
C	1.04973100	−1.44753900	0.00025300
C	2.32407400	−0.97057300	0.00001800
H	3.44082500	0.86541700	−0.00023300
H	1.42726600	2.28970800	−0.00006900

H	3.18167700	−1.62460900	−0.00003200
Cl	−1.29132600	1.73109800	0.00001100
S	−1.64828400	−1.58266200	−0.00017600
H	−2.41854100	−0.49454600	0.00133700
CTPDR			
0	3		
C	−2.37787700	0.57323600	0.00006600
C	−1.19262100	1.30183500	0.00021100
C	0.02874200	0.66154600	0.00011800
C	0.10178100	−0.75781300	−0.00012900
C	−1.12948800	−1.39266200	−0.00025900
C	−2.36460000	−0.81913300	−0.00017700
H	−3.31925400	1.10203500	0.00014400
H	−1.22018000	2.37903700	0.00039800
H	−3.27675600	−1.39379600	−0.00029200
Cl	1.46181300	1.59998200	0.00030200
S	1.53560900	−1.66806400	−0.00027200
TPR2			
0	2		
C	2.27496600	−0.04520900	0.00002000
C	1.57576100	−1.24875500	0.00000600
C	0.21325100	−1.16671600	−0.00003300
C	−0.52874800	−0.01733700	−0.00012400
C	0.19289500	1.17611200	−0.00004000
C	1.57729700	1.15225300	0.00003600
H	3.35411500	−0.04889800	0.00006900
H	2.09103700	−2.19690400	0.00003800
H	−0.33370000	2.11948200	−0.00002600
H	2.11728500	2.08627100	0.00009200
S	−2.28813000	0.01535900	0.00003000
H	−2.45120200	−1.30778100	0.00016300
TPDR			
0	3		
C	2.21279700	−0.03271900	−0.00001200
C	1.53539500	−1.24938700	0.00001100
C	0.17256500	−1.18220300	−0.00000800
C	−0.59592800	−0.02904700	−0.00007500
C	0.13229500	1.18357600	0.00000100
C	1.50854000	1.17123700	0.00000300
H	3.29254300	−0.02345800	0.00000500
H	2.06749000	−2.18780000	0.00003300

H	−0.41955600	2.11133100	0.00003300
H	2.05109500	2.10357500	0.00002800
S	−2.29909700	−0.07327400	0.00002400
2-CP			
0	1		
C	−1.81954100	−1.26813400	−0.00008700
C	−0.45026800	−1.45388200	−0.00021700
C	0.38749800	−0.35566600	−0.00009700
C	−0.11792600	0.93874400	0.00016300
C	−1.49369500	1.11335100	0.00030000
C	−2.33476200	0.01917900	0.00017400
H	−2.47739100	−2.12216600	−0.00018100
H	−0.02189200	−2.44319600	−0.00041300
H	−1.87689400	2.12126600	0.00050200
H	−3.40232800	0.17426500	0.00028100
O	0.66604100	2.02579800	0.00028400
H	1.58739100	1.76066400	0.00029100
Cl	2.10793800	−0.56816300	−0.00024500
CPR1			
0	2		
C	1.70263800	−1.33082400	−0.00002200
C	0.31585600	−1.44405400	−0.00000800
C	−0.46131600	−0.31320000	−0.00002200
C	0.13568900	1.00842800	−0.00013600
C	1.57378900	1.06006200	−0.00000300
C	2.32696600	−0.07612200	0.00000800
H	2.30188400	−2.22785900	0.00000100
H	−0.15348600	−2.41463400	0.00002200
H	2.01535500	2.04395200	0.00004700
H	3.40405700	−0.01686400	0.00004900
O	−0.55080200	2.03838600	0.00004600
Cl	−2.16018400	−0.41867100	0.00003700
CPR2			
0	2		
C	−1.93114600	−1.11695700	−0.00009100
C	−0.57428700	−1.38058900	−0.00021900
C	0.33734100	−0.33886900	−0.00008300
C	−0.06937700	0.99461500	0.00018300
C	−1.42956900	1.17926200	0.00030000
C	−2.37771300	0.20319000	0.00017700
H	−2.63871200	−1.93101900	−0.00019800

H	−0.20752000	−2.39398800	−0.00042400
H	−3.43120100	0.43657600	0.00028300
O	0.77295700	2.03311700	0.00031800
H	1.67647900	1.71180500	0.00023300
Cl	2.04034200	−0.66660100	−0.00023800
CPDR			
0	3		
C	−1.82600500	−1.18598200	−0.00006700
C	−0.44095400	−1.37465300	−0.00020100
C	0.41088900	−0.30243300	−0.00008300
C	−0.08675100	1.07163800	0.00018600
C	−1.51357100	1.13863700	0.00030300
C	−2.38160700	0.10099900	0.00019200
H	−2.47511600	−2.04820300	−0.00016300
H	−0.03463200	−2.37302200	−0.00039800
H	−3.45104400	0.24403000	0.00029400
O	0.66181300	2.04952700	0.00030000
Cl	2.09966400	−0.52401500	−0.00024200
PR2			
0	2		
C	1.83148600	−0.00768400	0.00018100
C	1.16100800	−1.22746600	0.00030700
C	−0.20260800	−1.18500000	0.00016700
C	−0.97014400	−0.04829800	−0.00008800
C	−0.28339800	1.16028600	−0.00021200
C	1.10082700	1.17060700	−0.00007600
H	2.91008300	0.01439100	0.00028400
H	1.69937300	−2.16301300	0.00050800
H	−0.85387700	2.07681000	−0.00041500
H	1.61697200	2.11799600	−0.00017500
O	−2.31859900	−0.03595400	−0.00021500
H	−2.64678200	−0.93322900	−0.00015000
PDR			
0	3		
C	1.76165200	0.04596300	−0.00002100
C	1.15035800	−1.21191500	0.00001600
C	−0.20631600	−1.24273600	−0.00000400
C	−1.07646000	−0.10866500	−0.00017500
C	−0.37670300	1.15955000	−0.00001000
C	0.98647200	1.21820000	0.00000200
H	2.83885000	0.11475600	0.00000700

H	1.74200500	−2.11481200	0.00007000
H	−0.98995100	2.04789800	0.00006100
H	1.48422000	2.17546000	0.00005200
O	−2.31364300	−0.17321100	0.00012000
IM1			
0	2		
C	2.81525700	2.42741800	0.27927500
C	1.50886900	2.58067100	−0.15196400
C	0.77737400	1.48042000	−0.55941600
C	1.33939400	0.21407100	−0.52592100
C	2.65315600	0.07064200	−0.09457800
C	3.39043900	1.17043000	0.30328300
H	3.39179400	3.28379100	0.59156400
H	1.05891800	3.56050100	−0.18396900
H	4.40837100	1.03108900	0.62967700
O	0.66639700	−0.88151900	−0.96599500
C	−0.40822600	−1.36617500	−0.14627900
C	−1.66600200	−0.57928300	−0.38451200
C	−2.34984400	−0.03854800	0.66821800
C	−1.92574100	−0.19106400	1.99325300
C	−0.74806600	−0.91016200	2.27151400
C	−0.01632600	−1.46541200	1.28116800
H	−2.50205700	0.25788500	2.78414900
H	−0.43097000	−1.01134200	3.29791300
H	0.89090200	−2.01162300	1.48849200
H	−0.22939300	1.59149700	−0.92811700
H	−0.55757200	−2.36884200	−0.55479600
Cl	3.37169800	−1.49574300	−0.05990700
Cl	−3.79856000	0.87746700	0.41282300
S	−2.06497500	−0.44924600	−2.06890000
H	−3.28661500	0.05977200	−1.89838000
IM2			
0	1		
C	3.48996600	−1.43044700	0.88153600
C	2.27290500	−1.92945100	1.31231300
C	1.09450900	−1.29979200	0.95771800
C	1.12665400	−0.16697900	0.16135000
C	2.34876100	0.33499800	−0.26708600
C	3.52522700	−0.29426700	0.09429600
H	4.41065100	−1.91907600	1.15741600
H	2.23536800	−2.80971900	1.93455700
H	4.46159600	0.11376600	−0.25052900

O	0.01210200	0.52664500	−0.18089700
C	−1.18471100	−0.12130800	−0.32056300
C	−2.29993200	0.44851000	0.28103400
C	−3.53520900	−0.15987800	0.08867200
C	−3.65003100	−1.30424200	−0.67546800
C	−2.52914000	−1.85514400	−1.27534300
C	−1.29619700	−1.25649200	−1.10231200
H	−4.41106700	0.27590100	0.54468200
H	−4.61819600	−1.76057200	−0.80888100
H	−2.61376800	−2.74122500	−1.88417300
H	−0.41008300	−1.66067300	−1.56713800
H	0.14161000	−1.67615000	1.29482000
Cl	2.39387200	1.74964900	−1.24640500
S	−2.20233700	1.89132800	1.27524400
H	−0.92816800	2.16625300	0.99450600
IM3			
0	2		
C	−3.37968900	1.45057000	−0.46153500
C	−3.41414700	0.13940000	−0.73679500
C	−2.46161500	−0.77387300	−0.11263400
C	−1.18861300	−0.13398100	0.43357500
C	−1.38742200	1.26291100	0.89599600
C	−2.38777800	2.01140800	0.42855100
C	−0.15192900	−0.15166600	−0.81368600
C	1.15652200	0.48729500	−0.46463100
C	0.00575900	−1.54323800	−1.30784800
C	2.23196900	−0.30935300	−0.16838700
C	1.11481900	−2.26700800	−1.04222900
C	2.22001100	−1.68670800	−0.39440200
O	−2.62936400	−1.96789400	−0.09454000
H	3.08567200	−2.27203500	−0.13502700
H	−0.65568900	1.66215000	1.57887700
H	−0.82934200	−1.99557200	−1.81538100
H	−4.13563600	2.10580400	−0.86886200
H	1.16630900	−3.30214900	−1.34146900
H	−2.47878700	3.04332100	0.72862100
H	−4.19742700	−0.31009600	−1.32599000
H	−0.65982000	0.46610800	−1.55902600
Cl	−0.53334500	−1.11173600	1.75489900
S	1.18215000	2.22665300	−0.52186700
H	2.49668800	2.36640700	−0.33817200
Cl	3.72047500	0.37280800	0.40079300

IM4			
0	2		
C	2.84317400	−1.60798900	0.19549500
C	2.98301500	−0.27930400	0.07817700
C	1.93109200	0.62967200	0.55256700
C	0.57039000	0.01649100	0.76621200
C	0.58849000	−1.43473400	1.05533400
C	1.64697200	−2.19114800	0.75401000
C	−0.28932800	0.32564900	−0.56714200
C	−1.66072400	−0.26104900	−0.43847600
C	−0.32673000	1.79706100	−0.76949500
C	−2.66089900	0.52202300	0.07547700
C	−1.36507600	2.53007900	−0.30685000
C	−2.51044300	1.90434900	0.21937400
O	2.11853100	1.81240800	0.70936700
H	−3.31872600	2.48123100	0.63620800
H	0.24203000	−0.16934200	−1.38044900
H	−0.30046300	−1.87738900	1.47534300
H	0.54340600	2.27679700	−1.18407700
H	3.65530800	−2.25496600	−0.09928100
H	0.07255300	0.57842000	1.55384300
H	−1.33167200	3.60726100	−0.35411600
H	1.63251900	−3.25518800	0.92948500
Cl	4.42738300	0.43714400	−0.50221800
Cl	−4.21671000	−0.14395800	0.45413600
S	−1.81808500	−1.91021600	−0.95969700
H	−3.13488500	−2.01340100	−0.76743700
IM5			
0	1		
C	−2.88679100	1.15762600	0.52871300
C	−2.52852100	0.03725300	−0.11516300
C	−1.14484700	−0.44901700	−0.09514900
C	−0.16309500	0.30305700	0.78756700
C	−0.66851300	1.55968900	1.39528500
C	−1.94253000	1.93697300	1.29261900
C	1.17719900	0.46040800	0.11774000
C	2.14437300	−0.54478000	0.17353600
C	1.46358900	1.62878500	−0.57468000
C	3.37844500	−0.34359200	−0.42951800
C	2.69221800	1.82194000	−1.17924200
C	3.65694200	0.83465300	−1.09717800
O	−0.79547500	−1.42431800	−0.71693600
H	4.62083900	0.97285900	−1.56189900

H	0.03937400	2.14361100	1.96485800
H	0.70871900	2.39991800	−0.63148200
H	−3.91105600	1.49269200	0.46732300
H	−0.00587500	−0.39930900	1.62130900
H	2.89230500	2.73972000	−1.70994100
H	−2.29591000	2.83260600	1.77844600
H	4.11638000	−1.12861600	−0.38114400
Cl	−3.64542800	−0.86243200	−1.05205000
S	1.89170100	−2.05995400	1.05658000
H	0.83326400	−2.45630900	0.34041000
IM6			
0	1		
C	3.18634700	0.21264500	−1.35623500
C	3.02417700	0.95944900	−0.25836300
C	1.81517900	0.87371300	0.54490900
C	0.74184000	−0.16259100	0.14338300
C	1.05742100	−0.91807100	−1.10391700
C	2.17957500	−0.72629700	−1.79457400
C	−0.65093000	0.43083100	0.03809500
C	−1.81937600	−0.33662100	−0.11518000
C	−0.78066700	1.81700100	−0.01361600
C	−3.04185100	0.30277600	−0.28687000
C	−1.99820200	2.43926100	−0.21191100
C	−3.14023900	1.67763900	−0.34758700
O	1.65176900	1.55965900	1.52431000
H	−4.10343500	2.14306800	−0.48708100
H	0.31294200	−1.61956200	−1.44198600
H	0.08746100	2.43828200	0.10703100
H	4.09206400	0.30255900	−1.93775800
H	−2.04411700	3.51609100	−0.24803200
H	2.34974600	−1.28159400	−2.70352600
H	−3.92989900	−0.30476600	−0.36790300
H	3.76823000	1.65956500	0.08586700
Cl	0.88602900	−1.33627800	1.54025900
S	−1.95221700	−2.10061400	−0.21788400
H	−1.01381400	−2.42277800	0.67184100
IM7			
0	2		
C	1.97040400	2.67468900	−0.46605400
C	0.82832400	2.42136800	−1.20555700
C	0.43602300	1.11622600	−1.42684300
C	1.15941200	0.04409600	−0.91050000

C	2.31467800	0.32166000	−0.18016800
C	2.71754100	1.62756600	0.03982300
H	2.29119500	3.68936500	−0.28778800
H	0.24584600	3.23406800	−1.60972800
H	3.61609500	1.81259500	0.60575300
Cl	3.30074200	−0.94163900	0.45729200
C	−0.67064500	−1.75932800	0.21853500
C	−1.74617300	−0.75291800	0.08200200
C	−1.74318100	0.33691000	0.91280900
C	−0.86196500	0.44578300	1.99269500
C	0.01739800	−0.61084100	2.28153400
C	0.07342200	−1.70350400	1.48202300
H	−0.89412200	1.32781600	2.60954500
H	0.63948800	−0.55066400	3.16087500
H	0.74432500	−2.51945300	1.69690700
H	−0.44976600	0.89915600	−2.00458600
H	−1.04236600	−2.75960000	0.00245600
Cl	−2.87012700	1.63233900	0.68655500
S	0.58570300	−1.59044900	−1.21317800
S	−2.81628000	−1.01596200	−1.26484800
H	−3.75333600	−0.14286000	−0.89222700
IM8			
0	1		
C	−2.94764600	2.20157700	−0.49051200
C	−1.59054600	2.40484600	−0.66669300
C	−0.70638300	1.34732100	−0.57155000
C	−1.16143300	0.06450600	−0.29627700
C	−2.52934300	−0.12366100	−0.12325200
C	−3.41743900	0.93058000	−0.21883800
H	−3.64056800	3.02469300	−0.56300700
H	−1.21141200	3.39228000	−0.87825500
H	−4.46989600	0.74354800	−0.07877100
C	1.50253900	−0.62040200	−0.33552000
C	2.14005400	0.05628500	0.71172800
C	3.42707800	0.54479800	0.50624600
C	4.07398700	0.35926500	−0.69891300
C	3.45126400	−0.32576800	−1.72882200
C	2.17172100	−0.80978000	−1.53894600
H	3.92563400	1.06620400	1.30923100
H	5.07314200	0.74559200	−0.82888800
H	3.95399900	−0.47938700	−2.67034400
H	1.66458400	−1.34004800	−2.32985400
H	0.34949600	1.51565000	−0.71061100

Cl	−3.14435000	−1.70287700	0.21400400
S	−0.09946700	−1.34296100	−0.16439700
S	1.40911900	0.37756900	2.27138000
H	0.37143100	−0.45075300	2.10878900
IM9			
0	2		
C	−2.86520500	2.02620500	−0.60402100
C	−3.11490100	0.71152400	−0.77487100
C	−2.38375300	−0.29230000	−0.04765200
C	−1.05156000	0.16760200	0.47807000
C	−1.04569900	1.58636700	0.90131400
C	−1.86856600	2.47193900	0.32847800
C	−0.00621100	0.03253600	−0.83666600
C	1.37520100	0.44307400	−0.46735900
C	−0.07420300	−1.33482500	−1.38112000
C	2.31806800	−0.52448700	−0.21395900
C	0.90150200	−2.24206600	−1.14930400
C	2.08533400	−1.86925700	−0.49166400
H	2.84732700	−2.59495200	−0.26252700
H	−0.30455200	1.88306100	1.62395800
H	−0.96115800	−1.61685300	−1.92211200
H	−3.47294700	2.75754500	−1.11504300
H	0.78709600	−3.25875900	−1.49085300
H	−1.80667300	3.51816300	0.58291800
H	−3.94083200	0.37355600	−1.37997100
Cl	−0.41954300	−0.85084600	1.77477100
H	−0.44585900	0.75726300	−1.52370200
Cl	3.89075500	−0.11049200	0.38201600
S	−2.93764000	−1.81855100	0.08147400
S	1.66823500	2.15528700	−0.42216000
H	2.97743500	2.07809000	−0.17453300
IM10			
0	2		
C	−2.50713200	1.88058300	0.09869200
C	−2.75208900	0.55767100	−0.02886900
C	−1.85316400	−0.41775900	0.53981100
C	−0.46846200	0.08263100	0.78879600
C	−0.39128600	1.50268100	1.17324100
C	−1.35047900	2.35843300	0.79770600
C	0.38746200	−0.12182100	−0.62675100
C	1.78838700	0.33773400	−0.42144300
C	0.31167500	−1.54070300	−1.02172500

C	2.72911300	−0.58098100	−0.02545300
C	1.29116000	−2.41095200	−0.67844400
C	2.47711400	−1.95212900	−0.08106700
H	3.23831000	−2.64080400	0.24531800
H	−0.12831500	0.52550900	−1.33492900
H	0.48547700	1.84659900	1.69701900
H	−0.58488900	−1.88735400	−1.50696500
H	−3.23020400	2.58726100	−0.27774700
H	0.03630300	−0.57462300	1.48993400
Cl	4.32308600	−0.08919200	0.44547100
H	1.17584200	−3.46503500	−0.87563100
H	−1.27497000	3.41180600	1.01601900
Cl	−4.21824800	0.03583600	−0.75961000
S	−2.25342200	−1.96836100	0.84326200
S	2.07435600	2.02651300	−0.70044500
H	3.39145300	2.00093100	−0.48470200
IM11			
0	1		
C	−2.69968900	1.29088200	0.72247000
C	−2.42685700	0.17902100	0.00735900
C	−1.15007400	−0.48732700	0.08925000
C	−0.09210100	0.18075800	0.94186500
C	−0.51485200	1.37930000	1.70897100
C	−1.74069600	1.89409000	1.60528000
C	1.14297700	0.49924600	0.12127900
C	2.28252900	−0.30474300	0.15706600
C	1.13605600	1.62748400	−0.68683400
C	3.39409700	0.05539900	−0.59458000
C	2.24234700	1.97649800	−1.43779800
C	3.37984200	1.19117000	−1.38238400
H	4.25182500	1.45159600	−1.96203100
H	0.22730100	1.82356800	2.35519000
H	0.24598100	2.23994000	−0.72051800
H	−3.67842100	1.73609300	0.63124900
H	0.22218300	−0.56927300	1.67557000
H	2.21493000	2.85657300	−2.06122100
H	−2.03365800	2.76452200	2.17038600
H	4.26797600	−0.57611500	−0.56710300
Cl	−3.64539300	−0.46818200	−1.01684200
S	−0.82197700	−1.88464900	−0.68667000
S	2.39673700	−1.75826400	1.16423200
H	1.33592500	−2.37188200	0.61212500

IM12			
0	1		
C	−2.77946600	−0.37766000	1.85725700
C	−2.85875800	0.45747600	0.80339700
C	−1.85103100	0.50740000	−0.21145400
C	−0.62028000	−0.39125400	−0.03877800
C	−0.67838000	−1.30691900	1.13879900
C	−1.67319100	−1.27721100	2.02613700
C	0.68337700	0.39359700	0.07681900
C	1.96690500	−0.17928100	0.01341400
C	0.60107200	1.74854700	0.39188300
C	3.08353900	0.62817800	0.20220900
C	1.71774800	2.53094400	0.61376000
C	2.97246500	1.96953500	0.50672100
H	3.86095900	2.56350600	0.65324500
H	0.16064600	−1.97233300	1.26028800
H	−0.36390000	2.21428300	0.47945800
H	−3.57281800	−0.38550300	2.58949000
H	1.59593400	3.57401900	0.85880900
H	−1.65213200	−1.93316200	2.88196700
H	4.05941600	0.17825800	0.10430500
H	−3.70745500	1.10860500	0.66973700
Cl	−0.66857000	−1.44971600	−1.52519400
S	2.39878900	−1.88876100	−0.16289700
H	1.44651400	−2.25507000	−1.01971500
S	−2.01413100	1.47203900	−1.51399500
IM13			
0	2		
C	1.56991900	2.79887300	−0.00060700
C	0.52372600	2.56418000	−0.87601000
C	0.27942400	1.27755300	−1.31317100
C	1.05632100	0.20367400	−0.88559700
C	2.11426000	0.46611500	−0.01581200
C	2.36910700	1.75400100	0.42326700
H	1.77517500	3.79903700	0.34837800
H	−0.09725200	3.37704800	−1.21782900
H	3.19477200	1.92743900	1.09419500
Cl	3.16108200	−0.79047000	0.53073400
C	−0.67847000	−1.88376600	−0.19505000
C	−1.80353800	−0.94567300	−0.28749600
C	−1.98792600	0.00703300	0.67173000
C	−1.21981100	0.04238800	1.83938200
C	−0.27945400	−0.97627700	2.06177800

C	−0.06355700	−1.94661100	1.13606200
H	−1.38457900	0.82383800	2.56141200
H	0.26199300	−0.99333200	2.99474300
H	0.65482500	−2.73087200	1.31038600
O	−2.53748400	−1.04863700	−1.39697500
H	−3.22593600	−0.37949000	−1.39310700
H	−0.52856300	1.07408900	−2.00002000
H	−0.96008200	−2.85773400	−0.59114700
Cl	−3.23845800	1.18430100	0.42587600
S	0.66530700	−1.40718700	−1.47152900
IM14			
0	1		
C	−2.88400800	2.25645300	0.13944500
C	−1.52178800	2.49660000	0.10816900
C	−0.62884900	1.44816900	−0.01025100
C	−1.08118300	0.13841600	−0.09659800
C	−2.45361900	−0.08668500	−0.06389100
C	−3.34998800	0.95844500	0.05288200
H	−3.58354000	3.07205700	0.23083500
H	−1.14548300	3.50528800	0.17587000
H	−4.40614300	0.74314400	0.07441300
C	1.58686200	−0.51217700	−0.27520200
C	2.25986400	−0.30950100	0.93135900
C	3.53336300	0.24360600	0.92063000
C	4.12702500	0.58046600	−0.27789900
C	3.47100600	0.37040000	−1.48345000
C	2.20486800	−0.17819000	−1.47406800
H	4.03623700	0.39538600	1.86220400
H	5.11798300	1.00800600	−0.27248400
H	3.94374100	0.62915000	−2.41713700
H	1.67312000	−0.35367600	−2.39648100
O	1.71625900	−0.61919200	2.11345100
H	0.86571500	−1.04468400	1.96076400
H	0.43117300	1.64375800	−0.03784400
Cl	−3.06601700	−1.69923400	−0.16605700
S	−0.00761800	−1.26212000	−0.25572100
IM15			
0	2		
C	−2.89686000	1.89312200	−0.81884300
C	−3.06713400	0.55407500	−0.84869000
C	−2.27260900	−0.32275800	−0.03329400
C	−0.97716300	0.27260700	0.43984400

C	−1.04779900	1.72625000	0.70297900
C	−1.92383100	2.49467400	0.04670100
C	0.08142100	0.05774400	−0.86072200
C	1.40516900	0.59753700	−0.51173500
C	0.13783700	−1.35538300	−1.26657500
C	2.46875800	−0.22710900	−0.26853600
C	1.22175800	−2.13586500	−1.02768300
C	2.39815600	−1.60083300	−0.48144100
H	3.25325600	−2.21994800	−0.26966100
O	1.47222200	1.92531600	−0.41384700
H	2.35935900	2.18285200	−0.14746200
H	−0.31715000	2.14487800	1.37292800
H	−0.73691400	−1.77898200	−1.72903500
H	−3.55222500	2.52775700	−1.39615600
H	1.19281700	−3.18330800	−1.28347400
H	−1.92010300	3.56451400	0.18168100
H	−3.87500300	0.10623700	−1.40484900
Cl	−0.27806700	−0.56897800	1.82652600
H	−0.39147400	0.69255900	−1.61253800
Cl	3.95874000	0.49292700	0.24901000
S	−2.72050000	−1.86691200	0.24269900
IM16			
0	2		
C	−2.50487000	1.84998100	−0.22752900
C	−2.66509600	0.50925300	−0.13674200
C	−1.71948400	−0.29928800	0.58829900
C	−0.38328100	0.33108400	0.77596000
C	−0.40039000	1.79392200	0.92805700
C	−1.40037200	2.51152200	0.39815100
C	0.50693700	−0.02174700	−0.60983900
C	1.86906600	0.50410200	−0.43121800
C	0.49464400	−1.46848400	−0.87437800
C	2.91142800	−0.32651000	−0.12590600
C	1.56243500	−2.26146700	−0.59769600
C	2.77775600	−1.71419200	−0.16186200
H	3.62045900	−2.33821600	0.08338800
H	−0.01717900	0.55109500	−1.37524700
O	1.98987500	1.82844600	−0.52937900
H	2.90512800	2.08069000	−0.37828100
H	0.44115600	2.27575800	1.39649800
H	−0.41860700	−1.90337700	−1.24335700
H	−3.26234100	2.43567900	−0.72465300
H	0.15484600	−0.18164600	1.56557100

Cl	4.45700100	0.38589700	0.21158000
H	1.48685900	−3.32888600	−0.73243300
H	−1.39653700	3.58908500	0.43838300
Cl	−4.08303600	−0.21778800	−0.78339700
S	−2.00517700	−1.81966600	1.11089300
IM17			
0	1		
C	−2.38959500	1.55875700	0.21714900
C	−2.19603200	0.27252600	−0.14320400
C	−1.05643500	−0.47608200	0.32215400
C	0.02125500	0.30795200	1.03960100
C	−0.33683300	1.68858700	1.45101400
C	−1.47011100	2.27154900	1.05978600
C	1.30238000	0.32597100	0.21806900
C	2.25590100	−0.67539000	0.39783600
C	1.55441100	1.34266200	−0.69188800
C	3.45981500	−0.61090200	−0.28646300
C	2.74453100	1.39346300	−1.39292600
C	3.70362300	0.41829000	−1.17480800
H	4.64175400	0.45116000	−1.70712900
H	0.38488500	2.21326500	2.05882700
H	0.80908800	2.11041600	−0.84081500
H	−3.27822100	2.06326300	−0.12993300
H	0.25875300	−0.25277700	1.94716100
H	2.92385900	2.19120800	−2.09612300
H	−1.71366400	3.28018900	1.35301200
H	4.18336700	−1.39183600	−0.11491100
Cl	−3.36470600	−0.50365800	−1.13477900
O	2.06199100	−1.69891600	1.25708400
H	1.17109300	−2.05491600	1.11968400
S	−0.88964400	−2.08504700	0.09668000
IM18			
0	1		
C	−2.62972800	−0.14272300	1.87868300
C	−2.58101900	0.75276700	0.87307100
C	−1.59972700	0.68231400	−0.16590100
C	−0.54324300	−0.41507700	−0.06730500
C	−0.73144200	−1.37568200	1.05948300
C	−1.69118500	−1.22676100	1.97353700
C	0.86825600	0.12261200	0.05365500
C	1.98512500	−0.71526900	−0.07486100
C	1.08825400	1.44010600	0.44776500

C	3.25864800	−0.22286300	0.16025500
C	2.35696100	1.92516800	0.69441000
C	3.44773300	1.08765900	0.54622500
H	4.44666400	1.45280000	0.72799600
H	−0.00832300	−2.17319400	1.12812100
H	0.24871300	2.10062300	0.57765900
H	−3.39922700	−0.05488100	2.63081600
H	2.48669500	2.95121200	0.99877700
H	−1.76534700	−1.91816600	2.79780200
H	4.08739400	−0.90017300	0.02999400
H	−3.30473800	1.54818600	0.79801000
Cl	−0.75554300	−1.39648600	−1.60710800
O	1.90758200	−2.02502900	−0.38874400
H	1.12686900	−2.20114200	−0.92014300
S	−1.60668300	1.72885700	−1.41327100
IM19			
0	2		
C	−0.57980800	0.39757400	−1.03041100
C	−1.58014400	−0.47050800	−0.30888100
C	−2.78999700	0.03943700	0.06314900
C	−3.20142600	1.33639300	−0.27801900
C	−2.36026600	2.14451500	−1.06723100
C	−1.13800600	1.71452400	−1.45079900
H	−4.16940300	1.68266600	0.04127800
H	−2.70942600	3.11958000	−1.37112600
H	−0.49594200	2.33739900	−2.05461700
C	2.95749200	0.13992100	0.32787800
C	1.84389900	−0.05715400	−0.48025400
C	0.65927500	0.60171000	−0.17038700
C	0.61883500	1.42996200	0.93799100
C	1.73029900	1.61511600	1.73935300
C	2.91153400	0.96611700	1.43115900
H	−0.30890900	1.93336400	1.16919800
H	1.68117600	2.26152300	2.60071100
H	3.79502900	1.09226800	2.03567100
O	1.86803200	−0.86779800	−1.55087300
H	2.73168500	−1.27702600	−1.62879600
H	−0.25105400	−0.14252100	−1.92172300
Cl	4.42165000	−0.69160800	−0.08322000
Cl	−3.92658300	−0.91851500	0.95913900
S	−0.99994100	−2.08256300	−0.01826600
H	−2.06460700	−2.51743200	0.65672900

IM20			
0	1		
C	−1.27554200	0.51244000	−0.08486900
C	−1.98624500	−0.61097200	0.34542300
C	−3.33335000	−0.73376100	0.02337100
C	−3.97699700	0.24011200	−0.71352400
C	−3.28649800	1.36651500	−1.12576600
C	−1.94867300	1.49225600	−0.80605000
H	−3.88167700	−1.59788500	0.36715200
H	−5.02223100	0.12168900	−0.95409800
H	−3.78234700	2.13684500	−1.69502700
H	−1.39352200	2.35861600	−1.13365900
C	2.46739800	0.13780400	−0.11622700
C	1.12445400	−0.09774600	−0.38621100
C	0.15912500	0.71675500	0.21082700
C	0.57479900	1.75027100	1.03696000
C	1.91592000	1.97896200	1.29259500
C	2.86993000	1.16483200	0.71350600
H	−0.17853500	2.37596600	1.49068500
H	2.21805500	2.78438100	1.94221500
H	3.92165100	1.31625800	0.89519200
Cl	3.64292300	−0.88878200	−0.86802800
O	0.71200800	−1.07200200	−1.20306100
H	1.46405200	−1.57442900	−1.52127000
S	−1.29055200	−1.90361100	1.31461000
H	−0.14830200	−1.28316000	1.61581000
IM21			
0	2		
C	0.65097900	0.02057100	0.99442200
C	1.72953800	−0.51276500	0.08695300
C	2.93873400	0.11563300	0.01311300
C	3.26633800	1.22115700	0.81126800
C	2.33690100	1.69228700	1.75837600
C	1.11480300	1.12848000	1.87917800
H	4.23683200	1.67553200	0.70862100
H	2.61615300	2.51462100	2.39909900
H	0.40397100	1.49159700	2.60548100
C	−2.84686900	0.41932500	−0.47761800
C	−1.81477500	−0.08632800	0.31526400
C	−0.54878100	0.48728600	0.17723500
C	−0.34676200	1.52404200	−0.71838700
C	−1.37869200	2.00764600	−1.49503400
C	−2.63602300	1.44964400	−1.37272600

H	0.64495900	1.94676100	−0.79801400
H	−1.21115200	2.81295000	−2.19211000
H	−3.46288100	1.80537100	−1.96549700
Cl	−4.45215300	−0.22410800	−0.38151700
H	0.32800500	−0.80957100	1.62836100
Cl	4.17145200	−0.44832800	−1.06929600
S	−2.02081900	−1.39775300	1.46965500
H	−3.30621900	−1.62440300	1.20419400
S	1.22997400	−1.91836500	−0.80213300
H	2.38341800	−2.12541500	−1.43893700
IM22			
0	1		
C	−1.29556700	0.34615200	−0.43716700
C	−2.07727600	0.05777700	0.68389200
C	−3.41085900	−0.29833700	0.51424700
C	−3.96741800	−0.36740600	−0.74685300
C	−3.19990800	−0.08213200	−1.86327000
C	−1.87426800	0.26959900	−1.69882700
H	−4.01302700	−0.52408200	1.38138200
H	−5.00378100	−0.64760800	−0.85573200
H	−3.62736100	−0.13665800	−2.85190200
H	−1.25999200	0.48774600	−2.55958400
C	2.44828900	0.16750600	−0.13130900
C	1.12141100	−0.24559800	−0.23239800
C	0.12889900	0.74065300	−0.32040900
C	0.47758900	2.08114300	−0.31810700
C	1.80115400	2.46983300	−0.21942800
C	2.78668000	1.50858600	−0.12295200
H	−0.30698400	2.81873100	−0.38589700
H	2.06588800	3.51503400	−0.21429100
H	3.82546100	1.78482500	−0.04244900
Cl	3.73937400	−0.97878400	−0.01857100
S	0.58310100	−1.91382900	−0.26318400
H	1.78259000	−2.45239700	−0.04391000
S	−1.48635200	0.13292500	2.33795000
H	−0.21249600	0.40155400	2.04733100
IM23			
0	2		
C	0.74138100	−0.41791600	0.85515900
C	1.75579000	−0.58197200	−0.22540000
C	3.00152200	−0.04968400	−0.12866800
C	3.43896800	0.64212900	1.01306600

C	2.55691400	0.76861500	2.10144800
C	1.29880700	0.26758200	2.05877200
H	4.43636400	1.04536900	1.04358200
H	2.89921200	1.27535500	2.99082200
H	0.63332200	0.37421900	2.90120100
O	1.30615300	−1.27490800	−1.27219200
H	1.99039900	−1.33605900	−1.94304900
C	−2.77492200	0.51092600	−0.29775600
C	−1.72811700	−0.26757100	0.19952900
C	−0.47180700	0.33178000	0.31796400
C	−0.29300100	1.65336700	−0.05394700
C	−1.34065000	2.40589400	−0.54426400
C	−2.59025900	1.83008100	−0.66211300
H	0.69125600	2.08744800	0.05229000
H	−1.19103400	3.43431300	−0.83174000
H	−3.42936200	2.39214100	−1.03846600
Cl	−4.36901500	−0.14091500	−0.48199600
H	0.42038200	−1.42402600	1.14028500
Cl	4.09032400	−0.26644900	−1.46391700
S	−1.90574600	−1.95724800	0.65548100
H	−3.23784200	−1.96771800	0.65268100
IM24			
0	1		
C	−1.40660400	0.40721000	−0.12988200
C	−2.04590900	−0.25851100	0.91638900
C	−3.39140400	−0.57902000	0.82185400
C	−4.10436400	−0.23852000	−0.30944200
C	−3.48696200	0.42785300	−1.35674000
C	−2.14625900	0.74580100	−1.25664000
H	−3.85573900	−1.09349000	1.64794100
H	−5.15055700	−0.49556700	−0.37351500
H	−4.04266100	0.69347700	−2.24179100
H	−1.64707100	1.25945900	−2.06481700
O	−1.40955300	−0.59802100	2.05214400
H	−0.47411100	−0.40639600	1.97868600
C	2.35388200	0.20308500	−0.03836700
C	1.02339300	−0.19812600	−0.15089100
C	0.02383300	0.77873900	−0.02202100
C	0.37560600	2.10249500	0.19495700
C	1.70126300	2.48029100	0.29709500
C	2.69132100	1.52489100	0.18562700
H	−0.41198100	2.83365300	0.29116600
H	1.96519400	3.51140000	0.46932200

H	3.73246800	1.79183300	0.26676900
Cl	3.64917300	−0.93379400	−0.18750000
S	0.49400000	−1.83813100	−0.48522500
H	1.69417500	−2.40273500	−0.35145400
IM25			
0	2		
C	−0.00397400	0.48376400	0.87951800
C	1.07672400	−0.32779500	0.21535100
C	2.28886700	0.22191900	−0.07528000
C	2.59537700	1.56111700	0.21038900
C	1.60976500	2.38613700	0.78400000
C	0.38167000	1.90941200	1.08546200
H	3.57234900	1.94262200	−0.03201900
H	1.84649300	3.42101900	0.97888100
H	−0.37157600	2.55151900	1.51578500
C	−3.56005900	−0.55088900	−0.02298100
C	−2.32362700	−0.51523900	0.60378100
C	−1.33620100	0.36242300	0.16099100
C	−1.60414300	1.15610700	−0.94754200
C	−2.82786900	1.11330200	−1.58547400
C	−3.81256900	0.26156000	−1.10940400
H	−0.83375700	1.82924700	−1.29601700
H	−3.01492300	1.74294700	−2.44091600
H	−4.77687300	0.22212200	−1.59220200
O	−2.15362400	−1.32870200	1.67637000
H	−1.27393500	−1.70829000	1.66733800
H	−0.15478500	0.04923700	1.87794800
Cl	3.53317100	−0.72631000	−0.81828200
S	0.60921500	−1.98034000	−0.10802700
H	1.82405000	−2.52450900	0.00097900
H	−4.30315600	−1.23450600	0.35562600
IM26			
0	1		
C	−0.65923700	−0.51889200	−0.00309900
C	−1.49590900	0.56884300	−0.28136200
C	−2.87660400	0.41954700	−0.18546000
C	−3.43221700	−0.79178200	0.17117100
C	−2.61589100	−1.88124600	0.42735400
C	−1.24595000	−1.73494500	0.33941500
H	−3.51592500	1.25988500	−0.41029600
H	−4.50501400	−0.88633200	0.23789400
H	−3.04254600	−2.83375200	0.69926200

H	−0.59617600	−2.57137700	0.54909400
C	2.92224900	0.37327400	0.78419000
C	1.53738400	0.32690400	0.84379100
C	0.81586100	−0.43246300	−0.07997500
C	1.51972200	−1.15336600	−1.03914600
C	2.89923600	−1.11441900	−1.10114800
C	3.59641100	−0.34284600	−0.18411500
H	0.95949300	−1.74316400	−1.74950000
H	3.42447200	−1.67555400	−1.85744000
H	4.67413300	−0.29996300	−0.22052700
O	0.93970900	1.02878100	1.82276300
H	−0.01308100	0.94857900	1.75773500
S	−0.90616800	2.15626400	−0.76612800
H	0.35793500	1.78923700	−0.99256500
H	3.44740600	0.97030300	1.51259900
IM27			
0	2		
C	0.10119100	0.24626400	0.91723100
C	1.18888200	−0.38777500	0.09048400
C	2.43873400	0.15663200	0.04742200
C	2.78130900	1.31911600	0.75375100
C	1.80091300	1.97213800	1.52401300
C	0.53961100	1.49366800	1.60628800
H	3.78534500	1.70119600	0.68497200
H	2.07004100	2.87349100	2.05333800
H	−0.20804000	2.00394200	2.19400300
C	−3.41815100	0.04681900	−0.57032500
C	−2.31121600	−0.26037000	0.21346100
C	−1.14562200	0.49514600	0.08595200
C	−1.11224600	1.52153700	−0.84914100
C	−2.21217900	1.82206900	−1.62764900
C	−3.37461700	1.08509000	−1.48083000
H	−0.20045000	2.09276700	−0.95294900
H	−2.16287100	2.62660500	−2.34486600
H	−4.24322100	1.30781500	−2.08092300
H	−0.18340700	−0.47891600	1.68853800
Cl	3.69494000	−0.57648100	−0.89642200
S	−2.49749200	−1.56819600	1.40351400
H	−1.54153600	−2.37180800	0.92312500
H	−4.31208300	−0.54722800	−0.46343900
S	0.66333400	−1.81413300	−0.76425000
H	1.86912600	−2.35243400	−0.95478500

IM28			
0	1		
C	−0.73644800	0.56631600	−0.07626400
C	−1.56571700	−0.34605200	0.58410700
C	−2.94427400	−0.27272600	0.40882700
C	−3.50263300	0.69532500	−0.40153800
C	−2.69153400	1.61391600	−1.04609000
C	−1.32258300	1.54139700	−0.87712900
H	−3.58084900	−0.97697200	0.92303700
H	−4.57424700	0.73468200	−0.52175700
H	−3.12005900	2.37703600	−1.67633200
H	−0.67614000	2.24582100	−1.37870800
C	2.94214800	−0.27831300	−0.40821300
C	1.56306100	−0.35260900	−0.57926000
C	0.73668500	0.56386100	0.07950700
C	1.32578300	1.53961200	0.87770300
C	2.69548000	1.61380700	1.04070200
C	3.50394600	0.69429600	0.39427000
H	0.68104100	2.24551700	1.37954800
H	3.12620900	2.37875700	1.66719300
H	4.57602200	0.73353200	0.51046700
S	−0.97003100	−1.61462300	1.64662700
H	0.31325700	−1.24780500	1.63019900
H	3.57661100	−0.98871100	−0.91658700
S	0.96919500	−1.61116600	−1.65324400
H	−0.33195500	−1.36220900	−1.49092100
IM29			
0	2		
C	0.15941200	0.08381100	0.95115200
C	1.18343500	−0.52123700	0.05009400
C	2.42384300	0.00975000	−0.09660600
C	2.84915400	1.13944700	0.62116900
C	1.95416100	1.73083500	1.53059100
C	0.69786800	1.25435800	1.70405100
H	3.84377500	1.52326700	0.47388700
H	2.28466100	2.58459300	2.10247100
H	0.02446100	1.72355900	2.40461800
O	0.75576800	−1.61826000	−0.58731400
H	1.44524200	−1.94517600	−1.17072100
C	−3.32735200	−0.04208900	−0.60738500
C	−2.19610200	−0.42569700	0.10246400
C	−1.09770600	0.43180600	0.17000900
C	−1.15312000	1.64078800	−0.50974900

C	−2.27789800	2.01778700	−1.21929200
C	−3.37482800	1.17572700	−1.25898300
H	−0.29555900	2.29668600	−0.46420700
H	−2.29721500	2.96532600	−1.73510500
H	−4.26151800	1.45737200	−1.80557800
H	−0.13421000	−0.69124300	1.66959800
Cl	3.52219300	−0.76450300	−1.19795600
S	−2.26549800	−1.98249900	0.95046800
H	−1.16271000	−2.50641100	0.40979400
H	−4.16758800	−0.71707200	−0.65356800
IM30			
0	3		
C	0.57382200	0.42531500	−1.02824100
C	1.58263300	−0.46172700	−0.34391600
C	2.78051700	0.05117800	0.06352800
C	3.17320600	1.36753200	−0.21752400
C	2.32983500	2.19288100	−0.98700800
C	1.11830700	1.76186500	−1.40184500
H	4.13271500	1.71511500	0.12507700
H	2.67161400	3.18086300	−1.25534700
H	0.47895400	2.39421900	−1.99847100
C	−1.73986400	1.42201300	1.82385400
C	−0.60743000	1.30489000	1.01708900
C	−0.64918000	0.58337800	−0.14478200
C	−1.87537000	−0.06723000	−0.55729700
C	−3.02347500	0.09083300	0.31497300
C	−2.94557800	0.81657800	1.47216200
H	−1.68668900	1.99220700	2.73819100
H	0.31571300	1.78440300	1.31002200
H	−3.81378000	0.91634000	2.10323000
O	−1.94014600	−0.72446500	−1.60311600
H	0.21654200	−0.08710000	−1.92585700
Cl	−4.47907900	−0.66124700	−0.15075100
Cl	3.92214900	−0.92902300	0.92639000
S	1.01899200	−2.09118200	−0.13078400
H	2.06550100	−2.53185000	0.56878600
IM31			
0	2		
C	−2.52091100	0.14659200	−0.11616400
C	−2.88135300	1.22473600	0.64555100
C	−1.89016200	2.05469500	1.16235900
C	−0.53718300	1.80398300	0.92453900

C	−0.13321400	0.72396100	0.18245900
C	−1.13029700	−0.15423500	−0.40346500
H	−3.92295200	1.42383200	0.83859900
H	5.08195500	0.13835700	−0.79132000
C	4.02609600	0.24713300	−0.59779600
C	3.36201600	1.40231600	−0.96419300
C	3.33027900	−0.77933400	0.01658100
C	2.00857800	1.52093500	−0.70860300
C	1.97123100	−0.67111600	0.27233800
C	1.29553700	0.49868900	−0.09073500
H	1.48163200	2.41659700	−1.00214800
H	3.89070200	2.20799400	−1.44927100
O	−0.81738000	−1.10220500	−1.13202600
H	−2.17584400	2.90333300	1.76404900
H	0.20818600	2.45831300	1.35003100
Cl	−3.70083000	−0.88863300	−0.77700800
H	3.83970500	−1.68463200	0.30657000
S	1.20611400	−1.98061400	1.19196400
H	0.34823200	−2.35970300	0.24019200
IM32			
0	3		
C	2.46691400	2.29583600	−1.10854200
C	1.14409800	2.37827000	−0.68918400
C	0.63198200	1.29509000	−0.03650500
C	1.29983300	0.13049900	0.24756300
C	2.62577200	0.07971900	−0.18774500
C	3.19735700	1.14797200	−0.85473400
H	2.92450700	3.12199900	−1.63002700
H	0.55022400	3.26015400	−0.87341500
H	4.22356000	1.06756200	−1.17405100
O	0.80059000	−0.93907600	0.89260300
C	−0.56729000	−0.90307400	1.31357400
C	−1.49143100	−0.81707800	0.13142000
C	−2.38635200	0.21156200	0.04008100
C	−2.50848000	1.18725400	1.03605000
C	−1.70833200	1.11591800	2.19517500
C	−0.79495200	0.13478300	2.35069300
H	−3.22629300	1.97970700	0.90821400
H	−1.83656100	1.86370400	2.96252700
H	−0.17058200	0.07573300	3.22844200
Cl	3.55490600	−1.33880000	0.11662100
S	−1.21613500	−2.07059500	−1.03102200
H	−2.22832300	−1.75113200	−1.83982500

Cl	−3.43504100	0.36097600	−1.32920900
H	−0.67552200	−1.89309900	1.76057000
IM33			
0	2		
C	3.34473000	−1.63138400	0.89188000
C	2.08193100	−2.18290300	1.08437400
C	1.01972700	−1.46216900	0.62565300
C	1.08437900	−0.25402000	−0.01845600
C	2.36100300	0.27441100	−0.19889800
C	3.47530600	−0.40977700	0.25500700
H	4.22264700	−2.15241600	1.24037500
H	1.95249400	−3.13212800	1.58064000
H	4.44791100	0.02841000	0.10163500
O	0.01917200	0.44882800	−0.45425500
C	−1.21892200	−0.14069200	−0.40002900
C	−2.23195500	0.54540000	0.25699500
C	−3.50196400	−0.01912700	0.27364200
C	−3.74723100	−1.22980400	−0.34462400
C	−2.72848400	−1.89269200	−1.00856100
C	−1.46255400	−1.33928100	−1.04333200
H	−4.30167600	0.50217400	0.77727600
H	−4.74023000	−1.65000000	−0.31571200
H	−2.91802100	−2.82991700	−1.50699700
H	−0.65577200	−1.82507300	−1.56969700
Cl	2.53836700	1.79422500	−0.98884200
S	−1.96444400	2.07127200	1.08087200
H	−0.73767100	2.27837100	0.60097700
IM34			
0	3		
C	−0.65242900	0.09546300	1.01973300
C	−1.71595000	−0.51105600	0.14362300
C	−2.91870600	0.11565000	−0.01296700
C	−3.25662500	1.28453000	0.68416800
C	−2.34830300	1.82223500	1.61630300
C	−1.13114600	1.26713000	1.80768100
H	−4.22173200	1.73321700	0.52249000
H	−2.64224900	2.68781400	2.19013700
H	−0.44002600	1.67917100	2.52696200
C	1.40625000	1.89262000	−1.58273300
C	0.36065100	1.47510900	−0.77962400
C	0.54954500	0.49850300	0.17747800
C	1.82717100	−0.09465000	0.35648300

C	2.87595500	0.36711800	−0.48263800
C	2.66818100	1.33927200	−1.43412900
H	1.24413300	2.65410300	−2.32977500
H	−0.62273000	1.90832400	−0.89485100
H	3.48937500	1.66008300	−2.05355200
Cl	4.45961500	−0.27915300	−0.34143000
H	−0.30120100	−0.67566800	1.70949800
Cl	−4.13052800	−0.53439000	−1.07010900
S	2.09577500	−1.31237300	1.51246700
S	−1.20544500	−1.99338700	−0.60499200
H	−2.33288500	−2.23619400	−1.27458900
IM35			
0	2		
C	−2.45626800	0.23738700	−0.04364900
C	−2.78283500	1.40833300	0.61058200
C	−1.78035300	2.27994900	0.99240500
C	−0.45153500	1.97955000	0.72954700
C	−0.10701600	0.80933300	0.08101500
C	−1.11524700	−0.09922400	−0.33876600
H	−3.81749300	1.62989700	0.81465000
H	5.08045300	−0.03682900	−0.87906100
C	4.03219400	0.13012400	−0.68555500
C	3.33429300	1.09079500	−1.39560300
C	3.39256000	−0.60762400	0.29131800
C	1.99309500	1.29097000	−1.12768100
C	2.04306200	−0.41578500	0.55611700
C	1.32555000	0.53436400	−0.17343100
H	1.43629900	2.03359400	−1.67948300
H	3.82775400	1.68083900	−2.15167500
H	−2.03635300	3.19551900	1.50250600
H	0.32981600	2.65564000	1.03995900
Cl	−3.74278500	−0.80099200	−0.50198800
H	3.94547700	−1.33460000	0.86649100
S	−0.70257900	−1.52334700	−1.17815800
S	1.32920000	−1.43710400	1.80170900
H	0.27044800	−0.66901400	2.06577900
IM36			
0	3		
C	1.91233100	2.68414100	−0.53752800
C	0.76385400	2.40692800	−1.27087900
C	0.46420500	1.08405400	−1.41911700
C	1.15567100	0.01111100	−0.92230500

C	2.30927800	0.33606800	−0.19777500
C	2.67994600	1.65807700	−0.01247600
H	2.21730500	3.70797300	−0.38049100
H	0.15590100	3.19123600	−1.69257200
H	3.57467100	1.87699700	0.54670500
Cl	3.31335900	−0.89591700	0.46810000
C	−0.68219300	−1.76170200	0.22541100
C	−1.74610400	−0.74593800	0.07399500
C	−1.73172800	0.35349500	0.89382100
C	−0.84460600	0.46866700	1.96708600
C	0.02538900	−0.59222200	2.26813200
C	0.06533200	−1.69775900	1.48552000
H	−0.86757000	1.35828500	2.57344900
H	0.65164000	−0.52545900	3.14405000
H	0.72904800	−2.51724800	1.70944200
H	−1.06054400	−2.76134600	0.01767800
Cl	−2.85318400	1.65305500	0.66312500
S	0.58555000	−1.62487500	−1.20759300
S	−2.82205500	−1.02522700	−1.25996200
H	−3.66759700	−0.02968500	−0.99153100
IM37			
0	2		
C	−2.77969400	2.26039700	−0.60573800
C	−1.40286100	2.39052700	−0.75862400
C	−0.66578100	1.25597500	−0.59223000
C	−1.13475600	0.00698700	−0.28810200
C	−2.51928500	−0.08201000	−0.14072000
C	−3.33043300	1.02864100	−0.29888100
H	−3.42154500	3.11963200	−0.72480500
H	−0.94794300	3.33960500	−0.99451700
H	−4.39547500	0.91735400	−0.17764400
C	1.48755200	−0.66679700	−0.30124100
C	2.10588100	0.08564600	0.70358500
C	3.37874500	0.59519500	0.46665700
C	4.02934700	0.35704500	−0.72706500
C	3.42508500	−0.40426000	−1.71327300
C	2.15930100	−0.91004500	−1.49282900
H	3.86439500	1.17514100	1.23667000
H	5.01781000	0.76132800	−0.88186100
H	3.93105900	−0.60000800	−2.64520300
H	1.66591800	−1.49891300	−2.25019000
Cl	−3.23507400	−1.60529700	0.24524400
S	−0.10412400	−1.40853900	−0.08917100

S	1.37907900	0.46898200	2.25222800
H	0.36417300	−0.39498100	2.15025400
IM38			
0	3		
C	−0.77069800	0.18862500	1.01493900
C	−1.66065800	−0.56914900	0.08620500
C	−2.93340900	−0.17269600	−0.17396000
C	−3.51376600	0.95036800	0.43685500
C	−2.74590300	1.68403400	1.35921100
C	−1.46514900	1.34666000	1.64687500
H	−4.52815700	1.22263900	0.20203100
H	−3.19651000	2.53289200	1.85073700
H	−0.89347400	1.92052800	2.35997700
O	−1.07742800	−1.64063700	−0.45323000
H	−1.69200500	−2.08480700	−1.04236500
C	1.53517800	2.11853000	−1.27447800
C	0.43114100	1.71806000	−0.54084500
C	0.48743500	0.60414600	0.27100800
C	1.68205400	−0.15816700	0.36089100
C	2.78903700	0.28307800	−0.40719700
C	2.71788100	1.40187200	−1.20720100
H	1.47769000	2.99255600	−1.90461200
H	−0.49218400	2.27530100	−0.59675900
H	3.58314800	1.70570500	−1.77295700
Cl	4.28063900	−0.56609000	−0.36454900
H	−0.44486400	−0.51814800	1.78608900
Cl	−3.87268000	−1.11481200	−1.29061300
S	1.78447600	−1.54490000	1.34047700
IM39			
0	2		
C	−2.37685300	0.21814700	−0.01596800
C	−2.67851600	1.52530800	0.29842000
C	−1.65408100	2.44001900	0.47021700
C	−0.33140800	2.04662500	0.34588400
C	−0.00392500	0.73772200	0.04037400
C	−1.03749300	−0.22138300	−0.16933400
H	−3.70907700	1.82175400	0.40386300
H	0.44926300	−0.88609900	1.78809900
H	5.19399700	−0.40201800	−0.37639000
C	4.14179500	−0.17585100	−0.29735800
C	3.54916100	0.70193700	−1.19109000
C	3.39466200	−0.76579300	0.70164200

C	2.20102300	0.97650300	−1.07079300
C	2.04084500	−0.49245200	0.81360400
C	1.42512900	0.38059400	−0.08316000
O	1.38519600	−1.07660000	1.83438300
H	1.72199800	1.65307200	−1.76314500
H	4.12899000	1.16433900	−1.97374800
H	−1.89115400	3.46396600	0.71418200
H	0.46414900	2.75869100	0.49977900
Cl	−3.68491300	−0.86995600	−0.23016800
H	3.83904600	−1.44433500	1.41211100
S	−0.66608100	−1.80972400	−0.65760000
IM40			
0	3		
C	1.61533800	2.79096700	−0.09681500
C	0.54673900	2.55781000	−0.95550300
C	0.34509000	1.25367300	−1.30229600
C	1.06236900	0.15908000	−0.89782500
C	2.13590300	0.44242600	−0.04368700
C	2.40473900	1.74379500	0.34844000
H	1.84120700	3.79800800	0.22068000
H	−0.07507500	3.35980700	−1.31990500
H	3.23833000	1.93087200	1.00517500
Cl	3.16782900	−0.81282400	0.52848000
C	−0.72077500	−1.87874200	−0.13843300
C	−1.84300700	−0.94400800	−0.26163500
C	−1.99299900	0.06770500	0.64318900
C	−1.19983500	0.15635800	1.79079000
C	−0.27470800	−0.86529000	2.05805300
C	−0.08999300	−1.88885400	1.18360600
H	−1.34069400	0.97925400	2.47075500
H	0.27783000	−0.84108800	2.98433700
H	0.61371100	−2.67802700	1.39201700
O	−2.60962400	−1.11902400	−1.33667500
H	−3.25903000	−0.41433700	−1.39334300
H	−0.99925600	−2.86574200	−0.50128400
Cl	−3.24073200	1.23914800	0.36306900
S	0.61788500	−1.44584800	−1.45554200
IM41			
0	2		
C	−2.69217100	2.35164500	0.15250900
C	−1.31008700	2.50735100	0.11530400
C	−0.57620800	1.36515200	−0.00994500

C	−1.05572700	0.08680000	−0.10033900
C	−2.44503800	−0.02878100	−0.05930200
C	−3.25228600	1.08901400	0.06557300
H	−3.33180600	3.21523300	0.25009100
H	−0.84925500	3.48020600	0.18308600
H	−4.32176000	0.95899800	0.09314600
C	1.56514500	−0.54822700	−0.28190700
C	2.22712800	−0.33238100	0.92756800
C	3.48455700	0.25557700	0.92197200
C	4.07123700	0.61475400	−0.27334800
C	3.42411400	0.39302000	−1.48129400
C	2.17398000	−0.19085300	−1.47778100
H	3.98056500	0.41723000	1.86553800
H	5.04978900	1.06994500	−0.26329900
H	3.89081400	0.67101400	−2.41247900
H	1.64939100	−0.37596500	−2.40232500
O	1.69232700	−0.66635400	2.10772400
H	0.85044500	−1.10686500	1.95387000
Cl	−3.16832400	−1.59263700	−0.16415700
S	−0.01769600	−1.33140900	−0.27080100
IM42			
0	3		
C	−0.03190800	0.34913500	−1.07217700
C	1.05624600	−0.42185900	−0.36656500
C	2.15574400	0.23152400	0.11181500
C	2.38812700	1.59299800	−0.12709700
C	1.48930600	2.31799800	−0.93355700
C	0.36479800	1.74531700	−1.41616300
H	3.27517300	2.05386000	0.27260400
H	1.71822300	3.34481700	−1.17519600
H	−0.31744400	2.30212000	−2.03995000
C	−2.51072700	1.07740000	1.72098200
C	−1.36517700	1.10459900	0.92878400
C	−1.28224200	0.35040100	−0.21430900
C	−2.39898300	−0.48074900	−0.60452500
C	−3.56319800	−0.48127000	0.23916200
C	−3.60912700	0.28101200	1.36944100
H	−2.55192000	1.67719400	2.61707000
H	−0.52681300	1.72384600	1.21553400
H	−4.48686300	0.27440200	1.99696800
O	−2.35633600	−1.17287100	−1.63895400
H	−0.30439400	−0.19215000	−1.98235000
Cl	3.37158400	−0.61335700	1.01654700

S	0.69919300	−2.11436400	−0.20892500
H	1.76893200	−2.43471200	0.52096200
H	−4.38537100	−1.10856000	−0.06724900
IM43			
0	2		
C	−2.98826100	0.46425100	0.68690500
C	−3.62892600	−0.37533800	−0.17538500
C	−2.88139800	−1.25349000	−0.96911600
C	−1.48980900	−1.27859800	−0.89343900
C	−0.80163800	−0.43457700	−0.05250700
C	−1.55451300	0.46869900	0.79601900
H	−4.70529900	−0.36949000	−0.24896500
H	4.50635500	−0.92689800	0.24759400
C	3.43433300	−0.81893800	0.18988400
C	2.60653800	−1.89359800	0.45373900
C	2.88339800	0.40538300	−0.14494100
C	1.23537700	−1.73288700	0.37713300
C	1.50884800	0.57629000	−0.22086700
C	0.66526500	−0.50876200	0.04174900
H	0.58285100	−2.56597100	0.59243000
H	3.02201500	−2.85278900	0.72069200
O	−0.98817100	1.21658500	1.61386900
H	−3.38600600	−1.91757600	−1.65375600
H	−0.93526300	−1.95834100	−1.52284100
H	3.52095300	1.25029800	−0.35209900
S	0.91635900	2.14274800	−0.80296300
H	0.22407200	2.46194300	0.29362000
H	−3.52133700	1.15157000	1.32473200
IM44			
0	3		
C	−3.44141600	−0.94861700	−1.35111400
C	−2.15100500	−1.42656000	−1.15361900
C	−1.34425100	−0.73626900	−0.29384400
C	−1.70884400	0.39607200	0.39171000
C	−3.00365600	0.86147900	0.18185100
C	−3.85374300	0.19244600	−0.67908400
H	−4.11156000	−1.46369700	−2.02187900
H	−1.80021400	−2.31159300	−1.66300200
H	−4.85372300	0.56948100	−0.82739800
O	−0.93170800	1.09865700	1.24859700
C	0.37961900	0.61376200	1.53597400
C	1.25118400	0.63439000	0.31053200

C	1.87599900	−0.50806400	−0.10277900
C	1.75844400	−1.71767100	0.59256000
C	0.99148400	−1.78033400	1.77329000
C	0.33980800	−0.69440500	2.23815900
H	2.26729800	−2.58913500	0.21682300
H	0.93035600	−2.71842100	2.30306200
H	−0.26039000	−0.73513500	3.13362000
S	1.27947200	2.18586000	−0.46026900
H	2.18229700	1.88078100	−1.39347700
Cl	2.86116600	−0.52018600	−1.52729100
H	0.74576400	1.37979400	2.22346200
H	−3.31927700	1.75069000	0.70644200
IM45			
0	2		
C	4.00189700	−0.42091400	0.58918400
C	2.89051900	−1.02208700	1.17341200
C	1.66123700	−0.65572300	0.70765700
C	1.44256700	0.24620000	−0.30240800
C	2.55846200	0.83412100	−0.88250200
C	3.82399100	0.50036200	−0.43051300
H	4.99325900	−0.67378100	0.93170300
H	2.99969000	−1.74257300	1.96989600
H	4.68335400	0.96502100	−0.88804700
O	0.21562100	0.62491600	−0.74344400
C	−0.86633300	−0.15303000	−0.42733100
C	−1.98971200	0.47967400	0.09137900
C	−3.11443300	−0.29051800	0.36185300
C	−3.11577100	−1.65087700	0.12334500
C	−1.99179100	−2.26550600	−0.40295300
C	−0.86877900	−1.51171900	−0.68524500
H	−3.99352600	0.18791200	0.76599100
H	−3.99907500	−2.22910400	0.34449800
H	−1.98981000	−3.32526100	−0.60253500
H	0.01251500	−1.96575400	−1.11064000
S	−2.03869100	2.20218300	0.42299000
H	−0.81818400	2.46593600	−0.04298400
H	2.41473500	1.54344700	−1.68345000
IM46			
0	3		
C	0.06000300	−0.05248300	0.99142200
C	1.18186700	0.49166000	0.14686200
C	2.36681800	−0.17982100	0.06125000

C	2.63319600	−1.34009000	0.80304200
C	1.66563800	−1.82185900	1.70567200
C	0.46219600	−1.21916600	1.82798000
H	3.58810900	−1.82557700	0.69695500
H	1.90178300	−2.68191500	2.31355300
H	−0.27495400	−1.58829900	2.52452900
C	−1.99581200	−1.84177700	−1.63489600
C	−0.96218500	−1.45597200	−0.79919500
C	−1.12136300	−0.42619300	0.11045500
C	−2.36611000	0.24206300	0.18606500
C	−3.40541900	−0.17087000	−0.67385100
C	−3.22558000	−1.19539300	−1.57065000
H	−1.84381500	−2.64698500	−2.33726800
H	−0.00568600	−1.95758600	−0.85044700
H	−4.03212100	−1.49467900	−2.22143500
H	−0.28950200	0.75144700	1.64538600
Cl	3.64700100	0.39638100	−0.95832700
S	−2.65870300	1.53459300	1.26152200
H	−4.34904600	0.34694700	−0.60852400
S	0.76008000	1.96738700	−0.66794000
H	1.92468000	2.14588800	−1.29344200
IM47			
0	2		
C	2.93881200	−0.29464000	0.44145000
C	3.51498900	0.63984000	−0.38979800
C	2.70851400	1.54352800	−1.06597000
C	1.33082000	1.50101900	−0.91113600
C	0.73046600	0.56836300	−0.08296600
C	1.54611800	−0.34899200	0.62334500
H	4.58648700	0.66832300	−0.51197600
H	−4.58340500	0.70963100	0.42540000
C	−3.50940700	0.68147700	0.32435800
C	−2.72683500	1.66576200	0.90039000
C	−2.92086200	−0.34413500	−0.39105300
C	−1.35293800	1.60609700	0.75926000
C	−1.54112900	−0.40488400	−0.53434700
C	−0.74347600	0.57537000	0.05785300
H	−0.72818900	2.36217300	1.21076300
H	−3.18001400	2.47094900	1.45669100
H	3.14997700	2.28010300	−1.71959200
H	0.70622300	2.19836200	−1.44833600
H	−3.54122900	−1.10107700	−0.84683700
S	0.88761400	−1.48951200	1.71409900

H	3.54631100	−1.00284700	0.98223800
S	−0.75796700	−1.67288300	−1.47344600
H	−1.88094900	−2.32012900	−1.78710600
IM48			
0	3		
C	3.02646800	1.64625600	−0.37485800
C	1.98109700	1.63808100	−1.29374700
C	1.26565500	0.47836600	−1.38829400
C	1.47842300	−0.66292700	−0.65968400
C	2.53468100	−0.62726500	0.25238400
C	3.30019700	0.51888200	0.38547800
H	3.62982500	2.53453900	−0.26008800
H	1.75573500	2.50473200	−1.89550900
H	4.11679900	0.53383500	1.09049500
C	−0.93899100	−1.67321200	0.34278800
C	−1.52739100	−0.35804700	0.00511000
C	−1.21568000	0.72510500	0.78637300
C	−0.50481700	0.59929100	1.98175600
C	−0.14280800	−0.67675300	2.44602600
C	−0.40435100	−1.78059100	1.70617800
H	−0.27784200	1.48326700	2.55315300
H	0.32796800	−0.76916300	3.41248500
H	−0.14222900	−2.76613500	2.05661600
H	−1.62601600	−2.48287100	0.10319100
Cl	−1.72967100	2.31863900	0.34540400
S	0.47624300	−2.09333500	−0.87275100
H	2.74150900	−1.50526800	0.84613200
S	−2.43485500	−0.33319700	−1.47488800
H	−2.89844300	0.91160100	−1.35316700
IM49			
0	2		
C	3.66005200	0.18625600	1.33896300
C	2.35465400	0.24589500	1.81578900
C	1.36050700	−0.06864700	0.93404000
C	1.53309400	−0.43191400	−0.37307200
C	2.84923300	−0.48236300	−0.83264000
C	3.89463000	−0.17722700	0.02151900
H	4.48409800	0.42406100	1.99413600
H	2.14202200	0.52901400	2.83526500
H	4.90678400	−0.22323300	−0.34928200
C	−1.19916900	−0.57124500	−0.44798400
C	−1.67552300	0.70609600	−0.13168800

C	−2.82001300	0.81586800	0.65264900
C	−3.48535900	−0.30637700	1.10176900
C	−3.02511000	−1.57013900	0.77274700
C	−1.88615100	−1.69229900	0.00189500
H	−3.19471400	1.79740900	0.90036400
H	−4.37268300	−0.19060900	1.70485200
H	−3.54341500	−2.45140800	1.11565200
H	−1.50382700	−2.66699200	−0.25782800
S	0.21326300	−0.83235200	−1.47597100
H	3.04886000	−0.76191900	−1.85736200
S	−0.91663800	2.20528800	−0.63008000
H	−0.07820800	1.65327200	−1.51291100
IM50			
0	3		
C	−0.14415700	−0.10033600	0.94704800
C	−1.17837200	−0.56384100	−0.02369100
C	−2.41942800	−0.01469900	−0.07007000
C	−2.82927500	1.00036400	0.80958300
C	−1.91818000	1.45473300	1.78024100
C	−0.66219800	0.95290800	1.86736400
H	−3.82498600	1.40222800	0.73647200
H	−2.23594000	2.21825200	2.47399000
H	0.02165100	1.31177700	2.62110600
O	−0.75521200	−1.54513200	−0.82220100
C	2.13946800	2.03610200	−1.18811400
C	1.05457200	1.57709400	−0.45944100
C	1.09064200	0.35845000	0.19244100
C	2.26166000	−0.43203500	0.11164400
C	3.35056200	0.05279000	−0.64128300
C	3.29443900	1.26882900	−1.27910300
H	2.08348800	2.99169600	−1.68672100
H	0.15666000	2.17461600	−0.39029900
H	4.14017400	1.62269200	−1.84764500
H	0.16346300	−0.97896100	1.52368900
Cl	−3.53924200	−0.61773500	−1.25385000
S	2.41805700	−1.94120900	0.89429800
H	4.23476100	−0.56165300	−0.70144100
H	−1.45777200	−1.80089200	−1.42455700
IM51			
0	2		
C	2.89621300	0.44054500	−0.17693500
C	3.44812700	−0.76103200	0.19976000

C	2.61567400	−1.83885600	0.47296600
C	1.23962300	−1.69997900	0.38436600
C	0.65586200	−0.49713400	0.01595900
C	1.50133700	0.60232700	−0.29129500
H	4.51894400	−0.86599700	0.27661600
H	−0.01298700	1.16929300	1.64453200
H	−4.67342300	−0.31669900	−0.22598000
C	−3.59571900	−0.35111100	−0.18171900
C	−2.89089700	−1.19749000	−1.02319400
C	−2.92781200	0.45063400	0.72086600
C	−1.51215700	−1.22454900	−0.94954800
C	−1.54389800	0.42397700	0.78646600
C	−0.81642900	−0.41239800	−0.06088300
O	−0.96571400	1.20219700	1.72125900
H	−0.94722000	−1.87232400	−1.60373500
H	−3.40850700	−1.82554400	−1.73051400
H	3.03811800	−2.78771400	0.76604900
H	0.59737300	−2.53582400	0.61651100
H	−3.45828000	1.10718800	1.39180000
S	0.88316300	2.08828700	−0.86727800
H	3.52155200	1.28786100	−0.40976000
IM52			
0	3		
C	2.86574100	1.81492400	0.02374800
C	1.91001600	1.85140200	−0.98739900
C	1.26989600	0.67518500	−1.25876700
C	1.47375300	−0.52289700	−0.62541200
C	2.44236500	−0.52945800	0.38019300
C	3.12937300	0.63034200	0.69548400
H	3.40732900	2.71375300	0.27891600
H	1.69379900	2.76227500	−1.52376200
H	3.87640600	0.61169200	1.47373500
C	−0.95262500	−1.77005900	0.07564100
C	−1.67133800	−0.54556200	−0.29483800
C	−1.56819800	0.57125600	0.48310500
C	−0.92979800	0.55004800	1.72630600
C	−0.44525400	−0.67200900	2.22029500
C	−0.51556100	−1.80606200	1.47541000
H	−0.85670600	1.45704700	2.30173800
H	−0.03262600	−0.70490600	3.21671400
H	−0.15533700	−2.74754000	1.85826900
O	−2.32524900	−0.60316800	−1.45349600
H	−2.67999900	0.26203700	−1.67073100

H	−1.51671500	−2.64965300	−0.22794000
Cl	−2.29621500	2.03857600	−0.08618800
S	0.57562600	−1.97285900	−1.06258600
H	2.64127800	−1.45208200	0.90499500
IM53			
0	2		
C	−3.61025700	1.32963900	0.27141900
C	−2.30145700	1.78241800	0.40337300
C	−1.30857300	0.87710100	0.15922100
C	−1.48625200	−0.42943400	−0.20385900
C	−2.80511800	−0.86303900	−0.33392000
C	−3.84925700	0.01386900	−0.09503100
H	−4.43381300	2.00303300	0.45337000
H	−2.08570100	2.80106700	0.68720000
H	−4.86422100	−0.33644500	−0.20069600
C	1.24318000	−0.49653400	−0.36065500
C	1.83877300	−0.32328800	0.88953100
C	2.95886200	0.48842100	1.00620800
C	3.47648200	1.11156300	−0.10989700
C	2.89502900	0.93770000	−1.35841300
C	1.78159900	0.13110700	−1.47609100
H	3.40466200	0.61283100	1.98000900
H	4.34830100	1.73923200	−0.00560000
H	3.30702500	1.42424900	−2.22780100
H	1.31058900	−0.02229100	−2.43460900
O	1.36649900	−0.91035500	1.99514900
H	0.62016600	−1.46781600	1.75155400
S	−0.15805800	−1.55901600	−0.50570600
H	−3.00814200	−1.88390100	−0.62464000
IM54			
0	2		
C	2.42023900	2.43723000	0.65557300
C	1.19494300	2.61588000	0.03654900
C	0.58610700	1.55782100	−0.61238800
C	1.18427300	0.30753800	−0.63627600
C	2.42165100	0.14305700	−0.02403300
C	3.03831200	1.20129900	0.61797200
H	2.90288200	3.26000500	1.15902000
H	0.71646100	3.58261600	0.04674100
H	3.99920500	1.04716100	1.08180300
O	0.62138000	−0.72812300	−1.30889100
C	−0.49070900	−1.39119100	−0.68007600

C	−1.71716000	−0.54587600	−0.72219300
C	−2.37378500	−0.17762200	0.41207500
C	−1.96575100	−0.60111100	1.68105600
C	−0.85095700	−1.45384300	1.78515200
C	−0.15981000	−1.85457800	0.69196900
H	−2.50707900	−0.27434500	2.55226500
H	−0.55062700	−1.79639000	2.76338400
H	0.69511400	−2.50543900	0.78077700
H	−0.34948700	1.68819800	−1.13237900
H	−0.64348500	−2.23838700	−1.35075500
Cl	3.21254800	−1.38726400	−0.08596400
Cl	−3.76970900	0.84205000	0.25507800
O	−2.07549900	−0.18014200	−1.95259200
H	−2.88240200	0.33970200	−1.91620700
IM55			
0	1		
C	−3.25778000	1.71848200	0.42772600
C	−2.01395800	2.23906000	0.73860300
C	−0.87711600	1.46357100	0.60370500
C	−0.97893900	0.15942600	0.14950600
C	−2.22863100	−0.36407900	−0.15703500
C	−3.36258100	0.41319000	−0.01689300
H	−4.14555400	2.32148100	0.53123900
H	−1.92223900	3.25333200	1.09389500
H	−4.32097800	−0.01482800	−0.26265800
O	0.08899000	−0.67133700	0.03061600
C	1.34596500	−0.15376400	−0.16326300
C	2.33583000	−0.67821600	0.65627500
C	3.64394200	−0.25944300	0.48534600
C	3.94663000	0.66741500	−0.49516000
C	2.95362400	1.17836700	−1.31637100
C	1.64510000	0.75888300	−1.15336900
H	4.40560700	−0.67152300	1.12770800
H	4.96843000	0.98953200	−0.62094100
H	3.19536300	1.89407800	−2.08558500
H	0.85198100	1.13529900	−1.78100100
H	0.09531300	1.86074500	0.84747100
Cl	−2.36109100	−1.98931900	−0.70734500
O	2.03565900	−1.58109100	1.60416300
H	1.10091200	−1.78762800	1.54207800
IM56			
0	2		

C	3.37425700	−1.27971600	−0.80262200
C	3.32417400	0.05998700	−0.82625500
C	2.34740400	0.77633400	−0.01342700
C	1.13147800	−0.03542000	0.42133500
C	1.42736300	−1.47834500	0.61503800
C	2.45322800	−2.05976100	−0.00822700
C	0.07071400	0.14351900	−0.78784600
C	−1.18950300	−0.58415500	−0.50163000
C	−0.16825000	1.58005400	−1.07844000
C	−2.35657500	0.07463500	−0.25929900
C	−1.35650400	2.18469800	−0.83657300
C	−2.47153400	1.46179000	−0.38274700
O	2.44818000	1.95241000	0.23490000
H	−3.41027900	1.94332300	−0.16945200
H	0.74310700	−2.04363400	1.22400800
H	0.66324900	2.16068000	−1.44020200
H	4.15155700	−1.79702000	−1.34593700
H	−1.45735200	3.24456200	−1.01072300
H	2.61840800	−3.12075100	0.09120900
H	4.05959800	0.65990100	−1.33808700
H	0.57202500	−0.35444200	−1.62428200
Cl	0.43323300	0.64886500	1.89687700
Cl	−3.75631400	−0.86895500	0.14417500
O	−1.07743700	−1.91579000	−0.48952000
H	−1.93138700	−2.30577800	−0.28330900
IM57			
0	2		
C	−2.81256700	1.60153400	−0.14896600
C	−2.88726800	0.26756600	−0.02626200
C	−1.81250300	−0.48312000	0.63321700
C	−0.48978800	0.22925100	0.75739200
C	−0.58714200	1.70607400	0.79055700
C	−1.66809400	2.33871200	0.32812500
C	0.40342400	−0.24655800	−0.50373200
C	1.73907900	0.39674700	−0.44961100
C	0.50641800	−1.72843700	−0.52715800
C	2.84136500	−0.29480500	−0.04643200
C	1.63406200	−2.37651800	−0.14317200
C	2.81293800	−1.67790000	0.16480800
O	−1.94734300	−1.62489700	1.00408200
H	3.70371400	−2.18734500	0.49024300
H	−0.12200800	0.13446200	−1.38184100
H	0.26185300	2.26404800	1.14906100

H	−0.37884300	−2.28848700	−0.77573400
H	−3.64418200	2.14079900	−0.57646300
H	0.01569500	−0.16841000	1.63392600
H	1.63838600	−3.45413700	−0.09212100
H	−1.71289100	3.41603700	0.31079100
Cl	−4.28065900	−0.61300900	−0.49771600
Cl	4.34183200	0.56634600	0.10247200
O	1.75541800	1.68940900	−0.78881500
H	2.65418500	2.02493600	−0.73343800
IM58			
0	1		
C	−2.59795100	1.33805100	0.03454200
C	−2.32768200	0.03839600	−0.16014500
C	−1.07108500	−0.54364500	0.30414600
C	−0.04383700	0.38555100	0.91780200
C	−0.47566700	1.79336300	1.09591900
C	−1.66904800	2.22722600	0.69227900
C	1.28710900	0.27340500	0.19818800
C	2.09576500	−0.84126400	0.42642600
C	1.74460200	1.27508200	−0.64566200
C	3.36245700	−0.90129100	−0.13227800
C	2.99863500	1.20314000	−1.22486300
C	3.81214800	0.11662700	−0.95062800
O	−0.84184400	−1.73321000	0.22413600
H	4.79718900	0.05373400	−1.38698200
H	0.22615400	2.45998700	1.57476300
H	1.11134800	2.12854000	−0.83921500
H	−3.54367400	1.73184600	−0.30567700
H	0.12330000	−0.03083600	1.91960700
H	3.33804400	1.99234500	−1.87678800
H	−1.96765100	3.25224600	0.84415900
H	3.97043500	−1.76668500	0.07774900
Cl	−3.43213400	−1.01470300	−0.93563400
O	1.69547500	−1.85487300	1.21925300
H	0.76942500	−2.05441400	1.01671500
IM59			
0	1		
C	2.98440600	−0.62010000	1.31855800
C	2.72912500	−1.22860000	0.15366400
C	1.55992900	−0.88408500	−0.63772300
C	0.63934700	0.23565000	−0.12267100
C	1.04624600	0.82769400	1.18546200

C	2.12175400	0.41153000	1.85022400
C	−0.80810800	−0.17775500	−0.04813300
C	−1.80611700	0.76844900	0.21770600
C	−1.18016400	−1.51682700	−0.12544200
C	−3.11628400	0.36874600	0.41716200
C	−2.48742500	−1.91448400	0.07640700
C	−3.45679900	−0.96686900	0.35438100
O	1.30656600	−1.43088200	−1.68315700
H	−4.48173000	−1.26513500	0.51201800
H	0.40028700	1.59126300	1.59015200
H	−0.43790500	−2.26272300	−0.35132400
H	3.86002900	−0.89750600	1.88678200
H	−2.74316900	−2.95985200	0.01192600
H	2.36279100	0.84476300	2.80807300
H	−3.85126300	1.13259300	0.61426000
H	3.36849200	−1.99356700	−0.25651100
Cl	0.88326300	1.55009900	−1.38664200
O	−1.56001900	2.09247400	0.32711300
H	−0.82083600	2.34565000	−0.23167100
IM60			
0	2		
C	−0.62811100	0.44736500	−0.89563500
C	−1.66973200	−0.51769800	−0.43715800
C	−2.89436600	−0.10789900	−0.01698900
C	−3.28579600	1.24116300	−0.03110000
C	−2.37876500	2.19749000	−0.52188500
C	−1.14190600	1.84928500	−0.95195800
H	−4.26695200	1.51660300	0.31498100
H	−2.68495300	3.23187600	−0.55947400
H	−0.45837400	2.59614700	−1.32494800
C	2.92637100	−0.06537400	0.34063200
C	1.83077100	−0.00587400	−0.51392600
C	0.59463500	0.36249500	0.00320900
C	0.48901800	0.66738500	1.34899900
C	1.58363800	0.60327300	2.19133400
C	2.81461500	0.23190200	1.68262400
H	−0.47852000	0.96035300	1.73205900
H	1.48375100	0.84092500	3.23808300
H	3.68736100	0.17267400	2.31248200
O	1.92837500	−0.28485600	−1.82365100
H	2.82603800	−0.54991100	−2.03141000
H	−0.30914900	0.13045700	−1.89179800
Cl	4.45731000	−0.53103800	−0.32690900

Cl	−4.01838000	−1.31377300	0.53341300
O	−1.26976400	−1.79003800	−0.46929000
H	−1.97212100	−2.35926300	−0.14590100
IM61			
0	1		
C	−1.37951900	0.28371500	−0.05912800
C	−1.96784300	−0.85745900	0.49581400
C	−3.33849900	−1.05094500	0.39441600
C	−4.13298600	−0.14014100	−0.26958700
C	−3.56355600	0.98162500	−0.85096300
C	−2.20316300	1.18120300	−0.73746000
H	−3.75477400	−1.93546300	0.84967500
H	−5.19601400	−0.31133900	−0.34263100
H	−4.17172000	1.69111900	−1.38933400
H	−1.74930700	2.05009600	−1.19115300
C	2.40021000	0.12145900	−0.04665000
C	1.07561800	−0.27013200	−0.17963700
C	0.04776600	0.62718000	0.10192300
C	0.40528900	1.90852900	0.50887700
C	1.72611300	2.29541500	0.63416900
C	2.73767200	1.39628300	0.35463100
H	−0.38335100	2.60561400	0.74609300
H	1.97036500	3.29399800	0.95881800
H	3.77624900	1.66822800	0.44830600
Cl	3.63461400	−1.03688700	−0.41609500
O	0.74537200	−1.51708600	−0.58282200
H	1.54007800	−2.01462000	−0.78986500
O	−1.27547700	−1.77725700	1.19814600
H	−0.42173200	−1.92619600	0.78268400
IM62			
0	3		
C	−0.59027900	0.69042700	0.53181100
C	−1.82911400	−0.12579800	0.64320400
C	−2.94120100	0.15326400	−0.08470000
C	−3.01667800	1.25029000	−0.95789300
C	−1.90679300	2.10918600	−1.05302200
C	−0.76884000	1.88426500	−0.35510700
H	−3.91905200	1.42632000	−1.51733800
H	−1.97550700	2.97561300	−1.69298000
H	0.07145400	2.55729700	−0.41527100
C	1.61807200	−1.97126300	−1.08272200
C	0.48007400	−1.29034400	−0.64248200

C	0.59371500	−0.12452700	0.06081800
C	1.90328600	0.42352000	0.35225400
C	3.05042600	−0.32174000	−0.12836400
C	2.89971600	−1.48923100	−0.82612000
H	1.50553900	−2.89107400	−1.63516700
H	−0.49741000	−1.69396400	−0.85766500
H	3.76837000	−2.02543000	−1.17221600
O	2.02878800	1.48211200	0.97929000
H	−0.32295900	1.04196400	1.53531500
Cl	4.59735700	0.30620900	0.20985900
Cl	−4.32666800	−0.87556300	0.11359100
O	−1.73105000	−1.13657800	1.51402300
H	−2.56833800	−1.60427700	1.56410900
IM63			
0	2		
C	−2.47286300	0.11615700	−0.05326800
C	−2.75834200	1.40405300	0.28324000
C	−1.70385800	2.27967700	0.55852800
C	−0.38289300	1.87072100	0.47251700
C	−0.03280700	0.57747000	0.11951100
C	−1.10744700	−0.37337400	−0.12518400
H	−3.78234000	1.73397800	0.34612900
H	5.23458700	−0.20099500	−0.27986100
C	4.16483600	−0.07028900	−0.22326000
C	3.57614500	1.10373300	−0.67757600
C	3.38129100	−1.08216400	0.27889500
C	2.21319100	1.24572900	−0.58010000
C	1.99596600	−0.95964500	0.34775100
C	1.38350200	0.24509700	−0.05046200
H	1.75046000	2.14873300	−0.94799200
H	4.17441900	1.89089200	−1.10744400
O	−0.89588900	−1.56489600	−0.39613600
H	−1.92644900	3.29415400	0.85021600
H	0.39786200	2.57476500	0.71101800
Cl	−3.71825900	−1.00052900	−0.38079300
H	3.80998400	−2.01019900	0.62148700
O	1.34056400	−2.00059400	0.86021100
H	0.45414200	−2.04140700	0.46378200
IM64			
0	3		
C	2.33115500	2.51847800	−0.05680300
C	0.98596000	2.37304300	0.26182600

C	0.50026600	1.10014600	0.33775500
C	1.21357600	−0.05401200	0.12945300
C	2.56247900	0.12778900	−0.18611100
C	3.10891200	1.39498500	−0.27730100
H	2.76940200	3.50140400	−0.13226100
H	0.35492200	3.23018200	0.43952000
H	4.15388300	1.48967900	−0.52363600
O	0.74103600	−1.30949100	0.19396000
C	−0.62076100	−1.51676700	0.58678900
C	−1.56815600	−0.92603100	−0.40203700
C	−2.45547300	0.04113600	−0.04312600
C	−2.56019400	0.50125700	1.27518900
C	−1.76394400	−0.09254900	2.27532300
C	−0.86772900	−1.06133100	1.97939900
H	−3.26259500	1.28283400	1.50992900
H	−1.88300300	0.23800800	3.29559500
H	−0.25547300	−1.51153400	2.74475000
Cl	3.55241600	−1.25513900	−0.46102000
Cl	−3.48299500	0.70827600	−1.26887800
H	−0.69895300	−2.60237600	0.51622500
O	−1.43152600	−1.41570800	−1.63048300
H	−2.03095700	−0.96680200	−2.23180000
IM65			
0	2		
C	−3.14222400	1.82338100	0.55400900
C	−1.85333600	2.32772400	0.69687400
C	−0.82512300	1.47432100	0.42653700
C	−0.94757600	0.17329900	0.01362500
C	−2.24872900	−0.30525100	−0.12358800
C	−3.33072900	0.51435100	0.14726000
H	−3.99530800	2.45018700	0.76149700
H	−1.67889500	3.34388400	1.01480300
H	−4.32414800	0.11250300	0.03326500
O	0.08428400	−0.65979300	−0.23881700
C	1.35990300	−0.14250100	−0.24910000
C	2.27703200	−0.81873600	0.54356600
C	3.59473500	−0.39699700	0.55508900
C	3.97824200	0.68048600	−0.22255700
C	3.05886100	1.34018500	−1.02211300
C	1.73993200	0.92002000	−1.04163800
H	4.29992800	−0.92456900	1.17704200
H	5.00753000	1.00309400	−0.20706800
H	3.36541400	2.17199800	−1.63555300

H	1.00788800	1.40411100	−1.66887500
Cl	−2.49908000	−1.93203200	−0.62913000
O	1.89882200	−1.86701500	1.29233200
H	0.97116900	−2.04389100	1.12420100
IM66			
0	2		
C	0.04794800	0.32929200	0.98269800
C	1.10369400	−0.48093700	0.31077100
C	2.25753500	0.05688200	−0.15320800
C	2.57332700	1.41346600	0.02916300
C	1.66963500	2.22070000	0.74441400
C	0.49446200	1.73406500	1.21088900
H	3.49856800	1.80440900	−0.35733800
H	1.92819000	3.25294500	0.92530900
H	−0.18610200	2.37021000	1.75551000
C	−3.39466700	−0.80918400	−0.19143500
C	−2.15503600	−0.80268700	0.42793800
C	−1.27325600	0.25879600	0.22687300
C	−1.64396900	1.26993600	−0.64808800
C	−2.87179900	1.25956700	−1.28314700
C	−3.75370000	0.21997500	−1.03977100
H	−0.95668500	2.08588700	−0.81587000
H	−3.14051600	2.05983200	−1.95443000
H	−4.72035800	0.20233700	−1.51930800
O	−1.86611300	−1.83325000	1.25685700
H	−0.94004300	−2.06495400	1.16903600
H	−0.13828400	−0.13882800	1.95929600
Cl	3.38433700	−0.98576000	−0.96396100
H	−4.05539100	−1.63957500	0.00104700
O	0.78795300	−1.78309200	0.21481700
H	1.47712900	−2.26282900	−0.25188200
IM67			
0	1		
C	−0.73859400	0.32969600	−0.03057300
C	−1.47231300	−0.68656800	0.58652100
C	−2.85866600	−0.68707500	0.53342600
C	−3.52224600	0.33095100	−0.12009700
C	−2.81380500	1.35940800	−0.72384800
C	−1.43395800	1.34894700	−0.67520000
H	−3.39219600	−1.48627300	1.02277400
H	−4.60083600	0.32398700	−0.15397300
H	−3.33260200	2.15633500	−1.23231600

H	−0.86558600	2.13468200	−1.15032800
C	2.85865000	−0.68706700	−0.53348000
C	1.47226800	−0.68661700	−0.58643400
C	0.73857300	0.32966200	0.03066200
C	1.43397500	1.34892100	0.67525300
C	2.81380900	1.35940500	0.72378900
C	3.52225100	0.33096500	0.11996100
H	0.86563200	2.13465900	1.15041200
H	3.33265200	2.15634800	1.23218800
H	4.60084200	0.32408700	0.15378000
O	0.87789500	−1.69502600	−1.25301700
H	−0.06669500	−1.54077900	−1.31313400
H	3.39207900	−1.48630900	−1.02287600
O	−0.87784400	−1.69500400	1.25298500
H	0.06663400	−1.54027300	1.31384100
IM68			
0	3		
C	−0.01124800	0.81182200	0.56044400
C	−1.11944100	−0.18340400	0.61790900
C	−2.28254800	−0.00504100	−0.05968500
C	−2.54005000	1.14121100	−0.82920200
C	−1.55958000	2.14856600	−0.88130300
C	−0.37824000	2.02693300	−0.22899200
H	−3.47794600	1.23741300	−1.34821200
H	−1.76423700	3.04347600	−1.44906200
H	0.35895000	2.81351700	−0.27008800
C	2.42560400	−1.04777900	−1.69304800
C	1.25621000	−0.47557400	−1.19830500
C	1.24555400	0.17287100	0.01123000
C	2.46834000	0.27734800	0.77470600
C	3.65375900	−0.32895200	0.23165900
C	3.62384400	−0.97354900	−0.96964700
H	2.40771400	−1.55383200	−2.64598600
H	0.34135200	−0.54165900	−1.77076900
H	4.51798800	−1.42589600	−1.36981200
O	2.49961200	0.87791600	1.86543000
H	0.23730000	1.10057800	1.58687800
Cl	−3.50115200	−1.23765800	0.06351900
H	4.55459100	−0.24705900	0.81913900
O	−0.84435600	−1.24370100	1.38096300
H	−1.59139200	−1.84677400	1.38235800
IM69			

0	2		
C	3.00294900	0.67586300	−0.31044200
C	3.56333800	−0.50008000	0.06882200
C	2.72959100	−1.56172100	0.46232500
C	1.35447700	−1.43207100	0.43920300
C	0.72002200	−0.25894300	0.03984300
C	1.57665600	0.86332400	−0.31279700
H	4.63519500	−0.62080400	0.08377200
H	−4.60880800	−0.61099000	−0.04692000
C	−3.53384600	−0.51529500	−0.05630900
C	−2.73674500	−1.58757200	−0.44169600
C	−2.95398100	0.68112100	0.29036600
C	−1.37183800	−1.43977400	−0.43004000
C	−1.57075500	0.85096900	0.26849100
C	−0.73960800	−0.24190900	−0.05593600
H	−0.75289700	−2.26435300	−0.74855100
H	−3.17901500	−2.52002300	−0.75371800
O	1.12145000	1.98298800	−0.62489600
H	3.16695500	−2.49012200	0.79548400
H	0.74937900	−2.26193200	0.76759300
H	−3.54844800	1.53477700	0.57370000
H	3.59070200	1.53155000	−0.60227600
O	−1.12515600	2.05259500	0.62152500
H	−0.26497600	2.21376800	0.18691200
IM70			
0	3		
C	−3.29582200	−1.57822100	0.44798400
C	−1.99933600	−1.50913000	0.94502700
C	−1.22107000	−0.46084900	0.54221300
C	−1.62037400	0.53557000	−0.31398300
C	−2.92082800	0.44976000	−0.80415100
C	−3.74208200	−0.59558100	−0.42298000
H	−3.94492300	−2.38940800	0.73945000
H	−1.62263300	−2.25866000	1.62483400
H	−4.74661300	−0.64172200	−0.81418700
O	−0.87041400	1.58351400	−0.72175100
C	0.47212000	1.69274800	−0.24591300
C	1.31025900	0.55273000	−0.71874700
C	1.94210500	−0.27636600	0.15553700
C	1.87081200	−0.08918100	1.54131100
C	1.16975900	1.02361500	2.04771900
C	0.53006800	1.88778700	1.22685000
H	2.36695400	−0.78400900	2.19728000

H	1.15222100	1.18185100	3.11507000
H	−0.01096900	2.73505200	1.61773900
Cl	2.85833200	−1.60178200	−0.48447700
H	0.81391300	2.59075600	−0.76346100
H	−3.26324400	1.21622300	−1.48303800
O	1.34930500	0.43243100	−2.04290100
H	1.85884300	−0.34464800	−2.28556700
IM71			
0	2		
C	3.81279700	−0.64565800	0.47893500
C	2.66207700	−1.22649600	1.00476700
C	1.46309800	−0.68844400	0.63679600
C	1.31038900	0.36835500	−0.22365800
C	2.46380300	0.93479000	−0.74830700
C	3.70164700	0.42787100	−0.39032100
H	4.78291900	−1.03309300	0.74878400
H	2.71959100	−2.06380300	1.68371100
H	4.59138300	0.87665900	−0.80341800
O	0.11292300	0.91176800	−0.56686200
C	−1.02864200	0.17932000	−0.34572100
C	−2.06520600	0.86907100	0.26867500
C	−3.27219100	0.22717800	0.47855500
C	−3.43125700	−1.08640900	0.07507300
C	−2.39465300	−1.76612300	−0.54356000
C	−1.18653100	−1.12597100	−0.76185200
H	−4.06937000	0.77084700	0.95976500
H	−4.37497200	−1.58089300	0.24433000
H	−2.52477500	−2.78765500	−0.86277200
H	−0.36807000	−1.62922100	−1.25251700
H	2.37099300	1.76335300	−1.43398400
O	−1.90346900	2.14549900	0.65661200
H	−1.01530900	2.42076100	0.42181100
